# The Exposome–Brain Axis: A Scoping Review of Biological Biomarkers in Environmental Neurotoxicity, Neuroinflammation, and Neurodegeneration

**DOI:** 10.3390/toxics14090817

**Published:** 2026-09-14

**Authors:** Olivia Curzio, Gabriele Donzelli, Said Daoudagh, Silvia Baldacci, Elisa Bustaffa, Chiara Cavigli, Maria Morales-Suárez-Varela, Paolo Paradisi, Davide Moroni, Fabrizio Minichilli

**Affiliations:** 1 Institute of Clinical Physiology, National Research Council, Via Moruzzi 1, 56124 Pisa, Italy; olivia.curzio@cnr.it (O.C.); silvia.baldacci@cnr.it (S.B.); elisa.bustaffa@cnr.it (E.B.); chiaracavigli@cnr.it (C.C.); 2 Institute of Information Science and Technologies “Alessandro Faedo”, National Research Council, Via Moruzzi 1, 56124 Pisa, Italy; said.daoudagh@cnr.it (S.D.); paolo.paradisi@cnr.it (P.P.); davide.moroni@cnr.it (D.M.); 3 Department of Preventive Medicine and Public Health, Food Sciences, Toxicology and Forensic Medicine, Faculty of Pharmacy, Universitat de València, Av. Vicent Andrés Estelles s/n, Burjassot, 46100 Valencia, Spain; maria.m.morales@uv.es; 4 Biomedical Research Center in Epidemiology and Public Health Network (CIBERESP), Carlos III Health Institute, Av. Monforte de Lemos 3-5 Pabellón 11 Planta 0, 28029 Madrid, Spain

**Keywords:** environmental neurotoxicology, exposome, biological biomarkers, neuroinflammation, air pollution, human biomonitoring

## Abstract

The escalating global burden of neurodegenerative and neuropsychiatric disorders is deeply intertwined with cumulative environmental toxicant exposure. To accurately capture the prodromal and subclinical impacts of this multi-chemical “pollutome”, research is shifting from symptom-based assessments toward the use of objective, quantifiable biological indicators. Guided by the PRISMA-ScR framework, this scoping review systematically analyzed literature across PubMed, Scopus, Web of Science, and Embase databases without temporal restrictions. The final selection included 52 human observational studies that evaluated the relationship between environmental stressors and objective neurobiological markers across diverse geographical populations and life stages. The synthesized evidence reveals that varied environmental insults—including ambient air pollution (e.g., fine particulate matter, PM_2.5_), heavy metals, agrochemicals, and persistent organic contaminants—frequently correlate with measurable alterations in fluid biomarkers. These exposures are primarily associated with variations in markers of axonal damage (neurofilament light chain), astrocytic reactivity (glial fibrillary acidic protein), potential microglial dysfunction (soluble triggering receptor expressed on myeloid cells 2), and cytostructural alterations (Tau proteins and amyloid-beta). The literature highlights distinct windows of vulnerability, spanning from early-life epigenetic modifications (DNA methylation) to adult neurovascular injury. Mechanistically, despite their chemical heterogeneity, the available evidence suggests that these pollutants may converge on shared pathophysiological pathways defined by blood–brain barrier disruption and chronic, self-perpetuating neuroinflammation. Understanding environmental neurotoxicity may benefit from moving beyond traditional single-pollutant approaches toward a broader exposome framework. Future epidemiological research should integrate high-dimensional human biomonitoring with artificial intelligence and machine learning architectures. This computational integration would be essential to decode non-linear multi-pollutant interactions and accelerate the deployment of targeted, early-stage public health interventions.

## 1. Introduction

The escalating prevalence of neuropsychiatric and neurodegenerative disorders represents a critical global public health challenge, acting as a major cause of death and disability worldwide [1,2]. In Europe, these conditions constitute the third most common cause of premature death and disability, a burden constantly exacerbated by population ageing. These multi-determinate disorders arise from complex interactions between genetic, biological, psychological, social, and environmental factors, driving premature mortality and a substantial economic impact [3,4,5].

Among environmental stressors, air pollution has emerged as one of the most frequently investigated factors associated with adverse neurological outcomes. Recent research emphasizes the complex nexus between exposure to airborne pollutants—such as fine particulate matter (PM_2.5_), nitrogen dioxide (NO_2_)—and compromised brain health across different life stages, from the prenatal period to adult aging [6]. This environmental impact on the central nervous system involves a multifactorial pathogenesis, including increased oxidative stress, systemic inflammation, blood–brain barrier disruption, and epigenetic dysregulation [6,7,8,9]. Specifically, these pollutants have been associated with alterations in gene expression, inflammatory responses, oxidative stress, and structural brain changes, all of which may contribute to adverse mental health outcomes; elucidating these mechanisms is crucial to improve prevention and treatment strategies [10,11,12,13,14].

While air pollution represents a pervasive threat, other environmental stressors consistently pose severe health risks. Heavy metals (from industrial discharges in water, soil, and air) and agrochemicals (pesticides, fertilizers, and herbicides) are strongly associated with the development and progression of mental health disorders and adverse neurological outcomes [8,15,16,17,18,19,20,21,22]. Concurrently, persistent organic contaminants—such as per- and poly-fluoroalkyl substances (PFAS), polychlorinated biphenyls (PCBs), and Polybrominated diphenyl ethers (PBDEs)—pose global environmental health issues. Prolonged exposure to long-chain PFAS (such as PFOA and PFOS), PCBs, and PBDEs are significantly associated with adverse infant neurodevelopment, autism, and metabolic alterations linked to perinatal or antenatal depression [23,24,25,26,27,28].

Given that controlled experimental human exposure is unethical, observational designs are the most appropriate frameworks to assess exposure-related risks [29]. These designs rely on human biomonitoring to track exposure timelines via acute or chronic biological matrices [30,31]. Identifying notable, objectively measurable biomarkers is a major focus; these objectively measurable indicators describe normal or pathological processes, improving the accuracy of clinical diagnoses under low-to-moderate exposure conditions [28,32,33,34,35]. Recent literature categorizes these impacts of environmental stressors into primary clinical clusters. Alzheimer’s disease (AD) and Parkinson’s disease (PD) are increasingly linked to air pollution, acrolein [36], and formaldehyde [37,38], with DNA damage and protein misfolding identified as key drivers. Similarly, Lead (Pb) exposure correlates with developmental neurotoxicity in infants, evidenced by alterations in effect biomarkers (serum brain-derived neurotrophic factor [BDNF], cortisol), susceptibility markers, and nutrient status [39,40]. Beyond classical genetics, contemporary neurology integrates candidate biomarkers from neurophysiology, and emerging fields like transcriptomics, proteomics, and metabolomics [41,42].

Furthermore, air pollution is heavily tied to autism spectrum disorder (ASD) [43] and severe mental disorders through shared inflammatory pathways [10], while climate stressors increasingly threaten perinatal mental health [44]. The current landscape of secondary research reflects a paradigm shift toward population neuroscience, bridging the gap between environmental data, and cellular models [45,46]. Emerging frameworks connect environmental exposure to systemic dysregulation and clinical outcomes, including pathways related to pesticide exposure and other pollutant classes represented in the predefined search framework [47,48]. Mechanistically, environmental pollutants may contribute to neurodegenerative processes through alterations in neuroendocrine signaling and brain connectivity [49,50]. A primary driver is the overactivation of the innate immune system via the NLRP3 inflammasome and the cGAS-STING pathway [51,52]. Triggered by toxins, these pathways activate TBK1 and IRF3, leading to interferon (IFN) release [53,54]. Simultaneously, NF-κB triggers pro-inflammatory cytokines such as IL-6 and TNF, driving the chronic neuroinflammation that underlies neurodegenerative processes [10,53,54,55]. Furthermore, this transition toward a population neuroscience framework reveals that pollutants contribute to a cumulative “pollutome” targeting the brain’s structural integrity. Within this framework, the disruption of the microbiome and the neuroendocrine system represents a critical, yet under-researched, pathway. To consolidate a rapidly expanding but highly fragmented evidence base, this scoping review evaluates scientific literature with no temporal constraints. By focusing on studies utilizing validated biological markers of neurotoxicity—spanning fluid, molecular, epigenetic, and structural endpoints, this work moves beyond subjective or symptomatic endpoints, enabling a meaningful, reproducible interpretation of the state-of-the-art evidence [29]. This synthesis identifies recurrent patterns of association across heterogeneous populations, clarifying the consistency of current data and outlining methodological recommendations to advance environmental risk prediction. By adopting an interdisciplinary perspective, this review addresses the following objectives:

- Identify the main classes of environmental pollutants investigated in relation to neurobiological biomarkers and neurological outcomes—specifically air pollutants (e.g., particulate matter such as PM_2.5_, PM_10_, fine and ultrafine particles; nitrogen dioxide [NO_2_]; sulfur dioxide [SO_2_]; ozone [O_3_]; carbon monoxide [CO]; BTEX and volatile organic compounds [VOCs] including benzene, toluene, xylene, and formaldehyde; polycyclic aromatic hydrocarbons [PAHs]), chemical contaminants (e.g., PFAS, Persistent Organic Pollutants, pesticides including organochlorines and organophosphates), and heavy metals (e.g., lead, cadmium, mercury, arsenic)across residential, occupational, agricultural, dietary, and hospital-based exposure scenarios, as well as internal biomonitoring assessments, and map their specific associations with neurodevelopmental, psychiatric, and neurodegenerative disorders.
- Evaluate early, subclinical signals of neurobiological alteration to capture prodromal processes that precede clinical diagnosis
- Provide a structured overview of recurring “patterns” of pollutants and biomarkers across the reviewed literature
- Pinpoint critical knowledge gaps and areas of toxicological uncertainty to advocate for a multidisciplinary framework bridging toxicology, public health, and environmental policy.

## 2. Materials and Methods

### 2.1. Study Design and Protocol

This scoping review followed the Joanna Briggs Institute (JBI) methodology for scoping reviews and adheres to the Preferred Reporting Items for Systematic Reviews and Meta-Analyses extension for Scoping Reviews (PRISMA-ScR) guidelines [56]. The Population, Concept, and Context (PCC) framework was used to define eligibility criteria and structure the research questions. Prior to screening, an internal protocol was finalized through iterative consensus meetings among investigators to establish the search strategy and inclusion criteria; however, the protocol was not publicly registered. The PRISMA-ScR checklist and the full search syntax are provided in Supplementary Documents S1 and S2.

### 2.2. Research Question (PECOS)

The research objective was defined according to the Population, Exposure, Comparator, Outcome, and Study Design (PECOS) framework:

- Population (P): Human populations of all ages, including children, adolescents, and adults.
- Exposure (E): Environmental toxicant exposures across residential, occupational, agricultural, dietary, and hospital-based settings, as well as internal biomonitoring (e.g., PM_2.5_, NO_2_, O_3_), VOCs, heavy metals, pesticides, and persistent contaminants (e.g., PFAS, PAHs).
- Comparison (C): Cohorts exposed to lower concentrations of pollutants or reference/control groups.
- Outcome (O): Quantitative alterations in biological markers of neurotoxicity, including fluid-based indicators (e.g., Tau protein, neurofilament light chain [NfL], glial fibrillary acidic protein [GFAP], BDNF), structural/functional parameters, epigenetic modifications (DNA methylation), and cellular integrity markers (telomere length [TL]).
- Study Design (S): Observational and epidemiological research, including longitudinal cohorts, cross-sectional studies, and case–control designs.

### 2.3. Search Strategy and Information Sources

The search was performed on 30 March 2026, across four major biomedical and multidisciplinary databases widely recognized for high-impact medical and environmental health literature: PubMed, Scopus, Web of Science, and Embase. The search strategy utilized complex strings of Boolean operators and keywords tailored to the specific indexing syntax of each database (Supplementary Document S2). The keywords were selected based on an initial non-systematic exploratory literature search and were searched within the titles and abstracts of records. No controlled vocabulary (such as MeSH or Emtree terms) was employed. To ensure maximum inclusivity and sensitivity of the search syntax, no time filters or language restrictions or database-specific filters were applied during the search process, aiming to identify a broad range of relevant historical and contemporary literature. Furthermore, 12 “gold standard” articles—identified a priori through preliminary pilot literature screening as representative benchmark studies covering key exposure-biomarker relationships across our scope—were used to refine the query. The final string was iteratively adjusted until it successfully captured all 12 targets publications. This benchmark validation supported the coverage of key exposure-biomarker relationships within the predefined scope; however, it does not demonstrate exhaustive retrieval of all potentially eligible literature. To ensure full methodological transparency and reproducibility, the detailed database-specific search syntax interfaces, and total number of retrieved results for each individual database are provided in the Supplementary Document S2. To further clarify the implications of the search design, the mandatory population/study-design block and some non-specific terms may have reduced sensitivity for records whose titles or abstracts did not contain the predefined descriptors. Therefore, potentially eligible studies may have been missed despite successful retrieval of all 12 benchmark articles. The findings are consequently interpreted as a structured mapping of the literature retrieved by the predefined strategy rather than as an exhaustive census of all environmental-neurotoxicity studies.

### 2.4. Eligibility Criteria

The eligibility criteria were strictly aligned with the thematic clusters of the systematic search strategy (Table 1). To be included, studies had to provide a quantitative analysis of the association between residential environmental stressors—encompassing residential, occupational, agricultural, dietary, and hospital-based exposures, as well as internal biomonitoring assessments—and specific biological markers of neurotoxicity. We prioritized community-based cohorts and studies on cognitively healthy individuals to capture preclinical or subclinical alterations. Only original, peer-reviewed human observational studies were considered, while non-English articles, animal models and secondary literature were excluded.

To ensure a comprehensive mapping of the existing literature—in alignment with PRISMA-ScR guidelines—multiple publications originating from the same longitudinal cohort were deliberately retained if they investigated distinct environmental pollutants, evaluated different biological matrices, or focused on specific, non-overlapping biological markers of neurotoxicity. Because scoping reviews are exploratory and do not involve quantitative meta-analytical pooling, maintaining these distinct study outputs was essential to capture the full spectrum of pollutant–biomarker interactions without omitting critical toxicological data.

### 2.5. Study Selection and Data Extraction

The study selection process was conducted and reported following the PRISMA-ScR recommendations. To ensure transparency and minimize bias, the entire screening and selection process was managed using Rayyan (Rayyan Systems Inc., Cambridge, MA, USA) [57].

Following the removal of duplicates, two reviewers independently screened the identified records by title and abstract in a blinded process. Potentially relevant studies were subsequently subjected to a full-text review to confirm eligibility based on the predefined criteria. Any discrepancies between reviewers were resolved through consensus or, when necessary, consultation with a third senior researcher.

Data extraction from the 52 included studies was performed by two reviewers (O.C. and G.D.). Prior to extraction, a standardized Excel form was collaboratively developed by the two authors to ensure structured data collection. Each reviewer initially extracted data from a distinct, non-overlapping subset of 26 studies. The extracted variables comprised: study identification details (Title, Journal, First Author, Year, Country), epidemiological characteristics (Study Design, Study Period, Target Population, Sample Size), exposure and outcome parameters (Pollutant/s, Environmental Matrix, Exposure Assessment Method, Biomarker, Biological Matrix), and analytical methodologies (Adjusted Confounders, Statistical Test, Main Results).

Following initial extraction, the complete dataset underlying the summary tables and exploratory co-reporting analysis was fully cross-verified by the other reviewer against the original publications as a quality-control step. Any discrepancies identified during verification were resolved by consensus, and missing study-level information was not imputed. The direction and statistical significance of associations were coded qualitatively and quantitatively in the summary table (Supplementary Table S1), reporting odds ratios (ORs), regression coefficients (β), 95% confidence intervals (CIs), and exact *p*-values whenever available. Given the broad scope of this scoping review to map the available evidence, together with the substantial heterogeneity in study designs, exposure assessments, and biological matrices, a formal risk of bias assessment was not performed.

### 2.6. Exploratory Co-Reporting Analysis of Biomarkers and Environmental Pollutants

To complement the synthesis, an exploratory co-reporting analysis was performed using information extracted from the included studies. The objective was to provide a structured overview of recurring patterns of pollutants and biomarkers across the reviewed literature. Using study-level information extracted from the full review table (Supplementary Table S1), the analysis aimed to identify recurring biomarker groups and pollutant groups—and examine how pollutant and biomarker terms were co-reported within the same studies. The persistence of the main descriptive co-reporting structure under a more restrictive occurrence threshold was also assessed.

Each study was treated as the unit of analysis. Biomarker and pollutant terms were extracted from the corresponding fields and harmonized to reduce fragmentation due to the spelling variants, abbreviations, synonyms, and alternative naming/nomenclature. Harmonization was performed upstream of VOSviewer version 1.6.20directly in the analysis dataset. The original extracted terms were retained in the review table for traceability, while harmonized terms were used to construct the exploratory co-reporting maps. A complete harmonization dictionary mapping original terminology variants to the corresponding harmonized terms is provided in the Supplementary Table S2.

Network construction and visualization were performed using VOSviewer version 1.6.20. Three separate Scopus-compatible CSV input files were derived from the review dataset, with each row representing one included study. These files were used to generate: (1) a biomarker-only co-reporting map, (2) a pollutant-only co-reporting map, and (3) a combined pollutant–biomarker co-reporting map. The analysis did not use the original author-provided keywords of the included studies. Instead, the harmonized terms extracted from the biomarker and pollutant fields of the review dataset were entered into a column labeled “Author Keywords”, as required by VOSviewer to process the selected field in a Scopus-compatible input file. For the biomarker-only and pollutant-only analyses, the corresponding harmonized field was assigned this column label. For the combined analysis, the “Author Keywords” field contained both types of terms, prefixed with “Bio_” and “Exp_” to distinguish biomarkers from pollutants, respectively. Thus, “author keywords” referred solely to the technical designation of the VOSviewer input field and not to the provenance of the analyzed terms.

Co-occurrence analysis was performed using the full counting method, with each study treated as the unit of analysis. Link strengths were normalized using the association-strength method, and clusters were identified using the VOS clustering technique with a resolution parameter of 1.00. A minimum occurrence threshold of two studies was used for the primary analysis, and a more restrictive threshold of three studies was applied as a sensitivity analysis. All components satisfying the applicable minimum occurrence threshold were retained; the analysis was not restricted to the largest connected component.

Links between terms indicate that two terms were reported within the same study. Accordingly, the resulting maps represent study-level co-reporting patterns and should not be interpreted as evidence of causal, statistical, or mechanistic associations.

## 3. Results

### 3.1. Study Selection

The initial systematic literature searches across the four selected databases yielded a total of 5935 records. The baseline distribution across the electronic databases was as follows: Scopus (*n* = 2378), Embase (*n* = 1792), PubMed (*n* = 1004), and Web of Science (*n* = 761). Following the identification phase, 2355 duplicate records were removed. A total of 3580 unique records were subsequently retained for title and abstract screening. During this primary screening stage, 3475 records were excluded as they did not meet the predefined inclusion criteria.

**Figure 1:**
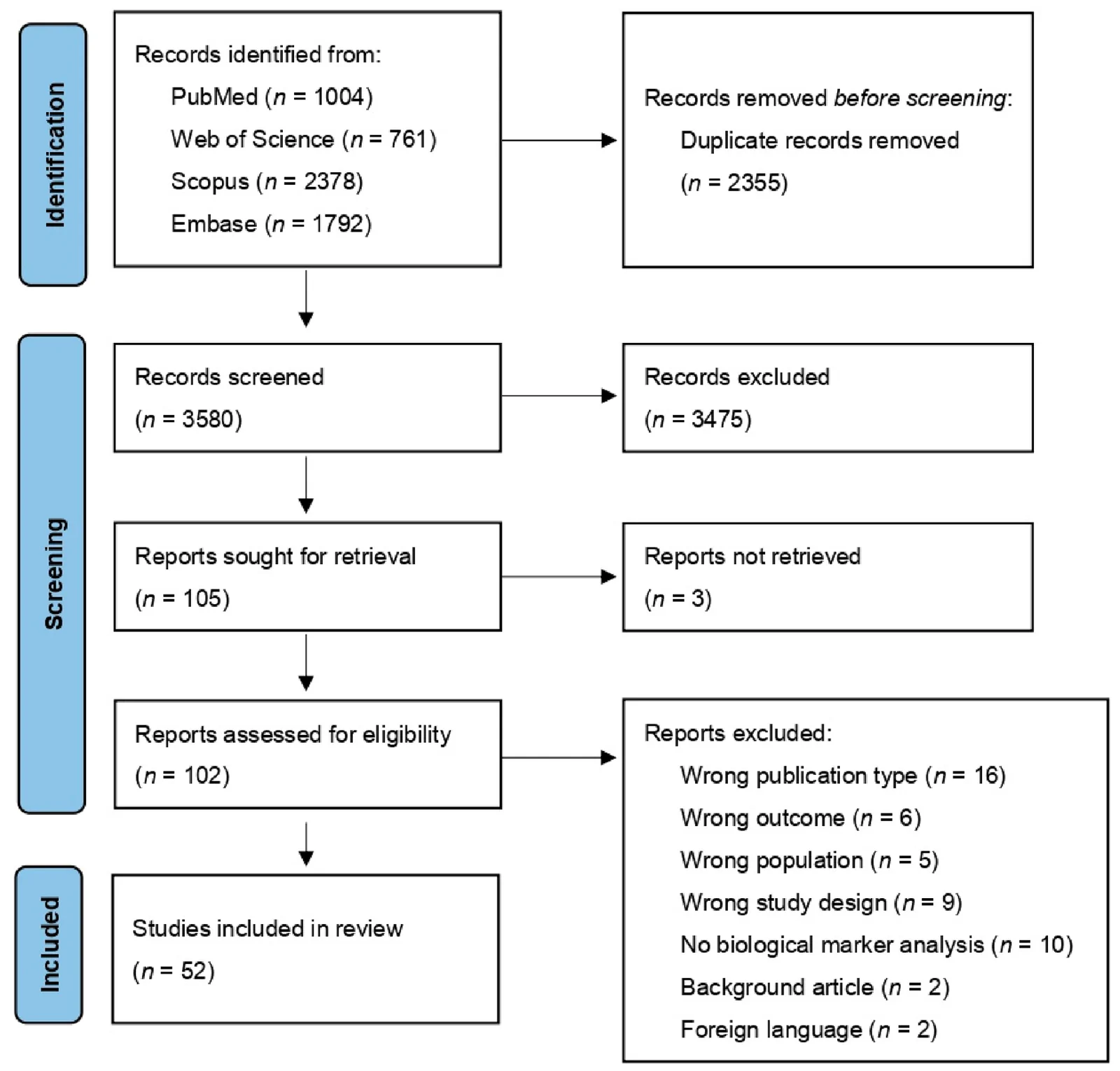
PRISMA-ScR Flow Diagram. Flow diagram of the study selection process following PRISMA-ScR guidelines. Numbers indicate records identified and screened, full-text reports assessed for eligibility and excluded (with primary reasons), and final studies included in the qualitative synthesis.

Consequently, 105 reports were sought for retrieval, of which 3 reports could not be successfully retrieved. The remaining 102 reports were assessed for eligibility through full-text screening. Of these, 50 reports were excluded upon detailed evaluation for the following specific reasons: 16 were the wrong publication type, 10 lacked biological marker analysis, 9 utilized an inappropriate study design, 6 investigated an incorrect clinical outcome, 5 involved an inappropriate target population, 2 were background or non-original articles, and 2 were excluded due to foreign language restrictions. Ultimately, a final sample of 52 studies fulfilled all eligibility criteria and was included in the scoping review (Supplementary Table S1). The complete study selection process is illustrated in the Flow Diagram (Figure 1).

### 3.2. Geographical and Timeline Distribution of the Studies Included in the Review

**Figure 2:**
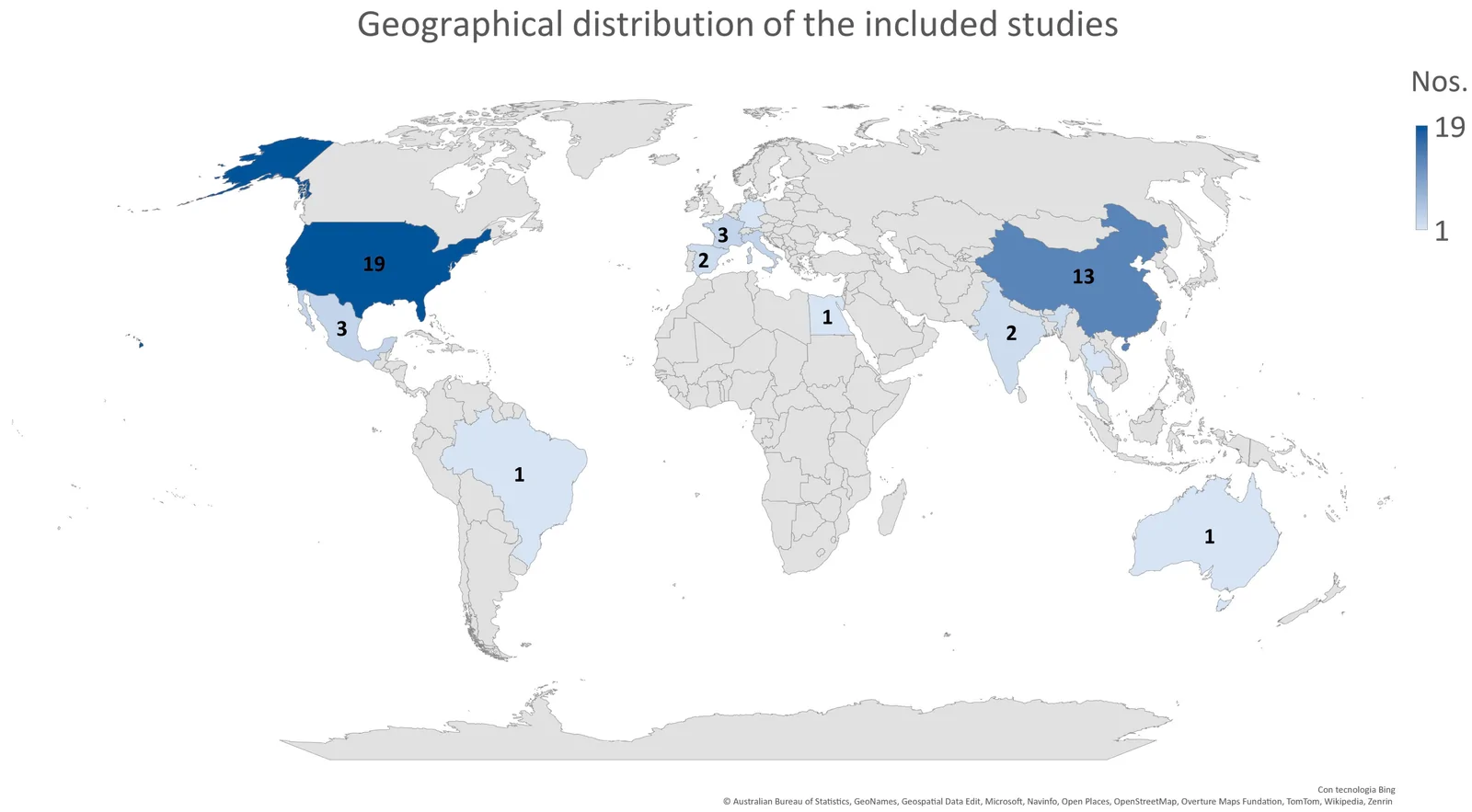
Geographical distribution of the included studies. Caption: Global map showing the country-level distribution of the 52 eligible studies included in the review. Color coding and legend values indicate the absolute number of published studies per country, highlighting a higher concentration of research in North America, China, and Western Europe.

A total of 52 studies, published between 2008 and 2026, were included in this review. The geographical area where the highest number of studies meeting the criteria of interest was conducted is the United States (19 studies), followed by China (13 studies). This distribution may reflect differences in research capacity, environmental monitoring systems, funding availability, and publication output rather than the true global burden of environmentally related neurological disorders. Consequently, the research landscape is predominantly driven by a bipolar axis, with China and the United States leading in total volume and recurrent output. However, the scope of research remains international, with notable contributions from across Europe (including Italy, Spain, France, Germany, and the Czech Republic) and diverse representation from regions such as Mexico, Brazil, Thailand, India, and South Korea. This distribution suggests that while the academic discourse is largely driven by Chinese and American research, the field benefits from a geographically diverse perspective that underscores its global relevance and multidisciplinary nature (Figure 2).

**Figure 3:**
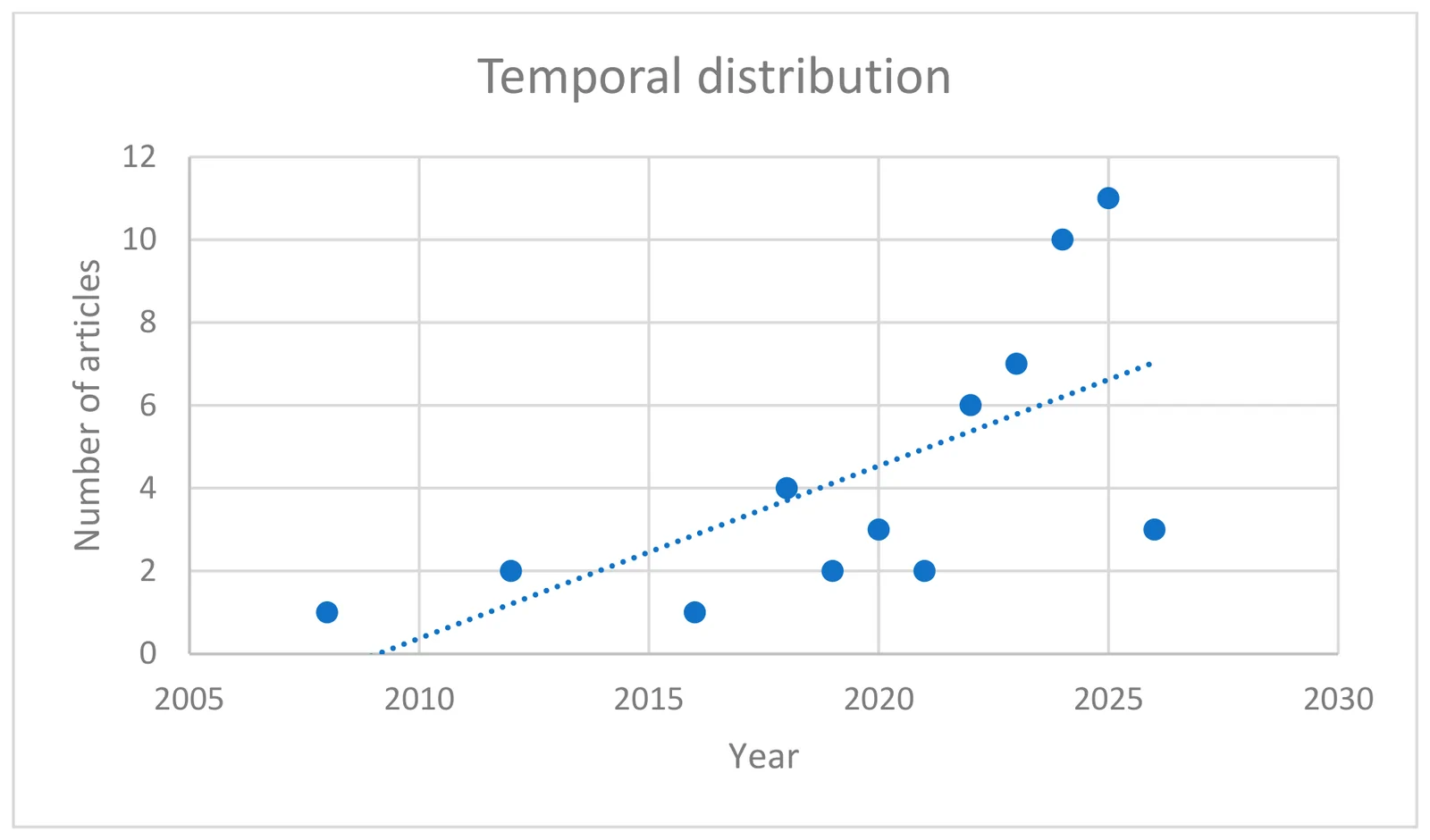
Temporal distribution of the included studies (2008–2026). Caption: Annual publication trend of the 52 included studies from 2008 to 2026. Blue dots represent the absolute number of articles published per year, and the dotted line indicates the overall linear upward trend in research output over time.

Observational epidemiological studies rigorously investigating exposures to environmental pollutants, alongside their relative biomarkers of exposure and outcomes relating to neurological and mental diseases, have developed only recently. Indeed, it can be observed that no studies were conducted prior to 2008, and that scientific interest in this area began to increase particularly from the 2012–2016 period onwards. This surge in interest within the field of neuroepidemiology then became especially pronounced between 2020 and 2025. The 52 studies reflect a rapidly intensifying global interest in this research field, characterized by a significant surge in output over the last three years (2023–2026). From a temporal perspective, there is an exponential growth trend; nearly 80% of the works have been published within the last four years, highlighting an increasingly urgent scientific focus. While the timeline begins in 2008 with sporadic contributions, it transitioned into a period of steady growth, ultimately reaching its peak intensity in 2024 and 2025 This trend reflects a substantial increase in scientific interest and research activity in the field over the last decade (Figure 3).

### 3.3. Overview of Study Designs, Exposures, and Biomarkers in the Included Studies

The included studies demonstrate a growing integration of environmental epidemiology, biomarker research, and molecular approaches aimed at elucidating the biological mechanisms linking environmental exposures to neurodegenerative and neurodevelopmental outcomes, with 52 studies published between 2008 and 2026 included in this review [58,59,60,61,62,63,64,65,66,67,68,69,70,71,72,73,74,75,76,77,78,79,80,81,82,83,84,85,86,87,88,89,90,91,92,93,94,95,96,97,98,99,100,101,102,103,104,105,106,107,108,109].

Sample sizes varied substantially, ranging from small-scale investigations to large cohorts exceeding 31,000 individuals, encompassing cognitively healthy adults, patients with mild cognitive impairment (MCI), AD, PD, and other neurological disorders, as well as occupationally exposed workers, pregnant women, mother-child pairs, children, and adolescents [63,64,66,73,74,76,81,83,86,95,102,104,108].

Cross-sectional study designs represent the predominant methodology in recent research [62,64,70,81,83,101,104,108], although prospective cohorts, birth cohorts, panel studies, longitudinal investigations, and case–control studies remain essential for evaluating temporal dynamics and exposure-outcome relationships [66,71,73,74,75,76,95].

The environmental exposures evaluated are highly heterogeneous, with ambient air pollutants constituting the most frequently investigated category, including PM_2.5_, PM_10_, O_3_, NO_2_, sulfur dioxide (SO_2_), and black carbon [62,63,67,71,83,108]. Other studies focus on heavy metals and trace elements such as lead, cadmium, mercury, manganese, copper, and complex mixtures [81,93,102,104,105], while additional investigations address pesticides, herbicides, VOCs, PAHs, PCBs, per- and PFAS, phthalates, and other environmental toxicants [59,65,66,72,73,74,76,90,91].

Exposure assessment methodologies vary considerably across studies. Air pollution investigations primarily rely on satellite-derived estimates, land-use regression models, spatiotemporal modeling, environmental monitoring networks, and GIS-based residential geocoding [60,70,74,75,83,108]. In contrast, studies on metals and organic contaminants frequently employ biomonitoring approaches quantifying internal exposure levels in blood, urine, serum, CSF, placental tissue, cord blood, and hair using advanced analytical techniques such as Inductively Coupled Plasma Mass Spectrometry, Gas Chromatography-Mass Spectrometry, and Liquid Chromatography-Mass Spectrometry [64,72,76,81,90,102,103,104].

A broad spectrum of neurodegenerative and neuroinflammatory biomarkers was evaluated, including core AD biomarkers such as amyloid-β peptides (Aβ40, Aβ42, and their ratios), total tau (t-tau), phosphorylated tau (p-tau/p-tau181), and soluble triggering receptor expressed on myeloid cells 2 (sTREM2) [80,81,86,108]. NfL emerged as one of the most recurrently assessed biomarkers across serum, plasma, blood, and CSF [63,65,95,99,102,105,109], alongside additional markers including GFAP, α-synuclein, BDNF, TAR DNA-binding protein 43 (TDP-43), ubiquitin carboxy-terminal hydrolase L1 (UCHL1), inflammatory markers, and oxidative stress indicators [62,64,69,107].

Finally, molecular and genetic mechanisms represent a major research focus, with studies investigating DNA methylation and specific gene polymorphisms such as SNCA as potential mediators of environmental neurotoxicity [64,66,67,71,73,74,76,82,83,88,96,103], highlighting the central role of genetic susceptibility and epigenetic regulation in biological responses to environmental exposures and overall demonstrating increasing methodological integration across environmental epidemiology, biomarker research, and molecular omics approaches to elucidate pathways linking environmental exposures to neurodegenerative processes and early neuropathological alterations.

To synthesize the clinical and mechanistic evidence gathered in this review, Table 2 provides an overview of the interaction between environmental toxicants and neurological health. Specifically, it integrates the biological interpretation of key neural damage biomarkers, their most frequently associated pollutant classes, and their predominant pathophysiological effects.

### 3.4. Impact of Ambient Air Pollution on Neurobiological and Neurodegenerative Biomarkers

The impact of ambient air pollution on neurobiological biomarkers and neurodegenerative diseases represents a research field of growing clinical and epidemiological relevance, see Supplementary Document S3. Chronic exposure to fine particulate matter (PM_2.5_) has been associated with lower CSF concentrations of sTREM2, potentially reflecting impaired microglial activity and altered amyloid-beta (Aβ) and tau homeostasis [80,108]. These observed associations may reflect biological pathways through which PM_2.5_ exposure contributes to neurodegenerative processes also on an epigenetic level, determining variations in the DNA methylation of key genes for neuroinflammatory processes such as NFATC1 [85]. Longitudinal studies have reported positive associations between long-term PM_2.5_ exposures and increased central amyloid burden, detectable in the CSF [86]. This trend is also mirrored at the systemic level, where chronic exposure to PM_2.5_, PM10, and nitrogen dioxide (NO_2_) shows clear positive associations with elevated plasma concentrations of Aβ [75,78], while elevated levels of PM10 correlate with greater overall positivity on the same amyloid (Positron emission tomography) PET scans [79]. Furthermore, the definition of precise exposure time windows reveals that the 1-year and 3-year cumulative load of ambient PM_2.5_ is inversely proportional to the CSF concentration of Aβ42, confirming an advanced sequestration of the protein at the cortical level [70]. However, important pollutant-specific null associations have been reported: while PM_2.5_ consistently impacts sTREM2 and amyloid load, gaseous pollutants such as O_3_ and NO_2_ show no significant associations with CSF sTREM2 levels [80,108] or CSF α-synuclein [101], and ground-level O_3_ shows no association with brain amyloid PET scan positivity [78]. Additionally, while 5-year mean exposure to PM10 correlates with an increased risk of amyloid-β positivity on PET scans, it displays no significant associations with tau accumulation, AD-signature cortical thickness, or white matter hyperintensity (WMH) volume [79].

Structural and inflammatory consequences related to air pollution manifest severely within diverse populations and urban ecosystems. In residents of metropolitan areas characterized by high rates of PM_2.5_ and ultrafine particles, a significant increase in TDP-43 protein is observed in the cerebrospinal fluid (CSF), accompanied by a notable systemic pro-inflammatory profile characterized by elevated levels of cytokines and chemokines such as tumor necrosis factor-alpha and MCP-1 [69]. Even within pediatric populations residing in high-pollution areas, children presenting with white matter hyperintensities on magnetic resonance imaging manifest compensatory alterations in immunoregulatory and tissue remodeling profiles to counteract the continuous environmental insult [68]. Contexts of severe environmental stress and extreme occupational exposure, such as those recorded among World Trade Center first responders exposed to toxic dust and fumes, have been associated with biological alterations recurrent with neuroinflammatory and neurodegenerative processes, including reduced plasma levels of Aβ40, a marked reduction in cortical thickness on brain imaging, and an increase in biomarkers of axonal damage [64]. In individuals already diagnosed with MCI, the application of non-linear regression models demonstrates that exposure to vehicle traffic-derived PM10 significantly accelerates the increase in p-tau and the reduction in Aβ1-42 in the CSF, acting as a predictive factor for clinical conversion toward dementia [100]. Conversely, traffic-related PM_2.5_ in other cohorts showed non-significant trends and predominantly null associations with CSF tTau, pTau, and their respective ratios [70]. Regarding synucleinopathies, exposure to PM_2.5_ shows a negative linear correlation with CSF levels of α--synuclein, suggesting a potential blockage of clearance mechanisms or a central sequestration of the protein [101].

The use of high-sensitivity peripheral biomarkers offers a dynamic perspective on neurotoxicity induced by specific chemical and atmospheric pollutants. Serum levels of NfL, a crucial indicator of axonal damage, undergo significant increases in response to exposure to VOCs such as 2,5-dimethylfuran, ethyl acetate, and xylene [65]. Acute spikes and short-term variations in O_3_ exposure are shown to cause a rapid serum increase in both NfL levels and BDNF [94], while the study of particulate metal components highlights that elements such as nickel (Ni), manganese (Mn), and lead (Pb) present strong positive associations with circulating serum concentrations of NfL [98]. These biological findings are solidly supported by comparative field studies conducted in high industrial and environmental impact areas in India, where exposed populations show a significant increase in plasma levels of GFAP and Aβ1-42, accompanied by a noticeable reduction in circulating levels of α--synuclein, total tau (t-tau), and BDNF itself compared to healthy controls residing in unpolluted areas [62,63]. These findings highlight contrasting dynamics between short-term/acute exposure spikes (which transiently elevate serum BDNF and NfL [94,98] and chronic, long-term environmental exposure, which instead correlates with a significant reduction/depletion of plasma BDNF, total tau, and α-synuclein [63].

The molecular interaction between airborne pollutants and neurobiological damage is further modulated by complex epigenetic rearrangements, vascular responses, and host genetic susceptibility profiles. Prenatal exposure to sulfur dioxide (SO_2_) during gestation is associated with alterations in DNA methylation at the imprinted INS-IGF2 locus, an epigenetic modification that mediates the persistence and severity of attention-deficit/hyperactivity disorder (ADHD) symptoms in children during development [71]. However, postnatal SO_2_ exposure shows no significant association with changes in DNA methylation, underscoring the critical sensitivity of prenatal developmental windows [71]. In adults, chronic inhalation of pollutants in high-risk occupational settings promotes specific genome-wide DNA methylation patterns, as evidenced in the biological profiles of police officers [77], while prolonged exposure to O_3_ induces widespread hypomethylation phenomena in genes involved in immune responses and systemic inflammatory diseases [67]. Nevertheless, epigenetic stability varies across time frames and settings: short-term (1-month) ozone-induced DNA methylation changes fail to persist over time compared to intermediate and long-term exposures [67], and no season-specific DNA methylation patterns have been identified in occupational cohorts [77]. Individual vulnerability to these insults is strictly conditioned by the genetic background: the presence of the APOE ε4 allele significantly amplifies susceptibility to cortical Aβ deposition and the subsequent increase in NfL levels induced by air pollution [60]. This biological scenario also interacts with psychological and behavioral factors, where a high polygenic risk score for depression exacerbates the negative impact of PM_2.5_ on functional brain connectivity through the hypermethylation of the SLC30A3 gene [107]. Conversely, protective lifestyles such as high sleep quality can partially mitigate the deleterious neuropsychological effects associated with black carbon exposure [83]. Finally, even in cognitively intact individuals, exposure to PM_2.5_ directly correlates with the development of inflammatory processes affecting the vascular endothelium of the central nervous system, leading to a significant increase in CSF levels of cell adhesion molecules such as Vascular cell adhesion molecule-1 (VCAM-1) and E-selectin [97]— an association that, notably, was not observed or remained inconclusive among participants already diagnosed with MCI or AD [97].

### 3.5. Impact of Heavy Metal Exposure on Neurobiological and Neurodegenerative Biomarkers

The relationship between heavy metal exposure and neurobiological biomarkers has been increasingly investigated in recent years through diverse biomarkers of neurodegeneration, axonal damage, and epigenetic modification, see Supplementary Document S4. Exposure to heavy metals such as cadmium (Cd) and lead (Pb) is recurrently associated with pathological alterations in AD biomarkers, correlating with reduced Aβ42, altered Aβ42/40 ratios, and increased p-tau, mechanisms that are partially mediated by oxidative stress [93,99]. Long-term developmental impacts are profound, with high prenatal lead exposure linked to reduced plasma Aβ42 and altered AD-related gene expression, such as ADAM9 and RTN4, in young adulthood [89], whereas adequate midlife blood zinc (Zn) appears neuroprotective against amyloidogenesis compared to elevated copper-to-zinc ratios [102]. Axonal injury, captured by NfL, is highly sensitive to metal toxicity; Cd and Pb were the metals most recurrently associated with elevated serum NfL concentrations in general adult populations [84,104,109], with NfL actively mediating the association between Cd and cognitive decline [109]. This NfL elevation is similarly profound in clinically diagnosed AD patients exposed to toxic metals and pesticides [95]. Occupational studies reveal further mechanistic pathways: a nickel (Ni) and cobalt (Co)-driven metal mixture significantly elevates plasma sTREM2, supporting a potential role of sTREM2-mediated neuroinflammatory pathways in the association between metal exposure and cognitive impairment [81], while welding fume exposure alters NfL and sphingosine-1-phosphate (S1P) via Ni, arsenic (As), and Pb, with Zn again displaying an inverse, neuroprotective association [105]. Finally, heavy metals disrupt fundamental neurotrophic and epigenetic regulation. High maternal arsenic exposure drastically increases the risk of depleted plasma BDNF [106], and dietary mercury (Hg) exposure correlates with overexpressed S100B mRNA, serving as a peripheral indicator of neurotoxicity [61]. Epigenetic vulnerability is further underscored by findings that early-life lead exposure induces persistent, sex-specific DNA methylation differences in imprinted genes [82], while childhood blood mercury interacts with DNA methylation at GRIN2B and BDNF loci to influence sustained attention and ADHD-related metrics [73].

Alongside these marked neurotoxic effects, several pollutant-specific null, non-linear, or protective associations have been reported, emphasizing the complex biological behavior of specific element fractions and biological matrices. While high maternal blood arsenic concentration is associated with a higher likelihood of low plasma BDNF levels, maternal exposures to lead (Pb), mercury (Hg), or cadmium (Cd) showed no significant associations with BDNF levels [106]. Furthermore, in multi-element mixture studies using weighted quantile sum (WQS) models on general adult populations, urinary barium (Ba) and thallium (Tl) emerged as the primary negative contributors to serum NfL levels [104], while elements like manganese (Mn) and selenium (Se) consistently displayed negative weights, counteracting the axonal damage and elevated sNfL driven by cadmium and lead [109]. Complex, non-linear patterns are also evident in occupational settings: among welders, exposure to urinary nickel (Ni) exhibits an inverted U-shaped relationship with serum NfL, S1P, and dopamine (DA), rather than a monotonic toxic response [105]. In populations evaluating trace element homeostasis, trace metals such as copper (Cu), iron (Fe), manganese (Mn), and zinc (Zn) displayed highly heterogeneous, non-linear, and largely null associations across CSF and serum matrices for core AD biomarkers, whereas serum cadmium showed a clear monotonic association [99]. Finally, in environmental mercury exposure studies among riverine populations, despite a significant upregulation of peripheral S100B mRNA expression among highly exposed individuals, no direct quantitative correlation was found between total hair mercury levels and self-reported clinical neurological symptoms [61].

### 3.6. Impact of Pesticide Exposure on Neurobiological and Neurodegenerative Biomarkers

The relationship between pesticide exposure and neurobiological integrity is extensively documented through epigenetic alterations, DNA methylation shifts, and molecular biomarkers of neurotoxicity, see Supplementary Document S5. Long-term ambient exposure to copper pesticides has been associated with differential DNA methylation patterns in peripheral blood, targeting the COL11A2 gene and pathways enriched for Fc gamma R-mediated phagocytosis, homophilic cell adhesion, and EGF receptor signaling, which have previously been implicated in neuroinflammatory and neurodegenerative processes in PD pathology [74]. Similarly, chronic ambient organophosphate exposure correlates with differential DNA methylation across both blood and saliva matrices at multiple CpG sites, showing notable enrichment for nicotinic and muscarinic acetylcholine receptor signaling pathways-the direct biological targets of organophosphate toxicity; notably, global mean methylation levels in PD patients decrease progressively with increasing organophosphate exposure [91]. This epigenetic vulnerability exhibits pronounced sex-specific profiles, with occupational and household pesticide exposure revealing 69 differentially methylated regions associated with early-stage PD in females compared to only two in males, indicating that cis-genetic variation explains interindividual DNA methylation variance more significantly than pesticide exposure alone [96]. Beyond Parkinsonian pathology, toxic epigenetic effects extend to neurodevelopmental risks, where elevated exposure to the organochlorine DDE significantly associates with genome-wide differentially methylated regions in sperm, targeting genes significantly enriched for neurological functions and neurodevelopmental processes that substantially overlap with known ASD risk genes [87]. Regarding classical genetic susceptibility, general pesticide and specific herbicide exposures increase PD risk predominantly in younger subjects (age 60 years), operating through independent effects alongside SNCA REP1 base pair length polymorphisms [66] without significant pairwise gene-environment interactions. Finally, molecular neurotoxic impacts are identifiable from early life, as prenatal organophosphate exposure significantly elevates neonatal neurotoxicity biomarkers in umbilical cord blood according to distinct sex-specific profiles; maternal pesticide metabolites link to elevated cord GFAP primarily in males, whereas flame retardant metabolites correlate with increased GFAP and UCHL1 predominantly in females [76]. Conversely, null associations were also observed in this matrix, as prenatal exposures to organophosphates showed no overall significant association with umbilical cord blood S100B levels across the cohort [76].

### 3.7. Impact of Diverse Environmental Pollutants on Neurodegenerative Biomarkers, Epigenetic Reprogramming, and Neurodevelopment

Internal exposure to diverse environmental pollutants significantly impacts central nervous system integrity, altering core CSF biomarkers, epigenetic structures, and autoimmune profiles, see Supplementary Document S6. For instance, PFAS—specifically PFHxS, PFOA, and PFOS—recurrently infiltrate the CSF of older adults, with significantly elevated concentrations of total, branched, and individual congeners found in patients presenting combined cognitive impairment and a pathological Aβ (Aβ1-42/40) ratio [72]. Similarly, non-targeted metabolomic screening underscores chemical specificities in neurodegenerative differentiation, demonstrating a significant 2.7-fold higher concentration (escalating to 3.8-fold in the demented subgroup) of the phthalate bis(2-ethylhexyl) phthalate (DEHP) in the CSF of patients with Dementia with Lewy Bodies compared to AD controls [59]. Conversely, targeted screening within the same matrix revealed no significant differences in CSF pesticide concentrations between Dementia with Lewy Bodies patients and AD controls [59].

Beyond direct fluid biomarkers, maternal and prenatal toxicant exposures profoundly reshape neurodevelopment via notable epigenetic modifications. Maternal serum PCBs significantly correlate with altered placental co-methylation modules that map to key ASD risk genes, specifically CSMD1 and AUTS2, which is associated with lower child neurodevelopmental scores [90]. However, no direct population-level association was observed between maternal serum PCB levels and categorical child ASD diagnosis [90]. This prenatal vulnerability is further highlighted by low-dose exposure to PCBs and polychlorinated dibenzo-p-dioxins and dibenzofurans (PCDD/Fs), which induces distinct sex-stratified alterations in cord blood DNA methylation across 32 positions and 8 regions crucial for neurodevelopment, gene regulation, and immune functioning [88]. Furthermore, airborne PAHs exert gestational neurotoxicity through gene-environment interactions and chromosomal alterations; prenatal PAH exposure interacts with core cord blood DNA methylation profiles across multiple genes (including CYP2E1 and LOC84931) to predict ADHD and mental development index deficits at age three [103]. Concurrently, prenatal PAH exposure quantified via molecular PAH-DNA cord adducts correlates inversely with newborn relative TL, marking early cellular vulnerability although TL does not directly mediate downstream developmental outcomes [92]. Finally, chronic exposure to mixed toxicants, including contaminated drinking water, indoor mold, and assorted chemicals, drives systemic neurotoxic responses characterized by extensive autoantibody generation; exposed individuals manifest profoundly elevated serum levels of neural autoantibodies targeting Tau, microtubule-associated protein 2, neurofilament protein, myelin basic protein, GFAP, and tubulin, with specific autoantibody profiles tightly correlating with clinical symptoms like neuropathy, chemical sensitivity, sleep apnea, or musculoskeletal pain, whereas baseline control markers like S-100B did not show overall elevated autoantibody profiles [58].

### 3.8. Structured Overview of Recurring Patterns of Pollutants and Biomarkers Across the Reviewed Literature

The included studies were highly heterogeneous in terms of exposure assessment, biomarker selection, biological matrices, study populations, and analytical approaches. This heterogeneity provided an opportunity to explore how environmental pollutants and biomarkers are distributed and co-reported across the selected studies. An exploratory co-reporting analysis was conducted using information extracted from the 52 included studies. The graphs, tables and a full description of the logical steps followed in this analysis are set out in the Supplementary Materials (Supplementary Document S7).

In the network maps each node represents a biomarker or an environmental pollutant; the size of a node is proportional to the number of studies in which the term appears; a link between two nodes indicates that the two terms were reported within the same study; the strength of a link increases with the number of studies in which the two terms were reported together; the distance between nodes reflects how frequently the terms are co-reported in the literature, with closer nodes indicating stronger co-reporting patterns. Colors identify groups of terms that tend to appear together more frequently.

The biomarker-only map showed a structured pattern of co-reporting among neurodegeneration- and neuroinflammation-related biomarkers. At the primary threshold, 21 biomarker terms were retained and grouped into four clusters. The most connected terms included Aβ42, total tau, p-tau, Aβ40, NfL and DNA methylation (Supplementary Document S7, Figure S1). The pollutant-only map revealed overall co-reporting patterns across all components (Supplementary Document S7, Figure S2), with specific sub-networks identifying metal exposures centered on cadmium (Supplementary Document S7, Figure S3) and air pollutants focused on PM_2.5_ (Supplementary Document S7, Figure S4).

The combined pollutant-biomarker map was considered the most informative output, as it allowed exposure and biomarker terms to be visualized within the same co-reporting structure. At the primary threshold (2 occurrences), the map retained 50 items and four clusters. The most frequent and highly connected terms included Aβ42, p-tau, total tau, NfL, PM_2.5_, cadmium, and lead. Based on the dominant cluster composition, the combined map suggested recurring study-level domains involving air pollution and amyloid/tau-related biomarkers, metal and trace-element exposures, neural injury/glial-neurotrophic biomarkers, and epigenetic biomarkers (Supplementary Document S7, Figures S5 and S6).

**Figure 4:**
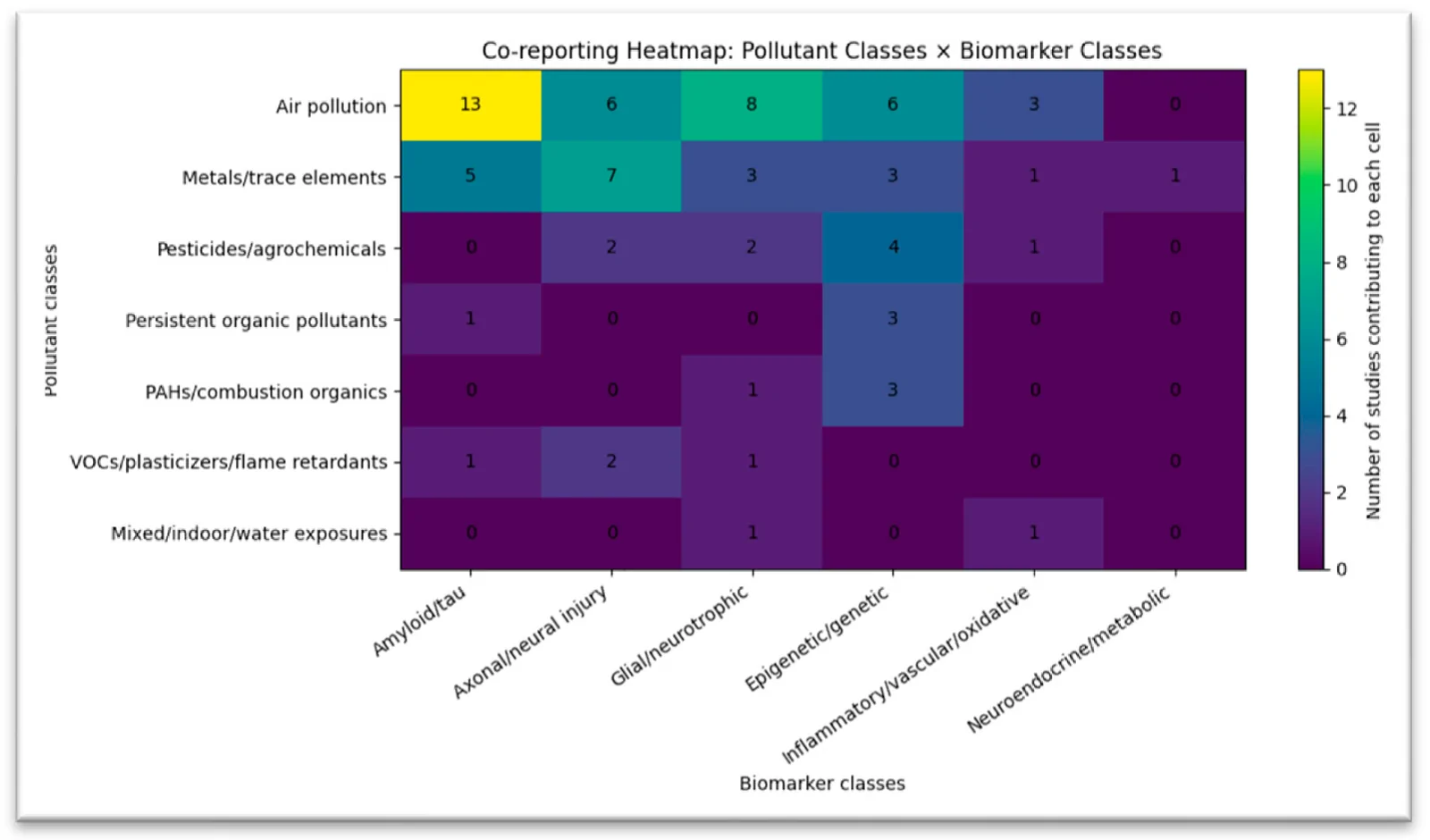
Heatmap of pollutant classes by biomarker classes. Co-reporting matrix displaying study-level publication counts across major pollutant categories (y-axis) and biomarker domains (x-axis). Cell numbers and color scale indicate the absolute number of included studies reporting associations for each specific pair, highlighting air pollution and heavy metals as the most frequently co-analyzed exposure classes.

To enhance data accessibility, a class-level heatmap was used as an additional readability layer (Figure 4). In this output, rows represent pollutant classes and columns represent biomarker classes; each cell counts the number of studies in which the corresponding pollutant and biomarker classes were co-reported. This figure is useful for communicating the table’s structure to readers who may not need the full term-level network.

In the combined analysis, the map retained four clusters under both thresholds. Terms removed by the more restrictive threshold were mainly low-frequency or peripheral terms, whereas the main co-reporting domains remained visible. This could support the consistency of the descriptive structure identified in the primary analysis.

A possible representation of the Study Traceability Matrix was performed. Each bubble represents one study included in the review (Supplementary Table S1). A study appears in a cell if it reports at least one pollutant from the corresponding pollutant class and at least one biomarker from the corresponding biomarker class. Multiple bubbles within the same cell indicate that several studies contributed to that specific pollutant–biomarker combination (Supplementary Document S7, Figure S7).

## 4. Discussion

### 4.1. Main Interpretation and Integrative Systems Framework

The comprehensive synthesis of the literature achieved in this scoping review reinforces the interpretation that environmental exposures do not act through isolated, independent toxic mechanisms but rather contribute to neurodegenerative processes through highly integrated, cross-talking biological systems [110,111].

Rather than acting as discrete, independent causal agents that produce isolated pathological endpoints, environmental stressors operate within a multi-dimensional biological network. This network continuously interacts with cellular decline, specific genetic susceptibilities (such as APOE status), microvascular structural integrity, and systemic immune system dynamics. This system-level perspective implies that the phenomenon under consideration is best conceptualized as an emergent property of long-term, non-linear interactions between a shifting environmental exposome and host biology. Consequently, the most critical translational implication of this evidence is the necessary abandonment of traditional single-pollutant paradigms in favor of a multi-layered model of host vulnerability, wherein cumulative environmental stress acts synergistically with the host’s biological profile [110,111,112,113]. Furthermore, the emergence of pollutant-specific null associations and non-linear response patterns (such as inverted U-shaped relationships observed for specific urinary metals) highlights that environmental neurotoxicity does not follow a simple dose-dependent model. The lack of uniform associations across all studied pollutants underscores the necessity of avoiding publication bias and oversimplified causal assumptions. Instead, it points toward complex biological threshold effects, compensatory physiological mechanisms, and matrix-dependent toxicokinetics that determine whether a specific exposure translates into detectable neurodegenerative biomarker shifts.

### 4.2. Neuroinflammation as a Convergent Biological Pathway

A central interpretation emerging from the reviewed literature is that neuroinflammation represents a convergent biological pathway linking chemically and structurally heterogeneous environmental exposures to downstream neuronal dysfunction [114,115].

**Figure 5:**
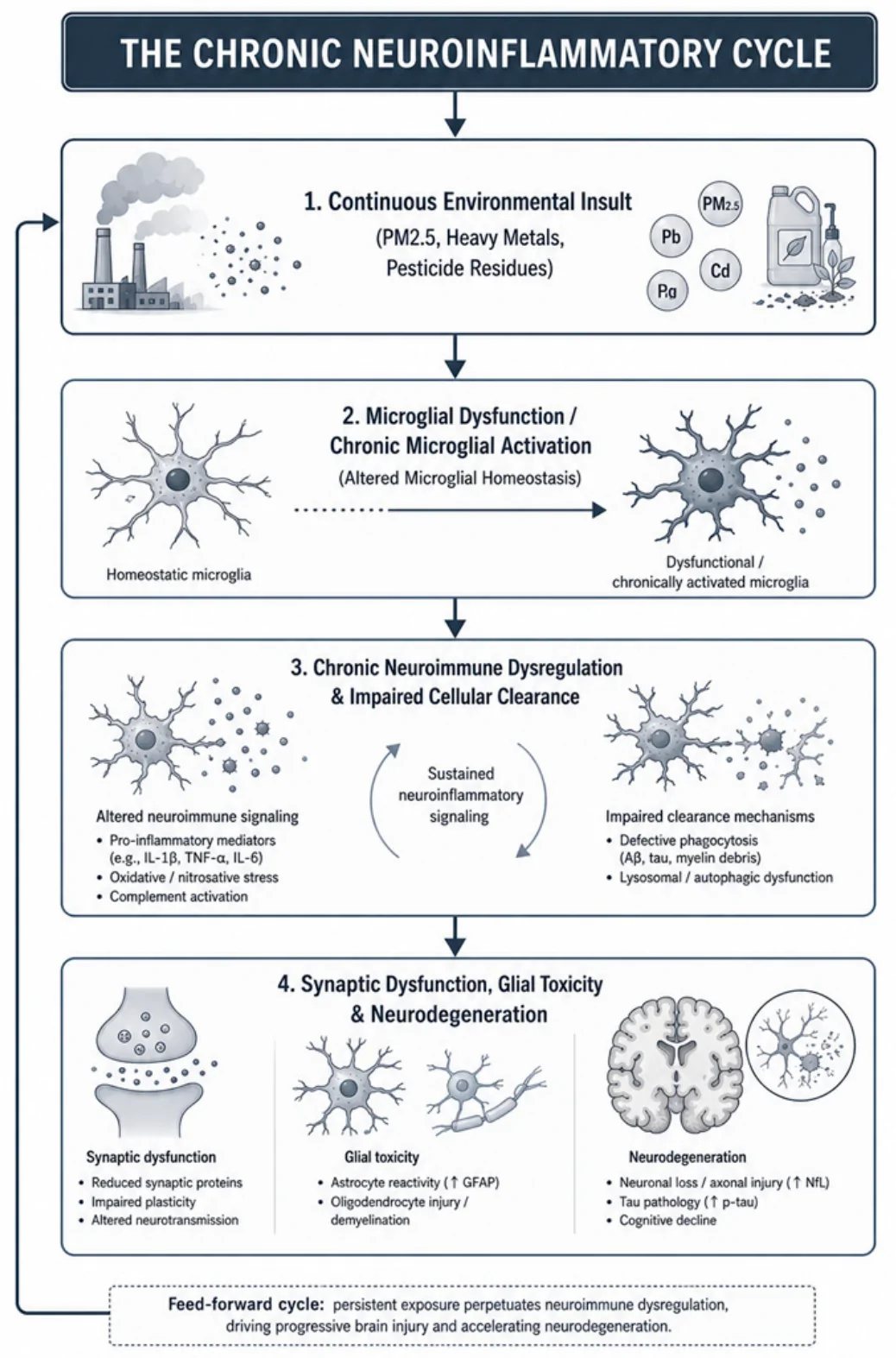
The Chronic Neuroinflammatory Cycle. Schematic representation of the feed-forward mechanism linking exogenous environmental insults (e.g., PM_2.5_, heavy metals, pesticides) to microglial activation, neuroimmune dysregulation, impaired protein clearance, synaptic failure, and progressive neurodegenerative damage. The conceptual workflow, structural layout, and final assembly were designed by the authors using Microsoft PowerPoint 2608 version (Microsoft Corp., Redmond, WA, USA), with visual illustration assets generated with the assistance of AI tools (ChatGPT-4, OpenAI, San Francisco, CA, USA) and subsequently reviewed and verified by the authors.

Across diverse exposure domains—including particulate air pollution, heavy metals, and industrial pesticides—and heterogeneous epidemiological study designs, activation of immune and inflammatory pathways within the central nervous system recurrently emerges as a response to chronic environmental and systemic toxic stress. This convergence supports the concept that neuroinflammation may function as a final common pathway of environmental neurotoxicity, rather than an exposure-specific pathological signature (Figure 5). Within this framework, microglial dysregulation and priming are considered key intermediate processes in the transition from exogenous environmental insult to progressive neurodegenerative pathology [116,117].

Under conditions of sustained environmental stress, microglia shift from a transient, homeostatic, and protective immune phenotype toward a persistently activated state. As illustrated in the proposed neuroinflammatory cycle, this chronic activation is associated with a self-perpetuating release of pro-inflammatory mediators, coupled with a progressive loss of homeostatic functions. The resulting imbalance contributes to impaired synaptic maintenance, reduced neurotrophic support, glial toxicity, and a diminished capacity for clearance of pathological protein aggregates, including amyloid-β and α-synuclein. In this context, emerging evidence also implicates inflammasome pathways, including NLRP3 activation, as potential amplifiers of sustained neuroinflammatory signaling, further reinforcing chronic microglial activation and downstream neurotoxicity. Ultimately, persistent neuroinflammatory signaling is proposed to bridge initial environmental exposures and system-level neurodegenerative processes, including synaptic dysfunction, gliotoxicity, and progressive neuronal loss [118,119,120].

However, a critical unresolved issue concerns the temporal and causal positioning of neuroinflammation within the broader neurodegenerative cascade [116]. Specifically, it remains difficult to determine whether environmental exposures act primarily as initiators of neuroinflammation that subsequently drives neurodegeneration, or whether inflammatory activation represents a secondary response to early, subclinical neurodegenerative processes induced directly by xenobiotic toxicity. Resolving this ambiguity is essential for optimizing the timing and precision of anti-inflammatory and neuroprotective interventions, as well as for refining public health prevention strategies aimed at mitigating environmental risk factors within the preclinical window of disease progression.

This temporal complexity is further demonstrated by contrasting exposure dynamics across acute versus chronic windows. While short-term exposure spikes may trigger transient compensatory surges in neuroprotective or axonal markers (such as serum BDNF and NfLs), prolonged chronic exposure ultimately leads to their exhaustion and depletion. Conversely, the selective absence of associations between gaseous pollutants (e.g., O_3_, NO_2_) and microglial or synuclein markers (such as CSF sTREM2 and α-synuclein) indicates that convergent neuroinflammatory pathways are not uniformly activated by every environmental agent, but are contingent upon specific chemical speciation, particulate properties, and central bioavailability.

### 4.3. Vascular Contributions and Systemic Integration

The evidence supports the view that environmental neurodegeneration is inextricably linked to systemic and cerebral vascular dysfunction [121]. Rather than being anatomically restricted to the enclosed parenchyma of the central nervous system, many of the primary biological alterations associated with environmental pollutants originate within peripheral organ systems—most notably the respiratory tract, the gastrointestinal tract, and the systemic vascular endothelium—and subsequently propagate toward the brain through complex, interconnected systemic pathways. Endothelial activation, localized blood–brain barrier (BBB) disruption, and the subsequent impairment of neurovascular coupling emerge as critical intermediary processes that directly connect peripheral exposomic injuries to central neuronal damage [117,122].

Chronic exposure to circulating ultra-fine particles or heavy metals was associated with systemic oxidative stress and endothelial nitric oxide synthase (eNOS) uncoupling. This leads to increased BBB permeability, which facilitates the unregulated entry of peripheral pro-inflammatory cytokines, circulating environmental toxicants, and immune cells into the brain parenchyma, thereby driving a chronic, self-perpetuating neuroinflammatory state and progressive synaptic degradation. This body of evidence strongly reinforces an increasingly accepted paradigm shift in modern neuroscience, which conceptualizes neurodegenerative disorders not as exclusively brain-centered proteinopathies, but as systemic pathologies with profound central nervous system manifestations [123]. Nevertheless, the exact relative contribution of vascular-mediated pathology versus direct, trans-olfactory or axonal neurotoxic transport mechanisms remains poorly quantified across human cohorts, and it is highly probable that their relative pathological weight varies dynamically depending on the specific chemical properties of the exposure, the duration and route of entry, and distinct host susceptibility profiles.

Notably, the observation that air pollution-induced elevations in vascular inflammatory markers (such as CSF VCAM-1 and e-Selectin) are prominent in cognitively intact adults but remain inconclusive or non-significant in patients with established MCI or AD underscores a critical window of vulnerability. This pattern suggests that vascular endothelial disruption may represent an early driver of neurodegenerative cascades that reaches a saturation ceiling once overt clinical pathology is established, rendering vascular biomarkers less sensitive to environmental insults in late-stage diseases.

### 4.4. Epigenetic Mechanisms and Long-Term Biological Embedding

Epigenetic regulation emerges from this scoping review as a foundational, high-resolution mechanistic framework that explains how transient or chronic environmental exposures are converted into long-term, persistent alterations in neurobiological architecture [124]. Through highly coordinated modifications—including localised DNA methylation shifts, specific histone tail modifications, and altered expression profiles of circulating and central non-coding microRNAs—environmental stressors can induce stable alterations in cell-specific gene expression landscapes.

This process, defined as “biological embedding,” provides a compelling molecular explanation for both the characteristically prolonged latency period of neurodegenerative conditions and the extensive inter-individual clinical variability observed within identically exposed populations. Epigenetic archiving is particularly critical during early-life or prenatal developmental windows, where heightened tissue plasticity and rapid epigenetic programming mean that transient environmental insults can permanently alter the developmental trajectory of neuroimmune and neurovascular systems, thereby creating an elevated baseline susceptibility to neurodegenerative decline later in life [125,126,127,128].

While DNA methylation shifts—induced by factors such as heavy metals and fine particulate matter—represent the most extensively documented mechanism within the reviewed human evidence base, other epigenetic pathways are increasingly recognized. Specifically, chromatin remodeling via histone modifications (often linked to pesticide and microplastic exposure) and non-coding RNA dysregulation (associated with traffic-related air pollution and VOCs) appear as emergent mechanistic pathways. These processes contribute to broader pathological outcomes, including the transcriptional silencing of neuroprotective genes, the sustained expression of pro-inflammatory cytokines, and the post-transcriptional downregulation of synaptic plasticity proteins.

However, a major limitation identified in the current human evidence base is its heavy reliance on cross-sectional or short-term longitudinal Epigenome-Wide Association Studies. This reliance inherently limits our capacity to distinguish between ‘state-dependent’ markers—which merely reflect acute cellular stress—and ‘trait-dependent’ epigenetic changes that function as stable, causal drivers of disease [129]. Consequently, it remains uncertain whether the identified environmental epigenetic marks are functional initiators of the neurodegenerative process or epiphenomenal signatures of cumulative exposure. Furthermore, an essential, unresolved question with profound translational implications concerns the potential reversibility of these modifications [130]. Determining whether such marks can be ‘reset’ through targeted interventions represents a critical frontier for precision prevention strategies.

This mechanistic uncertainty is reinforced by findings on the temporal instability and context-dependency of environmental epigenetic marks. For instance, short-term (1-month) ozone-induced DNA methylation changes fail to persist over time compared to intermediate and long-term exposures, and no season-specific methylation profiles are observed in occupational cohorts. These null and transient findings caution against treating short-term epigenetic fluctuations as permanent disease drivers, highlighting the need to distinguish ephemeral cellular stress responses from stable, lifelong biological embedding.

### 4.5. Heterogeneity, Specificity, and Trans-Diagnostic Pathological Overlap

An important and complex conceptual challenge highlighted by this scoping review is the distinct lack of a clear, one-to-one correspondence between specific classes of environmental exposures and distinct, traditional clinical phenotypes [131]. Instead, highly diverse environmental stressors appear to systematically converge onto overlapping and shared biological pathways. This suggests that environmental factors operate primarily by driving generalized pathogenic mechanisms (such as pan-cortical neuroinflammation or generalized microvascular decay) rather than triggering disease-specific etiologies. This striking biological convergence provides a compelling explanation for the extensive clinical overlap frequently observed across distinct neurological and mental disorders, including shared and co-occurring features of multi-protein aggregation, widespread synaptic drop-out, and chronic glial activation. Concurrently, the eventual manifestation of a specific clinical phenotype is likely shaped by the highly complex, non-linear interactions between an individual’s unique lifetime multi-pollutant exposure profile, structural genetic background, the precise developmental timing of exposures, and the cumulative lifetime target-organ burden. Consequently, these insights support an important paradigm shift away from traditional, strictly nosological and disease-centered diagnostic frameworks toward pathway-based and trans-diagnostic models [131]. However, this conceptual evolution complicates the development of exposure-specific clinical risk classification systems and highly targeted, pollutant-specific public health preventive strategies.

### 4.6. Methodological Heterogeneity, Publication Bias, and the Value of Null Results

A balanced synthesis of environmental neurodegeneration requires equal consideration of negative, null, and non-linear findings to counteract potential publication bias toward statistically significant results. Across the reviewed literature, several high-quality cohorts reported null associations—such as the lack of effect of gaseous pollutants on CSF sTREM2 and α-synuclein, the absence of maternal heavy metal effects (Pb, Hg, Cd) on cord BDNF, or the lack of association between pesticide exposure and CSF S100B levels. Furthermore, multi-element mixture models frequently reveal protective or inverse weights for specific trace elements (e.g., Mn, Se, Ba, Tl) counteracting primary toxic drivers like lead and cadmium.

These inconsistent and null findings should not be interpreted merely as methodological failures, but rather as critical indicators of:

- Tissue- and Matrix-Specific Compartmentalization: Biomonitoring differences across blood, CSF, urine, and target tissues.
- Stage-Dependent Susceptibility: Environmental impacts that vary drastically between healthy baseline status and established neurodegenerative disease.
- Antagonistic Mixture Dynamics: The complex interactive reality of real-world multi-pollutant exposures where essential trace elements buffer toxicant-induced damage.

Systematically documenting null and non-monotonic responses is essential for refining epidemiological risk models, establishing realistic safety thresholds, and ensuring that public health interventions target truly causal environmental determinants.

### 4.7. Limitations and Methodological Considerations

All 52 synthesized studies rely on observational epidemiological designs, which inherently preclude direct inferences of causality. The network maps and co-occurrence figures presented herein reflect reported correlational patterns between exposure classes and fluid biomarkers rather than confirmed cause-and-effect relationships. Given the substantial heterogeneity across exposure assessment methods (e.g., satellite spatial modeling vs. blood biomonitoring), analytical platforms, and cohort demographics, quantitative meta-analysis was neither feasible nor methodologically sound. Consequently, our findings remain restricted to qualitative synthesis and topological mapping. Fluid biomarkers measured in blood or CSF represent surrogate peripheral signals of central neuropathology. While our proposed feed-forward neuroinflammatory loop is grounded in pre-clinical mechanistic evidence, human observational studies offer indirect evidence and cannot currently verify the exact in vivo temporal sequence of parenchymal injury. The current evidence base exhibits a pronounced geographical selection bias that limits global generalizability. Over 60% of eligible studies originated from the United States (*n* = 19) and China (*n* = 13), whereas population-based evidence from low- and middle-income countries (LMICs) across Africa, Southeast Asia, and Latin America remains critically scarce. This discrepancy undermines the capacity to capture real-world exposome interactions under extreme environmental exposures typical of developing regions. Despite the overall consistency and biological plausibility of the documented associations, the current evidence base remains significantly constrained by several major methodological limitations [132]. The heavy predominance of observational and ecological study designs inherently restricts the capacity to infer definitive causality, a challenge that is further compounded by the presence of complex, unmeasured confounding factors, as well as the risk of reverse causality in cross-sectional studies. Furthermore, the high degree of heterogeneity in exposure assessment methods (for instance, spatial modelling versus blood biomonitoring), biomarker selection criteria, and clinical outcome definitions introduces high variability across the literature. This fragmentation limits cross-study comparability. Many historical cohorts rely on indirect, macro-level spatial proxies for both exposure and disease status, which introduces significant non-differential measurement error and typically attenuates or skews observed associations. Finally, the challenge of residual confounding remains a major hurdle in environmental neuroepidemiology [132]. Residual confounding, associated with tightly correlated socioeconomic gradients, localized environmental noise pollution, specific lifestyle factors (such as smoking, diet, and physical inactivity), heterogeneous occupational history, and complex medical comorbidities, can rarely be fully adjusted for in observational datasets. Collectively, these limitations indicate that whilst the current evidence base is suggestive and biologically cohesive, it cannot yet be interpreted as definitively causal. In addition, because the exploratory network analysis uses publications as the analytical unit, multiple reports originating from the same underlying cohort may contribute repeatedly to node frequencies and link strengths. Grounded in the data extracted from the full review table (Supplementary Table S1), it reflects study-level co-reporting patterns rather than causal, statistical, or mechanistic relationships; hence, the findings remain dependent on the quality and consistency of the extracted data. Although terms were harmonized to mitigate fragmentation, the methodological choices made during this process may influence the resulting maps and should be reviewed with caution. Highly connected terms must not be interpreted as stronger biological evidence, but rather as terms more frequently co-reported or co-studied within the included literature.

From a methodological perspective, potential constraints stemming from the initial search strategy must also be acknowledged. The deployment of a mandatory population and study-design conceptual block (incorporating terms such as “adults”, “community”, “cohort”, and “observational study”) may have inadvertently constrained overall search sensitivity, potentially missing eligible studies that omitted these explicit descriptors from their titles or abstracts. Additionally, the inclusion of short or non-specific keyword variants and syntax fragments (e.g., short chemical abbreviations or string combinations) may have introduced indexing noise or suboptimal term alignment during record retrieval. Consequently, low-frequency terms and patterns observed in the overlay maps should be interpreted cautiously, as these observations may be inherently influenced by the limited number of studies analyzed and the specific boundaries of the search strategy.

Finally, a methodological characteristic of this scoping review is the inclusion of several studies originating from the same epidemiological cohorts. In quantitative meta-analyses, such cohort overlap violates the assumption of independence of evidence and can artificially inflate statistical precision. However, because this review is exploratory and does not perform any statistical pooling or meta-analytical synthesis, retaining these studies was deemed essential to achieve a comprehensive mapping of the field. This approach allowed us to capture the full spectrum of distinct environmental insults (ranging from ambient air pollution to heavy metals) and diverse neurobiological indicators (such as NfL, GFAP, and sTREM2) measured within those populations across different life stages and follow-up windows. Nonetheless, readers should note that these specific publications do not represent independent lines of epidemiological replication, but rather complementary thematic expansions of identical underlying cohorts.

### 4.8. Future Perspectives: AI, Machine Learning, and the Exposome Framework

The current body of literature maintains a heavy structural bias toward ambient air pollution (48% of included studies), leaving emerging contaminants such as PFAS, pesticides, and complex indoor mixtures largely underrepresented. To operationalize the complexity of multi-chemical and multi-source environmental data, and to shift from single-exposure paradigms toward real-world mixture toxicity, the field of neuroepidemiology is undergoing a profound paradigm shift away from traditional single-pollutant analyses toward integrated models that capture the human exposome. Central to this evolution is the implementation of citizen-cantered engagement integrated with advanced computational architectures. This approach maps complex causal chains linking environmental exposure routes directly to biological responses and downstream neurological outcomes.

The core of this architecture relies heavily on its AI-Epidemiology component. Artificial Intelligence and machine learning algorithms are increasingly recognized as transformative tools in environmental health, uniquely capable of processing high-dimensional datasets derived from human biomonitoring and “omics” technologies [133]. By applying these advanced computational models to multi-matrix panel data, researchers can identify non-linear associations between multi-chemical mixtures and effect biomarkers that often elude traditional, linear statistical methods [134]. Furthermore, research hubs act as a technological engine, providing the necessary infrastructure for federated learning and secure data harmonization. This infrastructure allows for scalable, multi-centre investigations across distinct Areas of High Environmental Risk, facilitating early risk detection in vulnerable populations while strictly ensuring data privacy.

By bridging real-world data from living labs with predictive AI modeling [112], this integrated approach not only advances molecular neurotoxicology but also provides a validated, data-driven blueprint for evaluating environmental policies and timely public health interventions. Importantly, emerging evidence in environmental medicine and nutri-epigenetics indicates that environment-induced biomarker alterations are not static or irreversible. Structural public health policy interventions—such as stringent emission controls—alongside individual-level mitigation strategies (including antioxidant-rich dietary regimens, omega-3 fatty acid supplementation, and active lifestyle modifications) have been shown to attenuate systemic oxidative stress, restore altered DNA methylation patterns, and partially reverse peripheral neuroinflammatory signals [135,136,137,138].

## 5. Conclusions and Future Perspectives

To delineate future research directions, it is essential to address the critical knowledge gaps and toxicological uncertainties identified within the contemporary literature and this scoping review. The current research landscape reflects a pivotal paradigm shift towards population neuroscience, successfully bridging the gap between macro-level environmental data and micro-level cellular models. However, establishing a notable and multidisciplinary framework that integrates toxicology, public health, and environmental and health policy requires a concerted effort to resolve the following key research gaps (Table 3):

- Paucity of longitudinal cohorts: A critical shortage of long-term longitudinal studies restricts the capacity to establish definitive temporal sequences and life-course trajectories regarding pollutant exposure and subsequent neurobiological outcomes.
- Deficits in mixture toxicology data: Current evidence remains predominantly focused on single-pollutant models, highlighting a profound lack of data on the cumulative, synergistic, or antagonistic effects of complex real-world pollutant mixtures.
- Absence of standardised biomarker panels: The field suffers from a lack of harmonised and validated biomarker panels, which severely hinders cross-study comparability, quantitative data synthesis (including targeted meta-analyses of homogeneous cohort subgroups), and the reproducibility of epidemiological findings.
- Geographical underrepresentation in low- and middle-income countries: There is a stark geographical bias, with severely limited evidence originating from low- and middle-income countries, where pollution burdens are often highest but infrastructure for environmental monitoring is lacking. Establishing dedicated population cohorts across these vulnerable regions is urgently needed to map the real-world global landscape of environmental neurotoxicity.
- Suboptimal Integration of multi-omics approaches: Advanced multi-omics methodologies (e.g., genomics, epigenomics, transcriptomics, proteomics, metabolomics) are underutilised, limiting our mechanistic understanding of early biological responses and pathways of toxicity.
- Need for serial biomarker measurements: Existing frameworks rely heavily on single-point sampling, failing to capture the dynamic temporal fluctuations of exposure and the necessity for repeated, longitudinal biomarker assessments over time.

This scoping review underscores the utility of adopting an integrated, exposome-based framework to comprehensively map neurodegenerative and neuropsychiatric risk. By synthesizing evidence from 52 human observational studies across diverse populations and life stages, the included epidemiological data demonstrate that cumulative exposure to ubiquitous environmental contaminants, including ambient fine particulate matter, neurotoxic heavy metals, and persistent organic compounds, is associated with objective, measurable alterations in fluid biomarkers in human populations. Collectively, these findings suggest that the development of pathological phenotypes is not dictated exclusively by immutable genetic programming but may be influenced by modifiable, external environmental determinants operating dynamically across the life course.

While these observational studies document empirical associations with fluid biomarker variations, they do not directly demonstrate underlying cellular or causal mechanisms. The central role played by chronic neuroinflammation and oxidative stress as convergent biological pathways translating exogenous chemical stress into neurological pathology represents a biologically plausible framework supported primarily by broader experimental and toxicological literature. The tracking of highly sensitive fluid biomarkers, most notably NfL for structural axonal degradation, GFAP for reactive astrogliosis, and specific Tau proteoforms for cytostructural alterations—points toward the hypothesis that exposure-related biological alterations may occur during a prolonged, subclinical phase preceding overt clinical symptoms and traditional diagnostic thresholds. Within this context, proposed pathomechanistic cascades must be interpreted as plausible biological models rather than direct empirical proofs derived from observational human data alone.

To overcome current data fragmentation and define precise windows for preventive intervention, the future of environmental neurology relies on a paradigm shift. Future research must prioritize large-scale, prospective longitudinal cohorts that integrate advanced exposomic approaches, high-resolution personal exposure assessments, and multi-omics technologies (spanning genomics, epigenomics, proteomics, and metabolomics). In parallel, rigorous in vitro and in vivo experimental models will be essential to provide translational mechanistic validation for candidate fluid biomarkers and establish a causal link behind the observed epidemiological associations. Systematically deploying standardized, serial multi-biomarker panels across diverse populations—with focused meta-analyses on homogeneous sub-cohorts and expanded biomonitoring in underserved low- and middle-income countries—will be essential to refine risk prediction, move beyond immutable genetic programming, and support timely, evidence-based public health interventions.

## Figures and Tables

**Table 1:** Eligibility criteria based on the four thematic blocks of the search strategy.

Focus	Inclusion Criteria	Exclusion Criteria
Population & Design	Human observational studies (prospective/retrospective cohorts, cross-sectional, case–control) on populations of all ages (including healthy/cognitively unimpaired).	Non-human models (in vivo animal studies, in vitro cell cultures); examination of autopsy materials; secondary literature (reviews, meta-analyses, editorials); case reports.
Environmental Exposure	Quantifiable exposure to ambient pollutants (e.g., PM_2.5_, PM_10_, NO_2_, O_3_, VOCs), heavy metals, pesticides, or persistent contaminants (PFAS, PAHs) across residential, occupational, agricultural, dietary, or hospital-based settings, as well as internal biomonitoring.	Totally qualitative environmental exposure. Acute accidental poisoning.
Pathological Context	Studies investigating neurodegenerative processes, neuroinflammation, neurodevelopmental alterations, or cognitive/mental health decline (including subclinical and preclinical phases).	Studies not relating environmental factors to neurological, neurobiological, or mental health outcomes.
Biological Markers	Objective measurement of biological markers of neurotoxicity, including fluid-based (blood, plasma, serum, CSF), epigenetic (DNA methylation), cellular integrity (TL), or structural/functional parameters.	Studies reporting only clinical symptoms, psychiatric scores, or mortality rates without objective biological marker analysis.

**Table 2:** Summary of key neurological biomarkers associated environmental pollutants, and predominant pathophysiological effects.

Biomarker	Biological Meaning	Most Frequently Associated Pollutants	Predominant Effect	
NfL	Neuroaxonal injury and structural axonal degeneration	PM_2.5_, Cd, Pb, VOCs	Progressive axonal damage, white matter impairment, and accelerated neuroaxonal loss	
GFAP	Reactive astrogliosis and neuroinflammatory response	PM_2.5_, organophosphates	Astrocyte activation, neuroimmune signaling, and blood–brain barrier dysfunction	
sTREM2	Microglial activation and neuroimmune response		PM_2.5_, metal mixtures	Microglial dysfunction, altered phagocytic clearance, and neuroinflammation
Aβ42	Amyloidogenic processing and amyloid plaque accumulation	PM_2.5_, Cd, PFAS	Preclinical Alzheimer’s-like amyloid pathology and extracellular plaque formation	
pTau181/Total Tau	Hyperphosphorylation and neurofibrillary tangle pathology		PM_2.5_, Cd	Tau hyperphosphorylation, cytoskeletal disruption, and Alzheimer’s-like pathology
DNAm	Epigenetic alterations and biological aging acceleration		Air pollution, pesticides, Hg	Epigenetic dysregulation, altered gene expression, and accelerated biological aging

**Table 3:** Key research gaps and evidence limitations identified across major environmental exposure domains in neurodegenerative biomarker research. The volume of available literature for each pollutant category was classified according to predefined quantitative thresholds: Extensive (≥ 15 studies), Moderate (5–14 studies), and Scarce/Emerging (< 5 studies).

Area	Volume Of the Available Literature	Gap
Air pollution	Extensive	Lack of longitudinal studies
Heavy metals	Moderate	Limited CSF biomarkers
PFAS	Scarce	Very small sample sizes
Multi-pollutant exposome	Emerging	Lack of mixture analyses

## Data Availability

Data sharing is not applicable to this article, as no new data were created or analyzed during this study.

## Supplementary Materials

The following supporting information can be downloaded at https://www.mdpi.com/article/10.3390/toxics14090817/s1: Supplementary Document S1: PRISMA-ScR Fillable Checklist; Supplementary Document S2: Search Syntax; Supplementary Document S3: Extracted Data Summary from Included Studies on Ambient Air (*n* = 25); Supplementary Document S4: Extracted Data Summary from Included Studies on Heavy Metals and Trace Elements (*n* = 14); Supplementary Document S5: Extracted Data Summary from Included Studies on Pesticides (*n* = 6); Supplementary Document S6: Extracted Data Summary from Included Studies on Other Environmental Pollutants (*n* = 7); Supplementary Document S7: Exploratory Co-Reporting Analysis of Biomarkers and Environmental Pollutants; Supplementary Table S1: Extracted Data Summary from Included Studies; Supplementary Table S2: Harmonization Dictionary.

## Abbreviations

The following abbreviations are used in this manuscript:

Framework, Guidelines, and Methodologies. PRISMA-ScR: Preferred Reporting Items for Systematic Reviews and Meta-Analyses for Scoping Reviews, PECOS: Population, Exposure, Comparison, Outcome, Study Design, Chemical Substances and Pollutants. PM_2.5_: Particulate matter with a diameter less than 2.5 micrometers, PM10: Particulate matter with a diameter less than 10 micrometers, NO_2_: Nitrogen dioxide, O_3_: Ozone, VOCs: Volatile Organic Compounds, PFAS: Per- and polyfluoroalkyl substances, PAHs: Polycyclic aromatic hydrocarbons, PCBs: Polychlorinated biphenyls, PBDEs: Polybrominated diphenyl ethers, Pb: Lead, Cd: Cadmium, Hg: Mercury, As: Arsenic, Ni: Nickel, Co: Cobalt, Zn: Zinc, SO_2_: Sulfur dioxide, PCDD/Fs: Polychlorinated dibenzo-p-dioxins and dibenzofurans, Biomarkers and Biological Indicators. NfL: Neurofilament light chain, GFAP: Glial fibrillary acidic protein, sTREM2: Soluble triggering receptor expressed on myeloid cells 2, Aβ: Amyloid-beta protein (often specified as Aβ40, Aβ42, or Aβ1-42), Tau: Tau protein (often specified as t-tau or p-tau/p-tau181), BDNF: Brain-derived neurotrophic factor, DNAm: DNA methylation, TDP-43: TAR DNA-binding protein 43, MCP-1: Monocyte chemoattractant protein-1, UCHL1: Ubiquitin carboxy-terminal hydrolase L1, S1P: Sphingosine-1-phosphate, VCAM-1: Vascular cell adhesion molecule-1, Pathologies and Clinical Conditions. AD: Alzheimer’s disease, PD: Parkinson’s disease, MCI: Mild cognitive impairment, ASD: Autism spectrum disorder, ADHD: Attention-deficit/hyperactivity disorder, Other Technical Abbreviations. CSF: Cerebrospinal fluid, PET: Positron emission tomography, BBB: Blood–brain barrier, TL: Telomere length.
