# Supplementary material for: The Exposome–Brain Axis: A Scoping Review of Biological Biomarkers in Environmental Neurotoxicity, Neuroinflammation, and Neurodegeneration
Source: Toxics. 2026 Sep 14;14(9):817. doi: 10.3390/toxics14090817 (PMC13611727; doi:10.3390/toxics14090817)
Supplement: Supplementary file 1 [file toxics-14-00817-s001.zip › Supplementary Document S2 Search Syntax.pdf]

Search Syntax

("air pollution" OR "air pollutants" OR "airborne pollution" OR "urban air pollution" OR "traffic-related air pollution" OR "industrial pollution" OR "heavy traffic" OR "pollution" OR "particulate matter" OR "PM2.5" OR "PM10" OR "fine particles" OR "ultrafine particles" OR "NO2" OR "nitrogen dioxide" OR "SO2" OR "sulfur dioxide" OR "ozone" OR "CO" OR "carbon monoxide" OR "volatile organic compounds" OR "VOCs" OR "benzene" OR "toluene" OR "xylene" OR "BTEX" OR "formaldehyde" OR "polycyclic aromatic hydrocarbons" OR "PAHs" OR "Persistent Organic Pollutants" OR "POPs" OR "pesticides" OR "organochlorine pesticides" OR "organophosphates" OR "per- and polyfluoroalkyl substances" OR "PFAS" OR "heavy metals" OR "lead" OR "cadmium" OR "mercury" OR "arsenic") AND ("Neurodegenerative Diseases" OR "neurodegenerative disease*" OR "neurodegenerative effects" OR "Alzheimer Disease" OR "Alzheimer disease" OR "Alzheimer disease and related dementias" OR "ADRD" OR "dementia" OR "Parkinson Disease" OR "Mental Disorders" OR "Schizophrenia" OR "Bipolar Disorder" OR "Depressive Disorder" OR "Neurodevelopmental Disorders" OR "Autism Spectrum Disorder" OR "ASD" OR "Attention Deficit Hyperactivity Disorder" OR "ADHD" OR "Cognitive Dysfunction" OR "cognitive functioning" OR "cognitive decline" OR "cerebrovascular diseases" OR "brain damage" OR "neural damage" OR "neurodegeneration" OR "neuroinflammation" OR "neuroinflammatory effects″ OR "neuropathological response*" OR "neurobiological mechanism*" OR "neurobiological alteration*") AND ("amyloid beta" OR "Aβ" OR "Aβ40" OR "Aβ42" OR "Aβ1-40" OR "Aβ1-42" OR "Aβ1-42/Aβ1-40" OR "cerebral amyloid load" OR "Centiloid" OR "tau-181" OR "alongside phosphorylated" OR "p-Tau" OR "pTau" OR "t-Tau" OR "tTau" OR "Total Tau" OR "α-Synuclein" OR "CSF α-syn level*" OR "neurofilament light" OR "neurofilament-light" OR "neurofilament light chain" OR "NfL" OR "glial fibrillary acidic protein" OR "GFAP" OR "neuron specific enolase" OR "NSE" OR "protein gene product 9.5" OR "PGP9.5" OR "S100B" OR "S100 calcium binding" OR "protein B" OR "brain derived neurotrophic factor" OR "BDNF" OR "VCAM-1" OR "CSF vascular cell adhesion molecule-1" OR "DNA methylation" OR "DNAm" OR "cerebrospinal fluid" OR "CSF" OR "CSF biomarker*" OR "cerebrospinal fluid (CSF) biomarker*" OR "cerebrospinal fluid–AD biomarker*" OR "brain marker*" OR "brain biomarker*" OR "ADRD biomarker*" OR "neurodegenerative marker*" OR "neurodegenerative biomarker*" OR "neurological marker*" OR "neurological biomarker*" OR "neural damage biomarkers*" OR "neuroinflammation marker*" OR "neuroinflammation biomarker*" OR "neuroinflammatory biomarker*" OR "neuroinflammatory marker*" OR "neuroinflammatory and neurodegenerative plasma biomarkers" OR "neurobiological mechanisms*") AND ("population" OR "population study" OR "population-based cohort" OR "community" OR "community-based investigations" OR "urban population" OR "urban participants" OR "adults" OR "cognitively unimpaired adults" OR "cognitively normal adults" OR "healthy retirees" OR "cohort" OR "cross-sectional" OR "cross sectional" OR "case-control" OR "observational study" OR "epidemiology" OR "residential exposure")

Web of Science: 761 results

("air pollution" OR "air pollutants" OR "airborne pollution" OR "urban air pollution" OR "traffic-related air pollution" OR "industrial pollution" OR "heavy traffic" OR "pollution" OR "particulate matter" OR "PM2.5" OR "PM10" OR "fine particles" OR "ultrafine particles" OR "NO2" OR "nitrogen dioxide" OR "SO2" OR "sulfur dioxide" OR "ozone" OR "CO" OR "carbon monoxide" OR "volatile organic compounds" OR "VOCs" OR "benzene" OR "toluene" OR "xylene" OR "BTEX" OR "formaldehyde" OR "polycyclic aromatic hydrocarbons" OR "PAHs" OR "Persistent Organic Pollutants" OR "POPs" OR "pesticides" OR "organochlorine pesticides" OR "organophosphates" OR "per- and polyfluoroalkyl substances" OR "PFAS" OR "heavy metals" OR "lead" OR "cadmium" OR "mercury" OR "arsenic") AND ("Neurodegenerative Diseases" OR "neurodegenerative disease*" OR "neurodegenerative effects" OR "Alzheimer Disease" OR "Alzheimer disease" OR "Alzheimer disease and related dementias" OR "ADRD" OR "dementia" OR "Parkinson Disease" OR "Mental Disorders" OR "Schizophrenia" OR "Bipolar Disorder" OR "Depressive Disorder" OR "Neurodevelopmental Disorders" OR "Autism Spectrum Disorder" OR "ASD" OR "Attention Deficit Hyperactivity Disorder" OR "ADHD" OR "Cognitive Dysfunction" OR "cognitive functioning" OR "cognitive decline" OR "cerebrovascular diseases" OR "brain damage" OR "neural damage" OR "neurodegeneration" OR "neuroinflammation" OR "neuroinflammatory effects" OR "neuropathological response*" OR "neurobiological mechanism*" OR "neurobiological alteration*") AND ("amyloid beta" OR "Aβ" OR "Aβ40" OR "Aβ42" OR "Aβ1-40" OR "Aβ1-42" OR "Aβ1-42/Aβ1-40" OR "cerebral amyloid load" OR "Centiloid" OR "tau-181" OR "alongside phosphorylated" OR "p-Tau" OR "pTau" OR "t-Tau" OR "tTau" OR "Total Tau" OR "α-Synuclein" OR "CSF α-syn level*" OR "neurofilament light" OR "neurofilament-light" OR "neurofilament light chain" OR "NfL" OR "glial fibrillary acidic protein" OR "GFAP" OR "neuron specific enolase" OR "NSE" OR "protein gene product 9.5" OR "PGP9.5" OR "S100B" OR "S100 calcium binding" OR "protein B" OR "brain derived neurotrophic factor" OR "BDNF" OR "VCAM-1" OR "CSF vascular cell adhesion molecule-1" OR "DNA methylation" OR "DNAm" OR "cerebrospinal fluid" OR "CSF" OR "CSF biomarker*" OR "cerebrospinal fluid (CSF) biomarker*" OR "cerebrospinal fluid–AD biomarker*" OR "brain marker*" OR "brain biomarker*" OR "ADRD biomarker*" OR "neurodegenerative marker*" OR "neurodegenerative biomarker*" OR "neurological marker*" OR "neurological biomarker*" OR "neural damage biomarkers*" OR "neuroinflammation marker*" OR "neuroinflammation biomarker*" OR "neuroinflammatory biomarker*" OR "neuroinflammatory marker*" OR "neuroinflammatory and neurodegenerative plasma biomarkers" OR "neurobiological mechanisms*") AND ("population" OR "population study" OR "population-based cohort" OR "community" OR "community-based investigations" OR "urban population" OR "urban participants" OR "adults" OR "cognitively unimpaired adults" OR "cognitively normal adults" OR "healthy retirees" OR "cohort" OR "cross-sectional" OR "cross sectional" OR "case-control" OR "observational study" OR "epidemiology" OR "residential exposure")

PubMED: 1,004 results

("air pollution"[Title/Abstract] OR "air pollutants"[Title/Abstract] OR "airborne pollution"[Title/Abstract] OR "urban air pollution"[Title/Abstract] OR "traffic-related air pollution"[Title/Abstract] OR "industrial pollution"[Title/Abstract] OR "heavy traffic"[Title/Abstract] OR "pollution"[Title/Abstract] OR "particulate matter"[Title/Abstract] OR "PM2.5"[Title/Abstract] OR "PM10"[Title/Abstract] OR "fine particles"[Title/Abstract] OR "ultrafine particles"[Title/Abstract] OR "NO2"[Title/Abstract] OR "nitrogen dioxide"[Title/Abstract] OR "SO2"[Title/Abstract] OR "sulfur dioxide"[Title/Abstract] OR "ozone"[Title/Abstract] OR "CO"[Title/Abstract] OR "carbon monoxide"[Title/Abstract] OR "volatile organic compounds"[Title/Abstract] OR "VOCs"[Title/Abstract] OR "benzene"[Title/Abstract] OR "toluene"[Title/Abstract] OR "xylene"[Title/Abstract] OR "BTEX"[Title/Abstract] OR "formaldehyde"[Title/Abstract] OR "polycyclic aromatic hydrocarbons"[Title/Abstract] OR "PAHs"[Title/Abstract] OR "Persistent Organic Pollutants"[Title/Abstract] OR "POPs"[Title/Abstract] OR "pesticides"[Title/Abstract] OR "organochlorine pesticides"[Title/Abstract] OR "organophosphates"[Title/Abstract] OR "per- and polyfluoroalkyl substances"[Title/Abstract] OR "PFAS"[Title/Abstract] OR "heavy metals"[Title/Abstract] OR "lead"[Title/Abstract] OR "cadmium"[Title/Abstract] OR "mercury"[Title/Abstract] OR "arsenic"[Title/Abstract]) AND

("Neurodegenerative Diseases"[Title/Abstract] OR "neurodegenerative disease*"[Title/Abstract] OR "neurodegenerative effects"[Title/Abstract] OR "Alzheimer Disease"[Title/Abstract] OR "Alzheimer disease"[Title/Abstract] OR "Alzheimer disease and related dementias"[Title/Abstract] OR "ADRD"[Title/Abstract] OR "dementia"[Title/Abstract] OR "Parkinson Disease"[Title/Abstract] OR "Mental Disorders"[Title/Abstract] OR "Schizophrenia"[Title/Abstract] OR "Bipolar Disorder"[Title/Abstract] OR "Depressive Disorder"[Title/Abstract] OR "Neurodevelopmental Disorders"[Title/Abstract] OR "Autism Spectrum Disorder"[Title/Abstract] OR "ASD"[Title/Abstract] OR "Attention Deficit Hyperactivity Disorder"[Title/Abstract] OR "ADHD"[Title/Abstract] OR "Cognitive Dysfunction"[Title/Abstract] OR "cognitive functioning"[Title/Abstract] OR "cognitive decline"[Title/Abstract] OR "cerebrovascular diseases"[Title/Abstract] OR "brain damage"[Title/Abstract] OR "neural damage"[Title/Abstract] OR "neurodegeneration"[Title/Abstract] OR "neuroinflammation"[Title/Abstract] OR "neuroinflammatory effects"[Title/Abstract] OR "neuropathological response*"[Title/Abstract] OR "neurobiological mechanism*"[Title/Abstract] OR "neurobiological alteration*"[Title/Abstract]) AND ("amyloid beta"[Title/Abstract] OR "Aβ"[Title/Abstract] OR "Aβ40"[Title/Abstract] OR "Aβ42"[Title/Abstract] OR "Aβ1-40"[Title/Abstract] OR "Aβ1-42"[Title/Abstract] OR "Aβ1-42/Aβ1-40"[Title/Abstract] OR "cerebral amyloid load"[Title/Abstract] OR "Centiloid"[Title/Abstract] OR "tau-181"[Title/Abstract] OR "alongside phosphorylated"[Title/Abstract] OR "p-Tau"[Title/Abstract] OR "pTau"[Title/Abstract] OR "t-Tau"[Title/Abstract] OR "tTau"[Title/Abstract] OR "Total Tau"[Title/Abstract] OR "α-Synuclein"[Title/Abstract] OR "CSF α-syn level*"[Title/Abstract] OR "neurofilament light"[Title/Abstract] OR "neurofilament-light"[Title/Abstract] OR "neurofilament light chain"[Title/Abstract] OR "NfL"[Title/Abstract] OR "glial fibrillary acidic protein"[Title/Abstract] OR "GFAP"[Title/Abstract] OR "neuron specific enolase"[Title/Abstract] OR "NSE"[Title/Abstract] OR "protein gene product 9.5"[Title/Abstract] OR "PGP9.5"[Title/Abstract] OR "S100B"[Title/Abstract] OR "S100 calcium binding"[Title/Abstract] OR "protein B"[Title/Abstract] OR "brain derived neurotrophic factor"[Title/Abstract] OR "BDNF"[Title/Abstract] OR "VCAM-1"[Title/Abstract] OR "CSF vascular cell adhesion molecule-1"[Title/Abstract] OR "DNA methylation"[Title/Abstract] OR "DNAm"[Title/Abstract] OR "cerebrospinal fluid"[Title/Abstract] OR "CSF"[Title/Abstract] OR "CSF biomarker*"[Title/Abstract] OR "cerebrospinal fluid (CSF) biomarker*"[Title/Abstract] OR "cerebrospinal fluid–AD biomarker*"[Title/Abstract] OR "brain marker*"[Title/Abstract] OR "brain biomarker*"[Title/Abstract] OR "ADRD biomarker*"[Title/Abstract] OR "neurodegenerative marker*"[Title/Abstract] OR "neurodegenerative biomarker*"[Title/Abstract] OR "neurological marker*"[Title/Abstract] OR "neurological biomarker*"[Title/Abstract] OR "neural damage biomarkers*"[Title/Abstract] OR "neuroinflammation marker*"[Title/Abstract] OR "neuroinflammation biomarker*"[Title/Abstract] OR "neuroinflammatory biomarker*"[Title/Abstract] OR "neuroinflammatory marker*"[Title/Abstract] OR "neuroinflammatory and neurodegenerative plasma biomarkers"[Title/Abstract] OR "neurobiological mechanisms*"[Title/Abstract]) AND ("population"[Title/Abstract] OR "population study"[Title/Abstract] OR "population-based cohort"[Title/Abstract] OR "community"[Title/Abstract] OR "community-based investigations"[Title/Abstract] OR "urban population"[Title/Abstract] OR "urban participants"[Title/Abstract] OR "adults"[Title/Abstract] OR "cognitively unimpaired adults"[Title/Abstract] OR "cognitively normal adults"[Title/Abstract] OR "healthy retirees"[Title/Abstract] OR "cohort"[Title/Abstract] OR "cross-sectional"[Title/Abstract] OR "cross sectional"[Title/Abstract] OR "case-control"[Title/Abstract] OR "observational study"[Title/Abstract] OR "epidemiology"[Title/Abstract] OR "residential exposure"[Title/Abstract])

EMBASE: 1,792 results

('air pollution':ti,ab OR 'air pollutants':ti,ab OR 'airborne pollution':ti,ab OR 'urban air pollution':ti,ab OR 'traffic-related air pollution':ti,ab OR 'industrial pollution':ti,ab OR 'heavy traffic':ti,ab OR 'pollution':ti,ab OR 'particulate matter':ti,ab OR 'pm2.5':ti,ab OR 'pm10':ti,ab OR 'fine particles':ti,ab OR 'ultrafine particles':ti,ab OR 'no2':ti,ab OR 'nitrogen dioxide':ti,ab OR 'so2':ti,ab OR 'sulfur dioxide':ti,ab OR 'ozone':ti,ab OR 'co':ti,ab OR 'carbon monoxide':ti,ab OR 'volatile organic compounds':ti,ab OR 'vocs':ti,ab OR 'benzene':ti,ab OR 'toluene':ti,ab OR 'xylene':ti,ab OR 'btex':ti,ab OR 'formaldehyde':ti,ab OR 'polycyclic aromatic hydrocarbons':ti,ab OR 'pahs':ti,ab OR 'persistent organic pollutants':ti,ab OR 'pops':ti,ab OR 'pesticides':ti,ab

OR 'organochlorine pesticides':ti,ab OR 'organophosphates':ti,ab OR 'per- and polyfluoroalkyl substances':ti,ab OR 'pfas':ti,ab OR 'heavy metals':ti,ab OR 'lead':ti,ab OR 'cadmium':ti,ab OR 'mercury':ti,ab OR 'arsenic':ti,ab) AND ('neurodegenerative diseases':ti,ab OR 'neurodegenerative disease*':ti,ab OR 'neurodegenerative effects':ti,ab OR 'alzheimer disease':ti,ab OR 'alzheimer disease and related dementias':ti,ab OR 'adrd':ti,ab OR 'dementia':ti,ab OR 'parkinson disease':ti,ab OR 'mental disorders':ti,ab OR 'schizophrenia':ti,ab OR 'bipolar disorder':ti,ab OR 'depressive disorder':ti,ab OR 'neurodevelopmental disorders':ti,ab OR 'autism spectrum disorder':ti,ab OR 'asd':ti,ab OR 'attention deficit hyperactivity disorder':ti,ab OR 'adhd':ti,ab OR 'cognitive dysfunction':ti,ab OR 'cognitive functioning':ti,ab OR 'cognitive decline':ti,ab OR 'cerebrovascular diseases':ti,ab OR 'brain damage':ti,ab OR 'neural damage':ti,ab OR 'neurodegeneration':ti,ab OR 'neuroinflammation':ti,ab OR 'neuroinflammatory effects':ti,ab OR 'neuropathological response*':ti,ab OR 'neurobiological mechanism*':ti,ab OR 'neurobiological alteration*':ti,ab) AND ('amyloid beta':ti,ab OR 'ab':ti,ab OR 'ab40':ti,ab OR 'ab42':ti,ab OR 'ab1-40':ti,ab OR 'ab1-42':ti,ab OR 'ab1-42/ab1-40':ti,ab OR 'cerebral amyloid load':ti,ab OR 'centiloid':ti,ab OR 'tau-181':ti,ab OR 'alongside phosphorylated':ti,ab OR 'p-tau':ti,ab OR 'ptau':ti,ab OR 't-tau':ti,ab OR 'ttau':ti,ab OR 'total tau':ti,ab OR 'alpha-synuclein':ti,ab OR 'csf alpha-syn level*':ti,ab OR 'neurofilament light':ti,ab OR 'neurofilament-light':ti,ab OR 'neurofilament light chain':ti,ab OR 'nfl':ti,ab OR 'glial fibrillary acidic protein':ti,ab OR 'gfap':ti,ab OR 'neuron specific enolase':ti,ab OR 'nse':ti,ab OR 'protein gene product 9.5':ti,ab OR 'pgp9.5':ti,ab OR 's100b':ti,ab OR 's100 calcium binding':ti,ab OR 'protein b':ti,ab OR 'brain derived neurotrophic factor':ti,ab OR 'bdnf':ti,ab OR 'vcam-1':ti,ab OR 'csf vascular cell adhesion molecule-1':ti,ab OR 'dna methylation':ti,ab OR 'dnam':ti,ab OR 'cerebrospinal fluid':ti,ab OR 'csf':ti,ab OR 'csf biomarker*':ti,ab OR 'cerebrospinal fluid (csf) biomarker*':ti,ab OR 'cerebrospinal fluid–ad biomarker*':ti,ab OR 'brain marker*':ti,ab OR 'brain biomarker*':ti,ab OR 'adrd biomarker*':ti,ab OR 'neurodegenerative marker*':ti,ab OR 'neurodegenerative biomarker*':ti,ab OR 'neurological marker*':ti,ab OR 'neurological biomarker*':ti,ab OR 'neural damage biomarkers*':ti,ab OR 'neuroinflammation marker*':ti,ab OR 'neuroinflammation biomarker*':ti,ab OR 'neuroinflammatory biomarker*':ti,ab OR 'neuroinflammatory marker*':ti,ab OR 'neuroinflammatory and neurodegenerative plasma biomarkers':ti,ab OR 'neurobiological mechanisms*':ti,ab) AND ('cohort':ti,ab OR 'cross-sectional':ti,ab OR 'cross sectional':ti,ab OR 'case-control':ti,ab OR 'observational study':ti,ab OR 'population study':ti,ab OR 'epidemiology':ti,ab OR 'urban participants':ti,ab OR 'urban population':ti,ab OR 'community-based investigations':ti,ab OR 'cognitively unimpaired adults':ti,ab OR 'cognitively normal adults':ti,ab OR 'healthy retirees':ti,ab OR 'residential exposure':ti,ab OR 'population':ti,ab OR 'adults':ti,ab OR 'community':ti,ab OR 'population-based cohort':ti,ab)

Scopus: 2,378 results

(TITLE-ABS-KEY("air pollution" OR "air pollutants" OR "airborne pollution" OR "urban air pollution" OR "traffic-related air pollution" OR "industrial pollution" OR "heavy traffic" OR "pollution" OR "particulate matter" OR "PM2.5" OR "PM10" OR "fine particles" OR "ultrafine particles" OR "NO2" OR "nitrogen dioxide" OR "SO2" OR "sulfur dioxide" OR "ozone" OR "CO" OR "carbon monoxide" OR "volatile organic compounds" OR "VOCs" OR "benzene" OR "toluene" OR "xylene" OR "BTEX" OR "formaldehyde" OR "polycyclic aromatic hydrocarbons" OR "PAHs" OR "Persistent Organic Pollutants" OR "POPs" OR "pesticides" OR "organochlorine pesticides" OR "organophosphates" OR "per- and polyfluoroalkyl substances" OR "PFAS" OR "heavy metals" OR "lead" OR "cadmium" OR "mercury" OR "arsenic") AND TITLE-ABS-KEY("Neurodegenerative Diseases" OR "neurodegenerative disease*" OR "neurodegenerative effects" OR "Alzheimer Disease" OR "Alzheimer disease" OR "Alzheimer disease and related dementias" OR "ADRD" OR "dementia" OR "Parkinson Disease" OR "Mental Disorders" OR "Schizophrenia" OR "Bipolar Disorder" OR "Depressive Disorder" OR "Neurodevelopmental Disorders" OR "Autism Spectrum Disorder" OR "ASD" OR "Attention Deficit Hyperactivity Disorder" OR "ADHD" OR "Cognitive Dysfunction" OR "cognitive functioning" OR "cognitive decline" OR "cerebrovascular diseases" OR "brain damage" OR "neural damage" OR "neurodegeneration" OR "neuroinflammation" OR "neuroinflammatory effects" OR "neuropathological response*" OR "neurobiological mechanism*" OR "neurobiological alteration*") AND TITLE-ABS-KEY("amyloid beta" OR "Aβ" OR "Aβ40" OR "Aβ42" OR "Aβ1-40" OR "Aβ1-42" OR "Aβ1-42/Aβ1-40" OR "cerebral amyloid load" OR "Centiloid" OR "tau-181" OR "alongside phosphorylated" OR "p-Tau" OR "pTau" OR "t-Tau" OR "tTau" OR "Total Tau" OR "α-

Synuclein" OR "CSF α-syn level*" OR "neurofilament light" OR "neurofilament-light" OR "neurofilament light chain" OR "NfL" OR "glial fibrillary acidic protein" OR "GFAP" OR "neuron specific enolase" OR "NSE" OR "protein gene product 9.5" OR "PGP9.5" OR "S100B" OR "S100 calcium binding" OR "protein B" OR "brain derived neurotrophic factor" OR "BDNF" OR "VCAM-1" OR "CSF vascular cell adhesion molecule-1" OR "DNA methylation" OR "DNAm" OR "cerebrospinal fluid" OR "CSF" OR "CSF biomarker*" OR "cerebrospinal fluid (CSF) biomarker*" OR "cerebrospinal fluid–AD biomarker*" OR "brain marker*" OR "brain biomarker*" OR "ADRD biomarker*" OR "neurodegenerative marker*" OR "neurodegenerative biomarker*" OR "neurological marker*" OR "neurological biomarker*" OR "neural damage biomarkers*" OR "neuroinflammation marker*" OR "neuroinflammation biomarker*" OR "neuroinflammatory biomarker*" OR "neuroinflammatory marker*" OR "neuroinflammatory and neurodegenerative plasma biomarkers" OR "neurobiological mechanisms*") AND TITLE-ABS-KEY("population" OR "population study" OR "population-based cohort" OR "community" OR "community-based investigations" OR "urban population" OR "urban participants" OR "adults" OR "cognitively unimpaired adults" OR "cognitively normal adults" OR "healthy retirees" OR "cohort" OR "cross-sectional" OR "cross sectional" OR "case-control" OR "observational study" OR "epidemiology" OR "residential exposure"))
