# Supplementary material for: The Exposome–Brain Axis: A Scoping Review of Biological Biomarkers in Environmental Neurotoxicity, Neuroinflammation, and Neurodegeneration
Source: Toxics. 2026 Sep 14;14(9):817. doi: 10.3390/toxics14090817 (PMC13611727; doi:10.3390/toxics14090817)
Supplement: Supplementary file 1 [file toxics-14-00817-s001.zip › Supplementary Document S3 Extracted Data Summary from Included Studies on Ambient Air (n = 25).pdf]

First author, year	Country	Study design	Study period	Population	Sample size	Pollutants	Environmental matrix	Exposure assessment	Biomarker	Biological matrix	Confounders	Statistical test	Main results
Zhang ZQ, 2026	China	Cross-sectional study (CABLE cohort)	2017–2024	Cognitively normal adults from northern China evaluated for neurobehavioral and CSF profiles	1,202 participants	PM2.5, O_3, NO_2	Ambient air	2-year average residential exposure before enrolment. PM2.5 modelled via satellite-derived AOD Random Forest grid (1km resolution). O_3 and NO_2 obtained from the GBD 2019 database.	CSF sTREM2, CSF Aβ42, CSF t-tau, CSF p-tau, p-tau/Aβ42 ratio, t-tau/Aβ42 ratio	Cerebrospinal fluid (CSF)	Primary model: Age, sex, education, and APOE ε4 status. Sensitivity models: Smoking, alcohol, BMI, and clinical comorbidities (hypertension, diabetes, hyperlipidaemia, CHD, stroke, atrial fibrillation).	Multiple linear regression models and bootstrap-based mediation analysis	PM2.5 exposures was significantly associated with decreased CSF sTREM2 levels, indicating impaired microglial activation. In downstream structural equation models, this suppression of sTREM2 sequentially mediated the association of PM2.5 with reduced CSF Aβ42 levels (increased amyloid deposition) and elevated p-tau accumulation, which ultimately led to higher depression and anxiety scores. Conversely, O3 and NO2 did not show significant associations with CSF sTREM2.
Liu S , 2026	China	Cross-sectional study (GEHAS prospective cohort)	2023–2024 (follow-up wave used for mental health assessment)	Middle-aged and older adults (aged ≥45 years)	2,271 participants (573 in the DNA methylation subgroup)	Black Carbon (BC)	Ambient air	Short-term average residential exposure calculated across 1, 7, 14, 21, and 28 days prior to survey. Ambient Black Carbon concentrations modelled via the Tracking Air Pollution in China (TAP) database at a 0.1 degree spatial resolution, integrating satellite, ground monitoring, and meteorological data.	DNA methylation (846 CpG sites in SLC25A family genes)	Peripheral blood (genomic DNA)	Age, sex, BMI, smoking status, alcohol consumption, ethnicity, education level, ambient temperature, and estimated peripheral blood leukocyte distribution (estimated via Houseman algorithm).	Linear and logistic regression, restricted cubic spline regression, and mediation analysis	Short-term ambient Black Carbon exposure was significantly associated with increased depression and anxiety symptoms, showing stronger effects at longer exposure windows (28 days). DNA methylation M-values of three specific CpG sites mapped on mitochondrial carrier genes (SLC25A35, SLC25A38, and SLC25A37) partially mediated the association with depressive symptoms only (mediation proportions from 12.19% to 24.84%). No significant mediation was observed for anxiety symptoms. Good sleep quality (PSQI score < 7) significantly mitigated the adverse impacts of Black Carbon on both outcomes.
Balachandar R, 2025	India	Cross-sectional study	Not explicitly stated (published in April 2025)	Healthy adults (41–60 years) residing in a critically polluted area for over 10 years	202 participants (101 men and 101 women)	PM2.5, O3	Ambient air	Time-weighted average annual levels from CPCB stations and 48 seasonal on-site measurements	Amyloid β(1-42), Total τ (Tau), α-Synuclein, BDNF, GFAP	Blood plasma	Age, gender, BMI, blood pressure, random blood sugar, total cholesterol, triglycerides, renal (serum creatinine) and liver function (SGOT); exclusion of	Kolmogorov-Smirnov test (normality), Student's t-test (group comparisons), Pearson correlation (r),	PM2.5 levels consistently exceeded regulatory limits across all seasons. Females exhibited a trend of significantly higher alpha-Synuclein (p<0.001), Amyloid beta (1-42), and GFAP, while males had higher Total tau and BDNF. Older participants (51–60 years) showed higher levels of all biomarkers except alpha-Synuclein. Log-transformed BDNF plasma levels showed a

											prior/current neurological illness	log-transformation for non-normally distributed data, and outlier exclusion	statistically significant inverse correlation with exposure duration (r = -0.189, p = 0.009).
Balachandar R, 2025	India	Cross-sectional comparative study	2023-2025	Long-term adult residents (aged 40-60) from a Critically Polluted Area (CPA) vs geo-demographically matched controls residing at locations with very low / minimal air pollution	405 (203 exposed, 202 controls)	Particulate matter (PM2.5, PM10) and gaseous pollutants (viz. SO2, NO, NO2, NOx, CO, and O3)	Ambient air	Air pollutant levels measured across all seasons following standard protocols (CPCB data and seasonal site measurements)	Amyloid beta 1-42 (Ab1-42), Total Tau (t-tau), alpha-Synuclein (a-syn), Brain-derived neurotrophic factor (BDNF), Glial fibrillary acidic protein (GFAP)	Plasma	Age, gender, BMI, blood pressure, random blood sugar, lipid profile (cholesterol, triglycerides, HDL, LDL, VLDL)	Descriptive statistics, independent t-test/Mann-Whitney U test, Chi-square test, Pearson/Spearman correlation, multivariate logistic regression	Exposed participants had significantly higher levels of GFAP (p<0.001) and Aβ1-42 (p=0.044), and significantly lower levels of total tau, α-synuclein, and BDNF (p<0.001 for all three) compared to controls. Multiple linear regression confirmed that exposure area was a key predictor of biomarker variance independent of metabolic/cardiovascular covariates.
Barber A, 2025	USA	Cross-sectional study	2019	World Trade Center (WTC) responders	970 participants	WTC-related airborne pollutants (dust, debris, and smoke)	Ambient air (dust/smoke at WTC site)	Validated expert-assessed Neurotoxic Exposure Severity Index (5 categories ranging from minimal to severe exposure)	Aβ40 (Normalized Aβ40), Aβ42, pTAU181, GFAP, NfL, and cortical thickness	Plasma	Age, sex/gender, race/ethnicity, education, occupation, supervisor status, PTSD symptom severity, obstructive airway disease, all-cause cancer, blood volume, and cardiovascular disease burden	Spearman's rho (unadjusted correlations) and log-Gamma generalized linear modelling (multivariable-adjusted associations)	Observed Aβ40 levels were lower in minimally exposed WTC responders as compared to severely exposed responders (17.26 vs 44.48%, P=.005). Results remained statistically significant upon adjusting for covariates (adjusted blood volume ratio = 1.11 [1.02-1.22], P=.019). Normalized Aβ40 levels were significantly associated with higher plasma measures of pTAU181, Aβ42, GFAP, and NfL, as well as with reduced cortical thickness in a neuroimaging subsample (rho=-0.29, P=.020).

Ma T, 2025	USA	Population-based cohort study	2016-2020	Middle-aged and older adults free of cognitive impairment at enrollment (Emory Healthy Brain Study)	536 participants	Fine particulate matter (PM2.5)	Ambient air	Spatially matched geocoded residential addresses to the closest centroid of 1-km^2 grids from SEDAC air quality dataset (1-year average preceding blood collection)	Aβ42, and DNA methylation (DNAm) beta values at 9 noteworthy CpG sites (cg21682780, cg00534274 [SLC7A11], cg11676342 [BEND7], cg17904786 [GTF3A], cg23129342, cg10285526, cg05532093 [NFATC1], cg16891376 [FLT3LG], cg05352541 [PITPNB])	Cerebrospinal fluid (CSF) for Aβ42; Whole blood for DNAm	Sex, age, race/ethnicity, educational attainment, BMI, Area Deprivation Index (ADI), three principal components of neighbourhood deprivation, and whole blood cell-type proportions	Linear regression (total effect analysis), Traditional single-mediator mediation analysis (product method), High-dimensional mediation analysis (DACT, HIMA1, HIMA2, HDMA, MedFix), and Causal mediation analysis	Nine CpG sites mediated the PM2.5-Aβ42 association, with significant natural indirect effects (NIEs) validated for eight CpGs individually (mediating 14-43% of the effect). The joint NIE for all nine CpGs was -0.115 (95% CI: -0.215, -0.101) per 1 ug/m^3 increase in PM2.5 exposure , suggesting that differential DNAm (especially in neuroinflammation-related genes like NFATC1) mediates PM2.5 neurotoxicity in preclinical AD stages.
Zhang Y, 2024	China (Beijing)	Cross-sectional neuroimaging and genetic interaction study	2014-2016	Healthy adult volunteers living in Beijing for at least 1 year evaluated for PM2.5 exposure, genetic risk, and neuroimaging	497 participants	Fine particulate matter (PM2.5)	Ambient air	6-month historical ambient exposure assessed based on residential addresses using monthly averages from 12 air monitoring stations in Beijing	Resting-state functional connectivity (rsFC / ReHo) and DNA methylation (cg18219563 in SLC30A3 gene promoter region)	Brain (resting-state fMRI) and Peripheral blood / Whole blood samples	Age, gender, educational attainment, socioeconomic status, and genomic factors (head motion quantified via volume-based framewise displacement)	General linear model (GLM), Partial least square regression (PLSR), Hierarchical regression, Causal mediation analysis, and Nonparametric permutation test	PM2.5 exposures was significantly negatively associated with processing speed and reasoning. In individuals at high genetic risk for depression (higher MDD PRS), PM2.5 exposures significantly reduced local spontaneous activity (ReHo) in the precuneus, which was suggestively linked to the hypermethylation of the SLC30A3 gene. Furthermore, high polygenic risk augmented the impact of PM2.5 on global functional connectivity (Default Mode Network and occipitoparietal network) , where an increased connectivity between the left angular gyrus and left cuneus partially mediated the negative effect of air pollution on processing speed.

Bi Z, 2024	USA	Cross-sectional population study (NHANES)	2013–2014	US adults aged 20 to 75 years old	2008 participants	Volatile organic compounds (VOCs) (28 VOCs analysed)	Indoor/environmental sources (home decoration materials and products)	Whole blood VOC concentration measured via automated capillary gas chromatography and mass spectrometry (GC-MS) with selected-ion monitoring detection and isotope dilution	Neurofilament light chain (NfL)	Serum	Age, sex, ethnicity, BMI, annual household income, systolic blood pressure, urinary creatinine, HbA1c, total cholesterol (TC), PHQ-9 score (depression), cocaine use, and stroke	Multiple linear regression models (variables natural logarithm transformed and standardized), restricted cubic spline (RCS) for non-linear trends, and mediation analysis	Higher levels of 2,5-dimethylfuran (beta=0.042, 95% CI: 0.001, 0.096), ethyl acetate (beta=0.118, 95% CI: 0.008, 0.304), and m-/p-xylene (beta=0.043, 95% CI: 0.012, 0.074) were significantly associated with higher serum NfL levels in the fully adjusted model (Model 4). Serum NfL also had significant mediated effects on the associations of VOCs with depression.
Wang AY, 2024	China	Cross-sectional study (CABLE cohort)	5-year average (study initiated in 2017)	Urban older adults (Han Chinese from Qingdao Municipal Hospital, cognitively normal/excluded severe cognitive impairment)	933 participants	PM2.5, PM10, and NO2	Ambient air	PM2.5: random forest modelling combined with satellite AOD, visibility, meteorological data, and land use parameters (1 km x 1 km resolution). O3 and NO2: Global Burden of Disease 2019 exposure products based on residential addresses.	Alpha-synuclein (a-syn) levels	Cerebrospinal fluid (CSF)	Age, gender, education years, CM-MMSE, BMI, lifestyle factors (cigarette use, alcohol use, physical activity), socioeconomic factors (employment status), and comorbidities (hypertension, diabetes mellitus, hyperlipidemia)	Mann-Whitney U-test, Multivariate linear regression, Restricted cubic spline (RCS) models, Piecewise linear models	Significant negative linear association between PM2.5 and CSF a-syn levels (p=0.029), more pronounced in females, midlife, non-CHD, and cold season subgroups. Significant non-linear inverse J-shaped association for O3 (p for nonlinearity = 0.040), showing a marked decline in CSF a-syn levels when O3 exceeded 37.9 ppb. No statistically significant association for NO2.
Cai X, 2024	China	Cross-sectional Epigenome-Wide Association Study (NSPT cohort)	2015-2019	Han Chinese individuals recruited from different suburban regions, excluding individuals with critical illnesses	3365 participants	Ozone (O3)	Ambient air	Random forest prediction model (risoluzione giornaliera spaziale 1 km x 1 km basata su misurazioni a terra, simulazioni CMAQ, parametri meteorologici, densità di popolazione, altitudine e rete stradale) diviso in 3 finestre temporali: short- (1 mese),	Epigenome-wide DNA methylation (59 CpGs e 30 DMRs a significatività genomica)	Blood (blood-derived DNA, blood leukocytes)	Age, sex, smoking status, smoke pack year, BMI, sampling point index, blood leukocytes fractions, and the first ten genetic principal components (PCs)	Generalized linear model using limma R package for single CpG EWAS; DMRcate and comb-p for DMR analyses; linear regression adjusting for sex, BMI, cell	Identified 59 CpGs and 30 DMRs significantly associated with ozone exposure at a strict genome-wide threshold (P < 5x10^-8). Intermediate and long-term exposure showed an enhanced, gradually shifting hypomethylation trend (including UBE2G2 gene), while short-term methylation changes did not persist. Targeted genes were enriched in biological pathways involving telomere maintenance, DNA damage response, and megakaryocyte differentiation, linking exposure to aging, immune/inflammatory disease, metabolic syndrome,

								intermediate- (1 anno) e long-term (10 anni)				proportion, and smoking for global/regional tests	neurodevelopmental disorders, oncogenesis, and thrombosis.
Casey E, 2024	USA	Cross-sectional study	2016-2020	Cognitively healthy adults (45-75 years old) free of cognitive impairment at enrollment	1,113 (ambient analysis); 1,080 (traffic-related subset)	Fine particulate matter (PM2.5), both ambient and traffic-related	Ambient air	Geocoded residential addresses matched to 1-km ambient PM2.5 estimates (ensemble model) and 200-250 m traffic-related PM2.5 (random forest model) averaged for 1, 3, and 5 years prior to collection	Amyloid-beta 42 (Aβ42), total Tau (tTau), phosphorylated Tau (pTau), tTau/Aβ42, and pTau/Aβ42	Cerebrospinal fluid (CSF)	Age, gender, race, ethnicity, body mass index (BMI), and neighborhood socioeconomic status (Area Deprivation Index)	Multiple linear regression and multiple logistic regression	IQR increases in 1-year (β = -0.101; 95% CI: -0.18, -0.02) and 3-year (β = -0.078; 95% CI: -0.15, -0.00) ambient PM2.5 were significantly and negatively associated with CSF Aβ42. 5-year ambient PM2.5 showed a similar trend (β = -0.076; 95% CI: -0.160, 0.005). Traffic-related PM2.5 showed similar but non-significant trends. Associations with tTau, pTau, and ratios were mostly null.
Ma YH, 2023	China	Prospective cohort (CLHLS) and biomarker study (CABLE)	2008-2018	Older adults from the Chinese Longitudinal Healthy Longevity Survey (CLHLS) and cognitively unimpaired older adults from the Chinese Alzheimer's Biomarker and Lifestyle (CABLE) study	31,573 participants total (CLHLS: 31,573; CABLE: 1,131; Longitudinal sub-cohort: 5,878)	PM2.5, O3, NO2	Ambient Air	Satellite-based random-forest machine learning model (approx. 1 km x 1 km resolution) for PM2.5, and Global Burden of Disease 2019 exposure products for O3 and NO2. Assigned by residential geocodes as 2-year averages preceding enrolment.	Amyloid-beta (Abeta42, Abeta40), Total tau (T-tau), Phosphorylated tau (P-tau), and their ratios (Abeta42/Abeta40, P-tau/Abeta42, T-tau/Abeta42)	Cerebrospinal fluid (CSF)	Age, sex, educational attainment, APOE e4 status, lifestyle factors, socioeconomic factors, and medical comorbidities (hypertension, diabetes, cerebrovascular disease, cardiovascular disease)	Cox proportional hazards models, Logistic regression models, Linear models, and Mediation models (10,000 bootstrapped iterations)	Long-term exposure to PM2.5 (per 20 ug/m3) increased the risk of cognitive impairment (HR: 1.100; 95% CI: 1.026-1.180). High PM2.5 exposures was significantly associated with greater brain amyloid load in CSF (lower Abeta42 and Abeta42/Abeta40; higher P-tau/Abeta42 and T-tau/Abeta42 ratios). Amyloid pathology partly mediated the PM2.5-associated global cognitive decline (mediation proportions: 16.95% to 21.64%). No significant longitudinal associations were found for O3, NO2, or standalone tau proteins in sensitivity analyses.
Hajat A, 2023	United States	Prospective cohort (GEM study)	2000–2008	Dementia-free older adults aged 75 years or older recruited from four US sites	3044	PM2.5, PM10, NO2	Ambient air	Spatiotemporal model applied to residential addresses (incorporating address histories from LexisNexis dating back to	Amyloid beta (Aβ1-40, Aβ1-42, and Aβ1-42/Aβ1-40 ratio)	Plasma	ge, sex, race, education, study site, treatment assignment, Cystatin C, neighborhood deprivation index,	Ordinary least squares (OLS) linear regression for cross-sectional	Cross-sectional analysis found small positive associations between pollutants and Aβ1-40, but null for Aβ1-42 or ratio. Repeated measures analysis showed stronger positive associations across all three pollutants and outcomes: a 2 μg/m³ higher 20-year average PM2.5 was

				(Winston-Salem, NC; Hagerstown, MD; Sacramento, CA; Pittsburgh, PA)				1980) to predict 1-, 5-, 10-, and 20-year exposure averages			smoking status, pack-years smoking, secondhand smoke exposure, alcohol consumption, physical activity, APOE ε4 carrier status, hypertension, BMI	analysis; linear random effects models for repeated measures analysis	associated with 4.43% higher Aβ1-40, 9.73% higher Aβ1-42, and 1.57% higher Aβ1-42/Aβ1-40 ratio. Results for PM10 and NO2 were similar.
Qu R, 2023	China	Panel study	2018-2019	Healthy elderly adults	34 participants	Ozone (O3)	Ambient air	Calculated based on outdoor monitoring data (hourly concentrations from an automatic air monitoring system on campus) and sampling times.	Brain-derived neurotrophic factor (BDNF), Neurofilament light chain (NfL), Neuron-specific enolase (NSE), Protein gene product 9.5 (PGP9.5), S100 calcium-binding protein B (S100B)	Serum	Time trends (natural cubic smooth function of the day within the study with 3 degrees of freedom), average temperature, relative humidity, sex, age, body mass index (BMI), allergic status, and season.	Linear mixed-effects (LME) model	Short-term O3 exposure was positively associated with serum concentration of neural damage biomarkers. A 10 $\mu g/m^3$ increase in O3-8h was associated with an increment of 74% (95% CI: 1%-146%) in BDNF at lag 2 d and 197% (95% CI: 39%-356%) in NfL at lag 1 d. No significant associations were found for NSE, PGP9.5, or S100B.
Choi YJ, 2023	Republic of Korea	Prospective mother-child cohort	2012–2015 (recruitment at birth) with regular follow-up at 2-year intervals up to 8 years of age	Mother-child pairs recruited after birth, regularly evaluated for ADHD symptoms at ages 4, 6, and 8 years; a sub-cohort was tracked repeatedly for genome-wide DNA methylation	329 mother-child pairs for the main analysis; 51 children completed full evaluation within the DNA methylation sub-cohort	Sulfur dioxide (SO2)	Ambient air	District-specific monthly ambient SO2 levels from 257 national monitoring stations, assigned to the 1st, 2nd, and 3rd trimesters of pregnancy using ArcGIS based on the Euclidean distance from residential addresses	Genome-wide DNA methylation (DNAm) analyzed via candidate CpG approach (focusing on 6 specific loci, including cg07583420 within *INS-IGF2*) and epigenome-wide association study (EWAS)	Whole blood (sampled at ages 2 and 6 years)	Maternal age at pregnancy, mother's education level, maternal smoking status during pregnancy, environmental tobacco smoking (ETS) during pregnancy, child's sex, gestational age, multiple births, season of child's birth, cell type fraction, and maternal IQ	Linear regression, multivariable linear regression, Weighted Gene Co-methylation Network Analysis (WGCNA), and mediation analysis	Prenatal SO2 exposure during the 1st trimester was significantly associated with an 8.38% increase in the ADHD rating scale (ARS) at age 4. Exposure during the 3rd trimester contributed to differential DNA methylation at age 2 (specifically cg07583420 within INS-IGF2), which was persistently linked with increased ARS scores at ages 4, 6, and 8. Mediation analysis confirmed a significant indirect effect of 3rd-trimester prenatal exposure on ARS scores through DNA methylation at ages 4 and 6. Postnatal SO2 exposure was not significantly associated with changes in DNA methylation.

Urbano T, 2023	Italy	Prospective cohort study	2008 to 2021 (MCI diagnosis between 2008-2014, followed up until August 2021)	Subjects newly-diagnosed with mild cognitive impairment (MCI) living in the Modena and Reggio Emilia provinces	53	PM10 (Particulate matter < 10 μm) from motorized traffic	Ambient air	Spatial proximity analysis within a geographical information system (GIS) environment; ground-level concentrations modeled using California LINE version 4 (CALINE4) air quality dispersion model based on 2006 traffic and census data, validated with monitoring stations	Amyloid beta 1-42 (Aß1-42), total tau (t-tau), phosphorylated tau (p-tau181)	Cerebrospinal fluid (CSF)	Age, sex, and educational attainment (main multivariable model); additional model adjusted for electromagnetic fields (distance from power lines/transformers), selenate, baseline MMSE score, smoking status, diabetes, and COPD	Cox proportional hazards model combined with a restricted cubic spline regression model	During a median follow-up of 47.3 months, 34 of 53 participants converted to dementia (26 Alzheimer's). In non-linear spline analysis, mean and maximum annual PM10 levels positively correlated with CSF total and phosphorylated tau, and inversely correlated with Aß1-42. A non-linear increased risk of dementia conversion was found above 10 $\mu g/m^3$ for mean annual PM10 levels and above 35 $\mu g/m^3$ for maximum annual PM10 levels.
Honkova K, 2022	Czech Republic	Cross-sectional comparative study	2017–2019	Male police officers working outdoors	125 nonsmoking city policemen	PM2.5, benzo(a)pyrene, NO2	Air (outdoor occupational exposure)	Stationary air pollution monitoring data from the Czech Hydrometeorological Institute	Genome-wide DNA methylation (differentially methylated CpG loci)	Blood	Age, BMI, smoking status (all non-smokers), occupation duration, season/locality	ANOVA, PCA, differential methylation analysis with FDR-adjusted p-values, gene set enrichment analysis	No season-specific DNA methylation pattern was identified. Significant differences in DNA methylation profiles were observed mainly between Ostrava and Prague, with 13,643 differentially methylated CpG loci. Altered loci were linked to genes associated with diabetes mellitus (KCNQ1), respiratory diseases (PTPRN2), dopaminergic/neurodegenerative pathways (NR4A2), and DNA repair (XRCC5). The most affected pathway was axon guidance. Air pollution type and source were associated with distinct genome-wide DNA methylation patterns.
Song J, 2022	China	Panel study	2018–2019	Healthy retired adults from Xinxiang Medical University	34 participants	PM2.5 and PM2.5 constituents (24 metals/nonmetals and 6 ions)	Ambient air	Hourly mean concentration from an air quality monitoring station	BDNF, NfL, NSE, PGP9.5, S100B	Serum	Age, sex, BMI, allergic status, cold during follow-up, season, relative humidity, temperature, time trends; two-pollutant models additionally adjusted for NO2, SO2, O3, CO	Linear mixed-effect model with random intercept; lag analyses; stratified analyses	Short-term PM2.5 exposures was significantly associated with increased neural damage biomarkers. A 10 μg/m3 increase in PM2.5 was associated with increases in BDNF (2.09%; 95% CI: 39.3–76.5%), NfL (100%; 95% CI: 1.73–198%), and PGP9.5 (122%; 95% CI: 20.7–222%) on the current day. Several PM2.5 constituents (Cu, Zn, Ni, Mn, Sn, V, Rb, Pb, Al, Be, Cs, Co, Th, U, Cl−, F−) were positively associated with NfL. GSTT1 polymorphism modified PM2.5-associated neural damage effects.

Calderón-Garcidueñas L, 2022	Mexico	Prospective cohort study (clinical component)	2004–2008 (cohort monitoring period)	Cognitively healthy children and adults, plus patients diagnosed with Amyotrophic Lateral Sclerosis (ALS), residing in highly polluted Metropolitan Mexico City (MMC) and low-pollution control cities.	194 clinical subjects (92 MMC children, 26 control children, 43 MMC adults, 14 control adults, and 19 ALS patients).	PM2.5 (Fine particulate matter) and UFPM (Ultrafine particulate matter)	Ambient air	Residential history linked with data from air quality monitoring stations.	TDP-43	Cerebrospinal fluid (CSF) obtained via lumbar puncture.	History of head trauma/sports, maternal smoking/alcohol/illicit drugs, socioeconomic status, and indoor pets	Two-sample t-test, linear regression analysis	In living subjects, lumbar CSF TDP-43 concentrations increased exponentially with age and were significantly higher in young MMC residents highly exposed to PM2.5 and UFPM compared to cohorts living in low-pollution control cities
Li M, 2022	China	Cross-sectional analysis within an ongoing large-scale cohort study (The CABLE study).	Ongoing since 2017	Cognitively intact Han Chinese older adults recruited from Qingdao Municipal Hospital.	1,131 cognitively normal individuals overall (final sample sizes: 1,031 for sTREM2 analysis, 307 for hsCRP, and 735 for peripheral immune cells).	Fine particulate matter (PM2.5), Nitrogen dioxide (NO2), and Ozone (O3).	Ambient air	2-year averaged ambient levels estimated at each participant's residence using satellite-based high-resolution models for PM2.5 and Global Burden of Disease (GBD) exposure products for O3 and NO2.	Soluble TREM2 (sTREM2), core AD biomarkers (Amyloid β42 [Aβ42], total tau [t-tau], phosphorylated tau [p-tau]), and inflammatory markers (hsCRP, peripheral immune cell counts).	Cerebrospinal fluid (CSF) obtained via lumbar puncture (for sTREM2, Aβ42, t-tau, p-tau) and fasting blood samples (for hsCRP and immune cells).	Adjusted for age, gender, education, and APOE-ε4 status (fundamental models) , with sequential adjustments for lifestyle/socioeconomic characteristics (BMI, smoking, drinking, physical activity, occupation), clinical comorbidities (stroke, diabetes, coronary heart disease, hypertension), and CSF pathology levels.	Multivariable linear regression, multivariate logistic regression, multi-pollutants modeling, and mediation analysis with bootstrapped resamples.	Ambient 2-year averaged PM2.5 exposures was significantly associated with decreased levels of the neuroinflammatory marker CSF sTREM2 ($\beta = -0.116$, $p = 0.0002$), indicating microglial dysfunction. Furthermore, mediation analysis demonstrated that CSF sTREM2 acted as a key mediator, modulating the total harmful effects of PM2.5 on Alzheimer's disease amyloid pathologies (explaining 14.22% of the effect on Aβ42 and 47.15% on p-tau/Aβ42). Similar inverse associations with sTREM2 were observed for O3 among elderly and urban subgroups.
Alemany S, 2021	Spain	Cross-sectional analysis within a nested	2013–2019	Cognitively unimpaired adults with increased risk of Alzheimer's	156 participants	Nitrogen dioxide (NO2), PM2.5, PM10, and PM2.5 absorbance	Ambient air	Residential long-term exposure estimated via Land Use Regression (LUR) models from the ESCAPE campaign	CSF Aβ42, Aβ40, Aβ42/40 ratio, p-tau, t-tau, NfL, and brain Centiloid values	Cerebrospinal fluid (CSF) and brain tissue (PET imaging)	Sex, age at recruitment, APOE-ε4 status, education, physical activity, BMI, smoking,	Multiple linear regression models and interaction analyses	Long-term exposure to NO2 and PM2.5 absorbance was associated with greater brain Aβ deposition (mainly in Aβ-positive individuals), while PM10 and PM2.5 exposures were linked to higher CSF NfL levels, especially in APOE-ε4 carriers.

		longitudinal study (The ALFA+ Study)		disease residing in Barcelona							and family history of AD		
Iaccarino L, 2021	USA	Cross-sectional study (Secondary analysis of the IDEAS cohort)	2016–2018 (with air data from 2002–2003 and 2015–2016)	Older adults (aged ≥65) with cognitive impairment (MCI or dementia)	18,178 patients (10,991 MCI and 7,187 dementia)	Fine particulate matter (PM2.5) and ground-level ozone (O3)	Ambient air	Residential estimates calculated via the EPA Downscaler model at the Census tract level	Brain amyloid-beta (Aβ) plaque deposition (PET scan positivity)	Brain tissue (Positron Emission Tomography imaging)	Age, sex, race/ethnicity, income, smoking, clinical stage, and extensive medical comorbidities (cardiovascular, respiratory, etc.)	Logistic regression models, Average Marginal Effects (AME), and interaction analyses (FDR-corrected)	Higher long-term and short-term exposure to ambient PM2.5 was significantly associated with an increased, dose-dependent risk of brain amyloid-beta plaque deposition, whereas ground-level O3 showed no association with PET scan positivity.
Lee JH, 2020	South Korea	Cross-sectional study (KBASE cohort)	April 2014 to November 2016	309 older adults (191 cognitively normal and 118 with MCI) without dementia	309 participants	PM10	Ambient air	5-year mean concentration of PM10 matched to residential address	Cerebral amyloid-beta (Aβ) positivity (via PiB-PET), tau accumulation (via 18F-AV-1451 PET), AD signature cortical thickness, white matter hyperintensity (WMH) volume	Brain (cerebral)	Age, sex, education level, annual income, vascular risk score, smoking status, APOE ε4 positivity, and cognitive status (CN or MCI)	One-way analysis of variance, linear-by-linear association test, multivariate linear or logistic regression	Exposure to the highest tertile of PM10mean was associated with increased risk of amyloid-β (Aβ) positivity (OR 2.19, 95% CI 1.13 to 4.26). No significant associations were found between PM10mean exposure and tau accumulation, AD signature cortical thickness, or white matter hyperintensity volume.
Shaffer RM, 2019	USA	Observational cohort study	2001-2012	Community volunteers at VA Puget Sound (stratified by cognitive status: cognitively normal and MCI/AD)	133 total (73 cognitively normal, 60 MCI/AD)	Fine particulate matter (PM2.5)	Ambient air	Short-term (7-day daily average from central monitor) and long-term (1-year average from national model predictions)	VCAM-1 and e-selectin	Liquido cerebrospinale (CSF)	Age, smoking status, sex, education, APOE e4 status, BMI, hypertension, coronary heart disease, diabetes	Multivariable linear regression	This study provides evidence that in cognitively normal individuals, both short-term (7-day) and long-term (1-year) fine particulate matter exposure are associated with increased concentrations of CSF VCAM-1, with 1-year exposure also linked to elevated CSF e-selectin; however, these associations were not observed or remained inconclusive among participants diagnosed with MCI or AD.

Calderón-Garcidueñas L, 2012	Mexico	Matched cohort study (1-year longitudinal follow-up)	2007 (baseline) to 2008 (follow-up)	Healthy children matched by age and socioeconomic status (lifelong severe air pollution exposure in Mexico City vs. clean-air controls)	30 children (10 MC WMH+, 10 MC WMH-, and 10 matched controls)	Fine particulate matter (PM2.5) and Ozone (O_3)	Ambient air	Fixed-site monitoring stations (inclusion criteria required lifelong residency within 5 miles of the city monitoring stations)	Tumor Necrosis Factor alpha (TNF-alpha), Monocyte Chemoattractant Protein-1 (MCP-1), Chemokine CC motif ligand 22 (CCL22), Granulocyte Colony-Stimulating Factor (G-CSF), and Interleukin 12 (IL-12 p40)	Peripheral blood (serum)	Age, socioeconomic status, and similar Body Mass Index or height, while controlling for smoking history, environmental tobacco exposure, full-term birth, and unremarkable clinical histories	Mixed-model ANOVAs with full factorial design, focused polynomial orthogonal quadratic contrasts, and equivalent t-test contrasts	Compared to MC WMH-, MC WMH+ children showed significant compensatory grey and white matter volume increases in temporal, parietal, and frontal regions, resulting in better cognitive performance on related subscales (Arithmetic, Digit Span, Picture Arrangement, Object Assembly, Mazes) comparable to CTL. Inflammation-wise, MC WMH- children exhibited a strict pro-inflammatory profile with higher TNF-alpha, MCP-1, and IL-12 p40 alongside lower neuroprotective CCL22 and G-CSF , whereas MC WMH+ children displayed an immunoregulatory and tissue remodeling profile.
