# Supplementary material for: The Exposome–Brain Axis: A Scoping Review of Biological Biomarkers in Environmental Neurotoxicity, Neuroinflammation, and Neurodegeneration
Source: Toxics. 2026 Sep 14;14(9):817. doi: 10.3390/toxics14090817 (PMC13611727; doi:10.3390/toxics14090817)
Supplement: Supplementary file 1 [file toxics-14-00817-s001.zip › Supplementary Document S4 Extracted Data Summary from Included Studies on Heavy Metals and Trace Elements (n = 14).pdf]

First author, year	Country	Study design	Study period	Population	Sample size	Pollutants	Environmental matrix	Exposure assessment	Biomarker	Biological matrix	Confounders	Statistical test	Main results
Sarhan A A, 2026	Egypt	Case-control hospital-based study	2022-2023	Patients aged 50+ with neurological disorders (mean age = 68.16 ± 7.32 years) and healthy controls (mean age was 65.47 ± 10.15 years)	86 (43 AD cases, 43 controls)	Toxic heavy metals, Pesticides, Diesel motor exhaust	Occupational and environmental exposure	Risk factors questionnaire (closed-ended questions)	Serum neurofilament light chain (sNFLc)	Serum (Blood)	Gender (found non-significant), Age, Education, Residence, Marital status	Chi-square, t-test, Mann-Whitney, Spearman correlation, Binary logistic regression, ROC curve	sNFLc levels were significantly higher in AD patients (median 49.21) vs controls (34.91); sNFLc shows promise as a predictor for AD (AUC 0.866).
Wang X, 2025	USA	Longitudinal cohort study (SWAN)	2003–2004	Midlife women (median age = 53.3 years) tracked longitudinally from menopausal transition to late-midlife	194 participants	Serum levels of essential trace elements (manganese, iron, cobalt, copper, zinc, selenium, and molybdenum)	Mixed environmental matrices / Diet	Internal dose tracking via direct quantification of element concentrations in whole blood using ICP-MS	Serum Amyloid-beta (Aβ42, Aβ40, Aβ42/Aβ40 ratio), total tau, p-tau181	Whole blood (baseline) and serum (follow-up)	Age, race/ethnicity, education, site, BMI, smoking, alcohol, physical activity, and APOE ε4 status	Multivariable linear regression and elastic net penalized mixture regression	Higher midlife blood Zinc levels were significantly associated with a higher, protective serum Aβ42/Aβ40 ratio 20 years later, whereas an elevated baseline Cu/Zn ratio correlated with a lower, pathological amyloid ratio, indicating that midlife element homeostasis impacts long-term amyloidogenesis.
Li Y, 2025	China	Cross-sectional occupational study	Not specified	Male coal miners from Shanxi province evaluated for occupational metal exposure and cognitive decline (MCI vs. controls)	376 participants (159 MCI cases, 217 controls)	Metal mixture of 16 urinary elements (including Al, V, Cr, Mn, Fe, Co, Ni, Cu, Zn, As, Se, Sr, Cd, Sb, Tl, Pb)	Mixed environmental matrices / Occupational dust	Internal dose tracking via direct quantification of 16 metal concentrations in spot urine samples using ICP-MS	Plasma sTrem2, Neurofilament Light chain (NfL), phosphorylated Tau (p-tau181), and Montreal Cognitive Assessment (MoCA) scores	Spot urine and blood plasma	Age, education, years of employment, BMI, smoking status, alcohol consumption, and history of hypertension	Multivariable logistic regression, Propensity Score Matching, BKMR, and quantile g-computation (qgcomp)	Occupational exposure to the metal mixture (driven primarily by Ni and Co) significantly increased the risk of Mild Cognitive Impairment (MCI) and elevated plasma sTrem2 levels, validating sTrem2-mediated neuroinflammation as a key pathway linking metal toxicity to cognitive decline.

Wang Y, 2025	USA	Cross-sectional population study (NHANES)	2013–2014	General US adult population evaluated for multi-metal exposure and serum neurodegenerative biomarkers	833 participants	Twelve urinary metal levels: barium (Ba), cadmium (Cd), cobalt (Co), cesium (Cs), molybdenum (Mo), lead (Pb), antimony (Sb), tin (Sn), strontium (Sr), thallium (Tl), tungsten (W) and uranium (U)	Mixed environmental matrices	Internal dose tracking via direct quantification of metal concentrations in whole blood and spot urine samples using ICP-MS	Serum Neurofilament Light chain (sNfL) concentrations	Whole blood, spot urine, and blood serum	Age, sex, race/ethnicity, education, income-to-poverty ratio, smoking, alcohol, BMI, physical activity, waist circumference, and eGFR	Multivariable linear/logistic regression, WQS regression, and BKMR models	Exposure to the 12-metal mixture was significantly and positively associated with elevated serum sNfL levels, particularly in older and male participants. In the positive-effect WQS model, urinary Cd was the predominant driver of this neurotoxic association, accounting for 69.6% of the total mixture weight, followed by Pb (8.9%). In the separate negative-effect WQS model, urinary Ba (30.6%) and Tl (29.3%) were the main contributors to the inverse weight. BKMR analysis confirmed the overall linear positive joint effect of the mixture when all metals were above their 50th percentile.
Ehlinger J V, 2025	Mexico	Prospective birth cohort (ELEMENT cohort)	Recruitment: 1994-2004; Childhood visit: 2008-2012; Adolescent visit: starting 2015	Children and adolescents (9–17 years old)	N = 399 for the main model (Hg and CPT3); N = 374 for the interaction model with DNAm	Mercury (Hg)	Not applicable (Biomonitoring focus)	Mercury concentrations measured in maternal blood (2nd trimester) and child blood (at the 9–17 years visit) via ICP-MS	DNA methylation (DNAm) at 12 specific CpG sites (e.g., in genes like GRIN2B, BDNF, SLC6A4)	Leukocyte DNA from child blood	Age, sex, prenatal lead (Pb) exposure, prenatal tobacco smoke exposure, maternal education, and blood cell type proportions	Linear regression, interaction models, and False Discovery Rate (FDR) correction (Benjamini-Hochberg)	Significant interactions were found between blood mercury levels and DNA methylation on sustained attention. Specifically, for CpG sites in the GRIN2B and BDNF genes, the association between mercury and ADHD-related metrics (Omissions/Commissions) varied significantly depending on methylation levels.
Zhou Y, 2025	USA	Cross-sectional study	2013-2014	US adults (Total population for sNfL analysis; adults aged >= 60 for cognitive function tests)	959 participants	Lead (Pb), Cadmium (Cd), Mercury (Hg), Manganese (Mn), Selenium (Se)	None (Blood samples utilized directly for environmental exposure levels)	Inductively Coupled Plasma Mass Spectrometry (ICP-MS) technology	Serum neurofilament light chain (sNfL)	Blood (whole blood for heavy metals, serum for NfL)	Sex, age, race, education, marital status, poverty-to-income ratio (PIR), body mass index (BMI), metabolic equivalents (MET), alcohol consumption, smoking status	Generalized linear models (GLMs), weighted quantile sum regression (WQS), Bayesian kernel machine regression (BKMR), quantile-based g computation (qgcomp),	Positive correlation between blood Pb or Cd and sNfL in total population. Mixtures showed strong correlation with increased sNfL (Cd primary positive factor; Mn, Se negative factors). Significant negative relationship between sNfL or Cd and cognitive scores (AFT, DSST) in adults aged >= 60. sNfL partially mediated the association between Cd and AFT (23.35%) or DSST (32.7%).

												restricted cubic splines (RCS), and mediation analysis	
Urbano T, 2024	Italy	Cross-sectional study	2019-2021	Newly diagnosed patients with mild cognitive impairment (MCI)	128 patients (89 with serum samples, 45 with CSF samples analyzed)	Cadmium, copper, iron, manganese, zinc (Trace elements)	Environmental / Occupational (General background exposure)	Measurement of serum and cerebrospinal fluid (CSF) concentrations using ICP-MS / ICP-AES	β-amyloid 1-40, β-amyloid 1-42 (amyloid ratio), Total Tau, phosphorylated Tau181 (p-Tau181), and Neurofilament light chain (NfL)	Serum and cerebrospinal fluid (CSF)	Age at diagnosis, sex, educational attainment, and Mini-Mental State Examination (MMSE) score	Linear fit models with marginal statistics and restricted cubic spline regression models	High serum cadmium concentrations were monotonically associated with selected biomarkers of cognitive impairment (inversely associated with amyloid ratio and positively associated with Tau proteins). Other trace elements (Cu, Fe, Mn, Zn) displayed complex, heterogeneous, and mostly non-linear associations across serum and CSF matrices.
Luo J, 2024	USA	Cross-sectional population study (NHANES)	2013–2014	General US adult population evaluated for environmental cadmium exposure and axonal injury	1,040 participants	Cadmium (Cd)	Diet, water, air, tobacco (general environmental exposure)	Whole blood cadmium measurement	Neurofilament light chain (NfL)	Serum	Age, gender, race, education level, BMI, smoking status, alcohol drinking status, diabetes, and cardiovascular disease (CVD)	Linear regression and restricted cubic spline (RCS) models	Blood cadmium levels were positively associated with serum NfL (beta = 3.35, 95% CI: 0.41, 6.30, p for trend = 0.014 in the fully adjusted model). A linear dose-effect relationship was observed (p for non-linearity = 0.15), and this significant positive association was present only in middle-aged and older adults (50-75 years).
Qing Y, 2024	China	Cross-sectional study	2021	Seniors aged over 60 years in Shanghai	836 participants	Lead (Pb), Cadmium (Cd), Mercury (Hg)	Mixed environmental matrices	Urinary and blood concentration measurements using ICP-MS	Amyloid beta 42 (Abeta42), Amyloid beta 42/40 ratio (Abeta42/40), Total tau (T-tau), Phosphorylated tau (P-tau), Malondialdehyde (MDA), Superoxide dismutase (SOD), Catalase (CAT),	Plasma, Whole blood, Morning urine	Age, Gender, Body Mass Index (BMI), Education, Household income, Marital status, Tobacco smoking, Alcohol consumption	Mann-Whitney U test, Chi-square test, Spearman correlation analysis, Multiple linear regression, Weighted quantile sum (WQS) regression, Nonparametric bootstrapping	Urinary Pb and Cd levels were significantly negatively correlated with MoCA score, Abeta42, and Abeta42/40 ratio. Urinary Cd, Hg, and blood Hg were significantly positively correlated with P-tau. Combined metal exposure (WQS index) showed a more significant effect on cognitive impairment than individual exposure. Plasma SOD was found to partially mediate the effects of urinary Cd on Abeta42/40 and P-tau, accounting for approximately 20% of the total effect.

									Glutathione peroxidase (GSH-Px)			mediation modelling	
Wu L, 2023	China	Cross-sectional occupational study	2019	Welders exposed to welding fumes	165 (186 recruited, 21 excluded due to missing serum sample information)	Multiple heavy metals (Li, V, Cr, Mn, Fe, Co, Ni, Cu, Zn, As, Se, Sr, Mo, Cd, W, Pb)	Occupational environment (welding processes)	Inductively coupled plasma mass spectrometry (ICP-MS) of morning urine samples	Neurofilament light chain (NfL), Sphingosine-1-phosphate (S1P), Prolactin (PRL), Dopamine (DA)	Serum	Age, sex, BMI, education, smoking, and alcohol drinking	Multivariable linear regression, Bayesian kernel machine regression (BKMR), Quantile g-computation (QG-C)	Inverted U-shaped associations of urinary Ni with NfL, S1P, and DA observed in BKMR; non-linear relationship between Fe and PRL. Urinary Co was positively associated with PRL (strongest positive weights in QG-C). Urinary Pb was associated with higher S1P levels. Urinary As was associated with higher NfL and S1P, and lower PRL. Interactions were found among Ni, Zn, As, Sr, Fe, and Pb on neural damage biomarkers. Zn showed potential neuroprotective effects (negatively associated with NfL and S1P).
Zaw YH, 2019	Thailand	Cross-sectional study	June to October 2018	Myanmar migrant pregnant women (age 18–35, 0–14 weeks of gestation)	108 participants	Lead (Pb), Mercury (Hg), Cadmium (Cd), Arsenic (As)	Not applicable (Biomonitoring focus)	Maternal blood heavy metals measured by inductively coupled plasma mass spectrometer (ICP-MS)	Brain-derived neurotrophic factor (BDNF) serum levels	Maternal whole blood (for metals) and plasma (for BDNF)	Age, BMI, secondhand smoke exposure, regular aerobic exercise	Multivariate binary logistic regression, Mann-Whitney U test, Spearman Rank correlation, Independent t-test, Chi-square	High blood total arsenic concentration was associated with 2.6-fold increased odds (aOR=2.603, 95% CI: 1.178–5.751) of low plasma BDNF. No significant association found between BDNF and Pb, Hg, or Cd.
de Paula Fonseca Arrifano G, 2018	Brazil	Cross-sectional epidemiological study	Not explicitly stated (Received October 2017, Accepted	Riverine populations within the State of Pará, Brazilian Amazon (adult consumers of large quantities of fish)	224 individuals evaluated initially for anthropometric data and symptoms	Methylmercury (MeHg) and Total Mercury (THg)	None (Exposure via contaminated fish consumption was confirmed, but environmenta	Hair mercury speciation (analysis of MeHg and inorganic mercury in hair samples collected from the occipital region)	S100B mRNA expression levels	Whole blood (leukocytes) and hair samples	Age, sex, Body Mass Index (BMI), pre-existent chronic diseases (e.g., cancer, diabetes, cardiovascular diseases, hypertension, inflammatory diseases, arthritis), smoking	D'Agostino-Pearson normality test, Student's t-test, Mann-Whitney U test, Cumulative odds ordinal logistic regression with	Approximately 20% of the participants showed total hair mercury levels above the recommended limit of 10 μg/g. No direct quantitative correlation was found between mercury exposure levels and clinical self-reported symptoms. However, individuals with high mercury levels (≥10 μg/g) presented S100B mRNA blood levels over two times higher than those with lower exposure (<10 μg/g). A significant positive correlation was established between hair mercury content and S100B

			July 2018)		, and a sub-sample of 24 individuals included for the final S100B mRNA qPCR analysis after applying strict exclusion criteria.		l matrices like water or soil were not directly sampled in this study)				habits, alcohol drinking, drug dependency, chronic pharmaceutical treatments, recent head/body traumas, and inflammation status (verified via C-reactive protein)	proportional odds, Fisher's exact test, Spearman correlation test, and Pearson's partial correlation.	mRNA levels in blood, supporting its potential use as a peripheral candidate biomarker to predict mercury-induced neurotoxicity in humans.
Li Y, 2016	USA	Prospective birth cohort study (longitudinal follow-up)	1979–1984 (birth of participants) to 2008–2010 (adult follow-up)	Adults (mean age 27 years) who were enrolled at birth in the Cincinnati Lead Study	105 participants (64 females, 41 males)	Lead (Pb)	Not applicable (exposure assessed via blood)	Serial blood lead level measurements from maternal blood (1st and 2nd trimester) and children's blood (at 10 days of age, every 3 months up to 60 months, and every 6 months from 60 to 78 months)	DNA methylation at differentially methylated regions (DMRs) of human imprinted genes	Peripheral blood leukocytes (for DNA methylation in adulthood); maternal peripheral blood and child's whole blood (for lead levels)	Batch effect (first or second) and maternal cigarette smoking during pregnancy	Adjusted linear regression models	Early childhood lead exposure was associated with sex-dependent and gene-specific DNA methylation differences in adulthood. Specifically, higher childhood lead levels were associated with decreased PEG3 DMR methylation (significantly in males) and decreased IGF2/H19 DMR methylation (significantly in females). Conversely, higher neonatal lead exposure was associated with higher PLAGLI/HYMAI DMR methylation regardless of sex.
Mazumdar M, 2012	USA	Prospective birth cohort study (longitudinal follow-up)	1979 – 2009 (Infants were enrolled between August 1979 and April	Young adults (aged 28–30 years) who were enrolled as new-borns in the Boston prospective cohort study	55 participants for plasma analysis; 36 participants for gene	Lead (Pb)	Not reported (General environmental lead exposure)	Umbilical cord blood lead concentrations at birth, capillary blood lead concentrations at ages 6, 12, 18, and 24 months, and venous blood lead concentrations at 57 months and 10 years, measured via graphite	Plasma concentrations of amyloid beta proteins (Ab40 and Ab42) and blood AD-related gene expression levels (ADAM9, RTN4, LRPAP1)	Cord blood, capillary blood, venous blood, plasma, and whole blood (for RNA extraction)	Demographics, medical history, family medical history (parental dementia), concurrent medications (oral contraceptives), alcohol and tobacco use, maternal medication, maternal alcohol and	Correlation coefficients, linear regression models, robust multichip analysis (RMA), Benjamini-Hochberg correction for	Mean plasma Ab42 concentrations were lower among participants with high umbilical cord blood lead concentrations (greater than or equal to 10 ug/dL) than in participants with lower cord blood lead concentrations (p = 0.08). Among participants with high prenatal lead exposure, evidence of an inverse relationship was found between umbilical cord lead concentration and expression of ADAM9, RTN4, and LRPAP1 genes in adulthood.

			1981 and followed up into young adulthoo d in 2009)		expression analysis			furnace atomic absorption spectrometry			tobacco use during pregnancy, maternal IQ, age, body mass index, weeks of gestation, and birth weight	false discovery rate (FDR), and hypergeometric test.	
