# Supplementary material for: The Exposome–Brain Axis: A Scoping Review of Biological Biomarkers in Environmental Neurotoxicity, Neuroinflammation, and Neurodegeneration
Source: Toxics. 2026 Sep 14;14(9):817. doi: 10.3390/toxics14090817 (PMC13611727; doi:10.3390/toxics14090817)
Supplement: Supplementary file 1 [file toxics-14-00817-s001.zip › Supplementary Document S5 Extracted Data Summary from Included Studies on Pesticides (n = 6).pdf]

First author, year	Country	Study design	Study period	Population	Sample size	Pollutants	Environmental matrix	Exposure assessment	Biomarker	Biological matrix	Confounders	Statistical test	Main results
Hernandez AF, 2025	Spain	Prospective birth cohort study	2014-2017	Mother-child pairs recruited at delivery from university hospitals in Granada	398 mother-child pairs	Organophosphate compounds (OPCs): Organophosphate pesticides (OPPs) and Organophosphate flame retardants (OPFRs)	Mixed environmental matrices (dietary/indoor dust)	Internal maternal dose quantified via urinary metabolites (DAPs for pesticides, mOPFRs for flame retardants) at delivery	Cord blood concentrations of Glial Fibrillary Acidic Protein (GFAP), Ubiquitin C-terminal Hydrolase L1 (UCHL1), and S100B	Maternal urine and umbilical cord blood	Maternal age, smoking, education, parity, infant sex, gestational age, and mode of delivery	Multivariable linear regression and restricted cubic spline models (sex-stratified analyses)	Prenatal exposure to organophosphates significantly elevated molecular biomarkers of neonatal neurotoxicity, showing distinct sex-specific profiles: maternal pesticide metabolites linked to elevated cord GFAP (primarily in males), while flame retardant metabolites linked to increased GFAP and UCHL1 (predominantly in females).
Gong Y, 2025	USA	Population-based case-control study (PEG cohort)	2000–2007 (PEG 1) and 2010–2016 (PEG 2)	Residents of Central Valley, California (Kern, Tulare, or Fresno County) with early-stage Parkinson's disease (PD) and elderly community controls	806 participants (569 Parkinson's cases, 237 controls)	Copper pesticides (15 specific copper chemicals evaluated)	Air (Ambient agricultural application near homes and workplaces)	Geographic Information System (GIS)-based model utilizing California Pesticide Use Reporting (CA-PUR) system data, calculating applied pounds within a 500m radius buffer around lifetime addresses history from 1974 to the blood draw year	Genome-wide DNA methylation status (EWAS at CpG sites) and gene pathway enrichment profiles	Peripheral whole blood	Age at blood draw, sex, European ancestry, smoking status, study wave, cell composition (CD8T, CD4T, NK, Bcell, neutrophil, granulocytes), and organophosphates (OP) exposure	Epigenome-wide association analysis (EWAS) using empirical Bayes linear model (WGCNA and ChAMP R-packages), Bumphunter algorithm, Benjamini-Hochberg correction for multiple testing, and Bayesian correction via 'bacon' R-package to control genomic inflation	Long-term ambient exposure to copper pesticides resulted in differential DNA methylation in blood. Identified 140 CpG sites and 40 regions (top probe: cg17591573 in the *COL11A2* gene; $r=-0.16$, $p=8.84\times10^{-9}$). Enriched pathways include Fc gamma R-mediated phagocytosis ($p=6.03\times10^{-5}$), homophilic cell adhesion ($p=2.27\times10^{-7}$), and EGF receptor signaling, all of which are linked to neuroinflammation and neurodegeneration in PD pathology.

Schaffner S L, 2024	France, USA, Australia, New Zealand	Multi-cohort case-control study (epigenome-wide association study [EWAS])	1998 - 2000	Agricultural workers (TERRE cohort) and clinic-based patients (DIGPD replication cohort)	Discovery (TERRE): 71 cases, 147 controls; Replication (DIGPD): 110 cases, 112 controls	Pesticides (including insecticides and fungicides)	Occupational/ Household environment	Self-report questionnaires and occupational health interviews with physicians	Blood DNA methylation patterns (specifically reference co-methylated regions or CMRs)	Whole blood	Age, head trauma, alcohol consumption, smoking, blood cell type principal components (PCs 1–6), and genotype principal components (PCs 1–3)	Linear regression analysis, robust linear modeling (rlm), t-test with Benjamini-Hochberg (BH) adjustment, and Akaike Information Criterion (AIC)	Identified 69 differentially methylated regions (CMRs) associated with early-stage PD in females but only 2 in males. Genetic variation in *cis* (SNP genotype) significantly explained interindividual variation in DNAm at these regions, more so than pesticide exposure alone.
Maggio A G, 2022	Denmark	Cross-sectional analysis within a cohort study (High vs. low exposure groups)	2007–2008	Young men born between 1981 and 1984 recruited from the general population register	52 participants (26 high exposure; 26 low exposure)	1,1-dichloro-2,2-bis(p-chlorophenyl)ethylene (DDE)	Diet (Pilot whale meat and blubber)	Measurement of DDE concentrations in serum (divided into tertiles to compare highest vs. lowest exposure groups)	Differentially methylated regions (DMRs) / DNA methylation signatures	Sperm (for DNA methylation), Serum (for exposure measurement)	Height, weight, age, smoking status, sample collection date, length of abstinence, and genetic background	dmrseq (for differential methylation), Fisher's exact test (for pathway enrichment), linear regression/correlation (for validation)	Elevated exposure to DDE was significantly associated with genome-wide differentially methylated regions (DMRs) in sperm. The DMR-associated genes showed significant enrichment for neurological functions and neurodevelopmental processes, and overlapped significantly with known autism spectrum disorder (ASD) risk genes, indicating a potential epigenetic link between organochlorine exposure and neurodevelopmental risk.
Paul C K, 2018	USA	Population-based cross-sectional study (PEG cohort)	2000–2015 (PEG1: 2000–2007; PEG2: 2010–2015)	Parkinson's disease (PD) patients and population-based controls from agricultural regions of the California Central Valley (Kern, Tulare, and Fresno counties)	839 participants in total (580 peripheral blood samples: 342 PD patients and 238 controls; 259 saliva samples: 128 PD	Organophosphate (OP) pesticides (ambient exposure to 36 individual OP chemicals)	Air / Ambient agricultural drift	Geographic Information System (GIS)-based model linking California state mandated pesticide use reports (CA-PUR), land use surveys for crop locations, and geocoded residential/occupational addresses within a 500-m buffer	Genome-wide DNA methylation profiles (70 differentially methylated CpG sites)	Peripheral whole blood and saliva	Age, sex, European ancestry, Parkinson's disease status, and blood cell composition (controlled as a secondary analysis)	Chi-square test, Pearson correlation, biweight midcorrelation, regression models (residuals analysis), Student's t-test, and Fisher's exact test (for gene set	Long-term ambient organophosphate exposure was significantly associated with differential DNA methylation at 70 CpG sites in 41 genes across both blood and saliva matrices. The differentially methylated gene set was highly enriched for the nicotinic and muscarinic acetylcholine receptor signalling pathways, which align directly with the biological targets of organophosphate toxicity. Global mean methylation levels decreased in PD patients as organophosphate exposure increased.

					patients and 131 controls)							overrepresentation analysis)	
Brighina L, 2008	Italy	Case-control study	After June 1, 1996	Parkinson's disease patients and healthy controls	833 case-control pairs (833 cases and 833 controls; total 1,666 subjects)	Pesticides (including herbicides, insecticides, and fungicides)	None / Not applicable (exposure was assessed via questionnaire, no environmental samples were collected)	Telephone interviews using a structured risk factors questionnaire administered directly to the subjects or via proxy.	SNCA (Alpha-synuclein) gene polymorphisms (REP1)	Venous blood (genomic DNA extracted from leukocytes)	Age, sex, education level, smoking history, respondent type (direct vs. proxy), and control type (siblings vs. unrelated population controls)	Conditional logistic regression, Cox proportional hazard models, kappa statistics, and intraclass correlation coefficients.	Increasing SNCA REP1 bp length was associated with an increased risk of Parkinson disease (OR = 1.18 per unit, p = 0.03). Pesticide exposures overall (OR = 1.80, p = 0.01) and herbicides specifically (OR = 2.46, p = 0.004) were significantly associated with Parkinson disease in younger subjects (age ≤ 59.8 years) only. SNCA REP1 genotype score and herbicide exposures showed independent effects on the risk of Parkinson disease, with no significant pairwise interactions.
