# Supplementary material for: The Exposome–Brain Axis: A Scoping Review of Biological Biomarkers in Environmental Neurotoxicity, Neuroinflammation, and Neurodegeneration
Source: Toxics. 2026 Sep 14;14(9):817. doi: 10.3390/toxics14090817 (PMC13611727; doi:10.3390/toxics14090817)
Supplement: Supplementary file 1 [file toxics-14-00817-s001.zip › Supplementary Document S6 Extracted Data Summary from Included Studies on Other Environmental Pollutants (n = 7).pdf]

First author, year	Country	Study design	Study period	Population	Sample size	Pollutants	Environmental matrix	Exposure assessment	Biomarker	Biological matrix	Confounders	Statistical test	Main results
Delcourt N, 2024	France	Proof-of-concept study (Pilot study)	2021	Older adult patients (aged 68.3 to 86.7) hospitalized with suspected normal pressure hydrocephalus (NPH)	8	Per- and polyfluoroalkyl substances (PFAS) (18 PFAS analyzed, including PFHxS, PFOA, PFOS, PFBS, PFHpS)	N/A (The study focuses strictly on the internal exposure through biological fluids)	Quantification in CSF using liquid chromatography electrospray ionization mass spectrometry (LC-ESI(-)-MS/MS) after protein precipitation and extraction	Amyloid-beta (Ab42, Ab40), Total Tau (tTau), Phosphorylated Tau (pTau)	Cerebrospinal fluid (CSF)	Presence of comorbidities (e.g., renal failure) monitored	Descriptive statistics (mean, SD), Non-parametric comparison tests (alpha = 5%)	PFHxS, PFOA, and PFOS were found in all CSF samples. Patients with both cognitive impairment (MMSE <= 25) and an Ab1-42/40 ratio < 0.1 showed significantly higher concentrations of total PFAS, total PFOS, branched PFOS, and PFHxS in the CSF compared to patients with only one or no signs.
Mouat JS, 2023	USA	Prospective birth cohort (MARBLES)	2006-2016	Mother-child pairs at increased risk of Autism Spectrum Disorder (ASD) (mothers who already had a biological child with ASD)	156 mother-child pairs (MARBLES study sample analysed: 104 maternal serum samples and 147 placental tissue samples, with 95 overlapping pairs having both matrices)	Polychlorinated biphenyls (PCBs)	Maternal serum	Gas chromatography-tandem mass spectrometry (GC-MS/MS) of maternal serum collected at delivery	Placental DNA methylation (Whole Genome Bisulfite Sequencing, WGBS) analyzed via co-methylation networks/modules	Maternal serum (at delivery) and placenta tissue	Maternal age, year of sample collection, pre-pregnancy BMI, and maternal plasma polyunsaturated fatty acids (PUFA) levels	Weighted Gene Correlation Network Analysis (WGCNA) via Comethyl R package, Kendall's tau for left-censored data, bicor (biweight midcorrelation), multiple linear regression, Peto-Prentice log-rank test, and logistic regression	Placental co-methylation modules significantly correlated with maternal serum PCBs and child neurodevelopment. Specifically, two modules mapping to ASD-related genes *CSMD1* and *AUTS2* negatively correlated with maternal serum PCB levels as well as child neurodevelopmental assessments (ADOS comparison score and Mullen Scales of Early Learning composite score). No direct population-level association was observed between maternal serum PCB levels and categorical child ASD diagnosis.

Mattonet K, 2022	Germany	Prospective birth cohort (RAPS)	2000-2002	Healthy German or Turkish-speaking mother-infant pairs, born at term (weeks 38-42 of pregnancy), APGAR score >= 8, parity 1-3, without serious complications or illnesses during pregnancy/parturition, and without congenital anomalies.	142 mother-infant pairs (73 females, 69 males)	Polychlorinated biphenyls (PCBs), polychlorinated dibenzo-p-dioxins and dibenzofurans (PCDD/Fs)	Air / Industrial dust leakage (leakage of one ton of dioxinated dust from a recycling firm in 1999)	Quantification of 16 congener levels from maternal blood taken during late pregnancy (28th–42nd week, median: 32 weeks) using capillary gas chromatography and high-resolution mass spectrometry	DNA methylation (DNAm) levels assessed across the genome at around 850,000 CpG sites	Cord blood	Smoking status of mother during pregnancy, age of mother at birth, gestational age, weight of neonate, technical batch (EPIC assay plate), and estimated cell-type proportions (CD4T, CD8T, natural killer cells, B cells, granulocytes, monocytes)	Sex-stratified Epigenome-Wide Association Studies (EWAS) using multiple regression models with outlier-robust empirical Bayes moderation (via limma), regional analysis (via dmrCate), and Weighted Gene Correlation Network Analysis (WGCNA)	Prenatal low-dose exposure to PCBs and PCDD/Fs is significantly associated with altered DNA methylation patterns in cord blood, showing marked sex-specific effects. The EWAS identified 32 significantly differentially methylated positions (DMPs) and 8 differentially methylated regions (DMRs) mapping to genes explicitly involved in neurodevelopment, gene regulation, and immune functioning.
Agin A, 2020	France	Follow-up of a cohort / Case-control study	Not specified	Patients with Dementia with Lewy bodies (DLB) at prodromal or demented stages, and patients with Alzheimer's disease (AD) at prodromal or demented stages (alphaLewyMA cohort)	45 patients (24 DLB: 16 prodromal, 8 demented; 21 AD controls: 8 prodromal, 13 demented)	Phthalates / Bis(2-ethylhexyl) phthalate (DEHP)	Indoor/outdoor environment (general background environmental exposure)	Indirect environmental history / Non-targeted and targeted LC-MS metabolomic screening	Bis(2-ethylhexyl) phthalate (DEHP) levels, core CSF biomarkers (Total Tau, Ph-tau, A-beta 1-42, A-beta 1-40)	Cerebrospinal fluid (CSF)	Baseline demographics evaluated (age, sex, MMSE), but no multivariate regression adjustment performed	Mann-Whitney U test, Welch's t-test, Fisher's exact test, Univariate analysis (volcano plots)	Non-targeted metabolomics revealed a significantly higher concentration of the phthalate DEHP in the CSF of patients with DLB compared to AD controls (2.7-fold in the whole cohort, p=0.031; 3.8-fold in the demented subgroup, p=0.001). Targeted screening for pesticides showed no significant differences.

Wang Y, 2020	USA	Prospective birth cohort study	1998–2006	Pregnant women and their children (Columbia Center for Children's Environmental Health - CCCEH MN cohort)	328 (discovery MN cohort), 43 (replication Sibling cohort) for ADHD; 216 (discovery), 94 (replication) for MDI	Polycyclic Aromatic Hydrocarbons (PAHs)	Ambient air	48-hour personal prenatal air monitoring during the third trimester	DNA methylation profiles (445 CpG sites across 25 genes) and neurodevelopmental scores	Umbilical cord blood leukocytes (DNAm) and clinical developmental tracking at age 3	Not specified	Weighted Kernel Distance-Based Method (WKDM) and multi-locus ridge regression	The study identified significant gene-level associations and interactions between DNA methylation and prenatal PAH exposure with neurodevelopmental outcomes (ADHD and MDI) at age 3. Specific genes (e.g., CYP2E1, LOC84931) were identified and replicated.
Perera F, 2018	China	Prospective sequential birth cohort study	2002 and 2005	Newborns of non-smoking mothers	255 (122 in 2002 cohort, 133 in 2005 cohort)	Polycyclic Aromatic Hydrocarbons (PAHs)	Ambient air	Prenatal exposure assessed via proxy using molecular PAH-DNA adduct levels quantified in umbilical cord blood	Relative Telomere Length (TL) and Brain-Derived Neurotrophic Factor (BDNF) levels	Umbilical cord blood leukocytes (for TL) and cord blood plasma (for BDNF)	Mother's education, mother's age, gestational age, child's sex, cord mercury	Multiple regression, t-test, chi-square test	Mean TL was significantly higher in the second cohort; PAH-DNA cord adducts were significantly and inversely correlated with TL; TL was not a mediator between adducts and developmental scores.
Abou-Donia M B, 2018	USA	Case-control study	10-year clinical evaluation period	Patients with chronic neurological symptoms and histories of chemical or mold exposures vs. healthy controls	36 participants in total (24 toxicant-exposed patients and 12 healthy controls)	Contaminated drinking water, indoor mold/water damage, pesticides, and assorted toxic chemicals	Air / Water / Environmental matrix	Detailed clinical and occupational history tracking with estimation of major exposures	Neural autoantibodies (Tau, MAP-2, NFP, MBP, GFAP, and tubulin) and S-100B control levels	Serum	Age and gender used for stratification, and exclusion of patients with a history of stroke, traumatic brain injury, Parkinson's, or Alzheimer's disease	Descriptive antibody fold-level profiling and clinical subgroup stratification	Chemically/mold-exposed patients had significantly higher levels of all six neural autoantibodies compared to controls ($p < 0.001$), except for S-100B. Mean antibody levels relative to controls in descending order were: tau (475%), MAP-2 (391%), NFP (334%), MBP (302%), GFAP (299%), and tubulin (225%). Specific symptoms correlated significantly with distinct autoantibodies: tau was elevated in neuropathy, muscle/joint pain, asthma, and chemical sensitivity; tubulin was higher in chemical sensitivity and asthma; NFP was higher in sleep apnea; and S-100B was increased in muscle/joint pain, asthma, and apnea/insomnia.
