# Supplementary material for: The Exposome–Brain Axis: A Scoping Review of Biological Biomarkers in Environmental Neurotoxicity, Neuroinflammation, and Neurodegeneration
Source: Toxics. 2026 Sep 14;14(9):817. doi: 10.3390/toxics14090817 (PMC13611727; doi:10.3390/toxics14090817)
Supplement: Supplementary file 1 [file toxics-14-00817-s001.zip › Supplementary Document S7 Exploratory Co-Reporting Analysis of Biomarkers and Environmental Pollutants.pdf]

Exploratory Co-Reporting Analysis of Biomarkers and Environmental Pollutants

To complement the narrative synthesis and summary tables, an exploratory co-reporting analysis was performed using information extracted from the included studies. The aim was to provide a structured overview of recurring "patterns" of pollutants and biomarkers across the reviewed literature.
Specifically, the analysis aimed to: (i) identify recurring biomarker groups across the included studies; (ii) identify recurring pollutant groups; (iii) explore how pollutant and biomarker terms were co-reported within the same studies and (iv) assess whether the main descriptive co-reporting patterns persisted when a more restrictive occurrence threshold was applied.
The analysis was conducted using the data extraction table developed for the review, with each included study treated as the unit of analysis. Biomarker and pollutant terms were extracted from the corresponding fields and harmonized before network construction to reduce fragmentation due to spelling variants, abbreviations, synonyms, or/and alternative nomenclature. Harmonization was performed upstream of VOSviewer directly in the analysis dataset; the original extracted terms were retained alongside their harmonized counterparts for traceability. A complete harmonization dictionary, reporting one original term or variant per row together with the corresponding harmonized term, is provided in the Supplementary Material. , Network construction and visualization were performed using VOSviewer version 1.6.20. Co-occurrence analysis was based on author keywords using the full counting method. Link strengths were normalized using the association-strength method, and clusters were identified using the VOS clustering technique with a resolution parameter of 1.00 and a minimum cluster size of one; the option to merge small clusters was enabled. The clustering optimization settings were 10 random starts, 10 iterations, and a random seed of 0. All components satisfying the occurrence threshold were retained; the networks were not restricted to the largest connected component. Three complementary analyses were conducted: (1) a biomarker-only co-reporting map, (2) a pollutant-only co-reporting map, and (3) a combined pollutant-biomarker co-reporting map. In the combined map, terms were prefixed (Bio_ and Exp_) to distinguish biomarker from pollutant/exposure terms. A minimum occurrence threshold of two (2) was used for the primary analysis, and a threshold of three (3). Links between terms indicate that two terms were reported within the same study. Accordingly, the maps represent study-level co-reporting patterns and should not be interpreted as evidence of causal, statistical, or mechanistic associations.

Structured overview of recurring "patterns" of pollutants and biomarkers across the reviewed literature

The included studies were highly heterogeneous in exposure assessment, biomarker selection, biological matrices, study populations, and analytical approaches. Although this heterogeneity could limit the feasibility of a quantitative synthesis, it provided an opportunity to describe how environmental pollutants and biomarkers were distributed and co-reported across the 52 included studies.

In the network maps:

1. Each node represents a biomarker or an environmental pollutant;
2. The size of a node is proportional to the number of studies in which the term appears;
3. A link between two nodes indicates that the two terms were reported within the same study;
4. The strength of a link increases with the number of studies in which the two terms were reported together;
5. In the VOSviewer layout, the distance between nodes approximately reflects their normalized relatedness based on co-reporting; closer nodes generally indicate stronger normalized co-reporting relationships.

Colors identify groups of terms that tend to appear together more frequently. These groups, referred to as clusters, should be interpreted as descriptive groupings within the reviewed literature rather than as established biological pathways or causal mechanisms.

Across the three exploratory analyses, increasing the minimum occurrence threshold from two to three reduced the number of retained terms, while the main descriptive co-reporting patterns remained visible.

This comparison was used as a descriptive sensitivity check to assess the persistence of the observed structure after including lower-frequency terms; it was not intended as a formal test of robustness.

Analysis	Primary threshold	Sensitivity threshold	Main interpretation
Biomarker-only	21 items / 4 clusters	17 items / 3 clusters	Main biomarker domains remained visible
Pollutant-only	29 items / 7 clusters	17 items / 4 clusters	Metal and air-pollution domains remained visible
Pollutant-biomarker	50 items / 4 clusters	34 items / 4 clusters	Main cross-domain co-reporting structure remained visible

The biomarker-only map showed a structured pattern of co-reporting among neurodegeneration- and neuroinflammation-related biomarkers. At the primary threshold, 21 biomarker terms were retained and grouped into four clusters. The most connected terms included Aβ42, total tau, p-tau, Aβ40, NfL and DNA methylation.

The sensitivity analysis using a threshold of three retained 17 terms and three clusters. Low-frequency and peripheral terms were removed, while the main amyloid/tau, neural injury/glial-neurotrophic and epigenetic domains remained visible. This indicates persistence of the main descriptive biomarker co-reporting pattern after the threshold was increased.

Dominant terms	Conservative descriptive label
Aβ40, Aβ42, p-tau, total tau, Aβ ratios, sTREM2	Amyloid/tau biomarker cluster
NfL, GFAP, BDNF, S100B, alpha-synuclein, NSE, PGP9.5	Neural injury / glial / neurotrophic biomarker cluster
DNA methylation, CpG sites, DMRs, EWAS	Epigenetic biomarker cluster
Amyloid PET positivity, cortical thickness	Neuroimaging-related terms

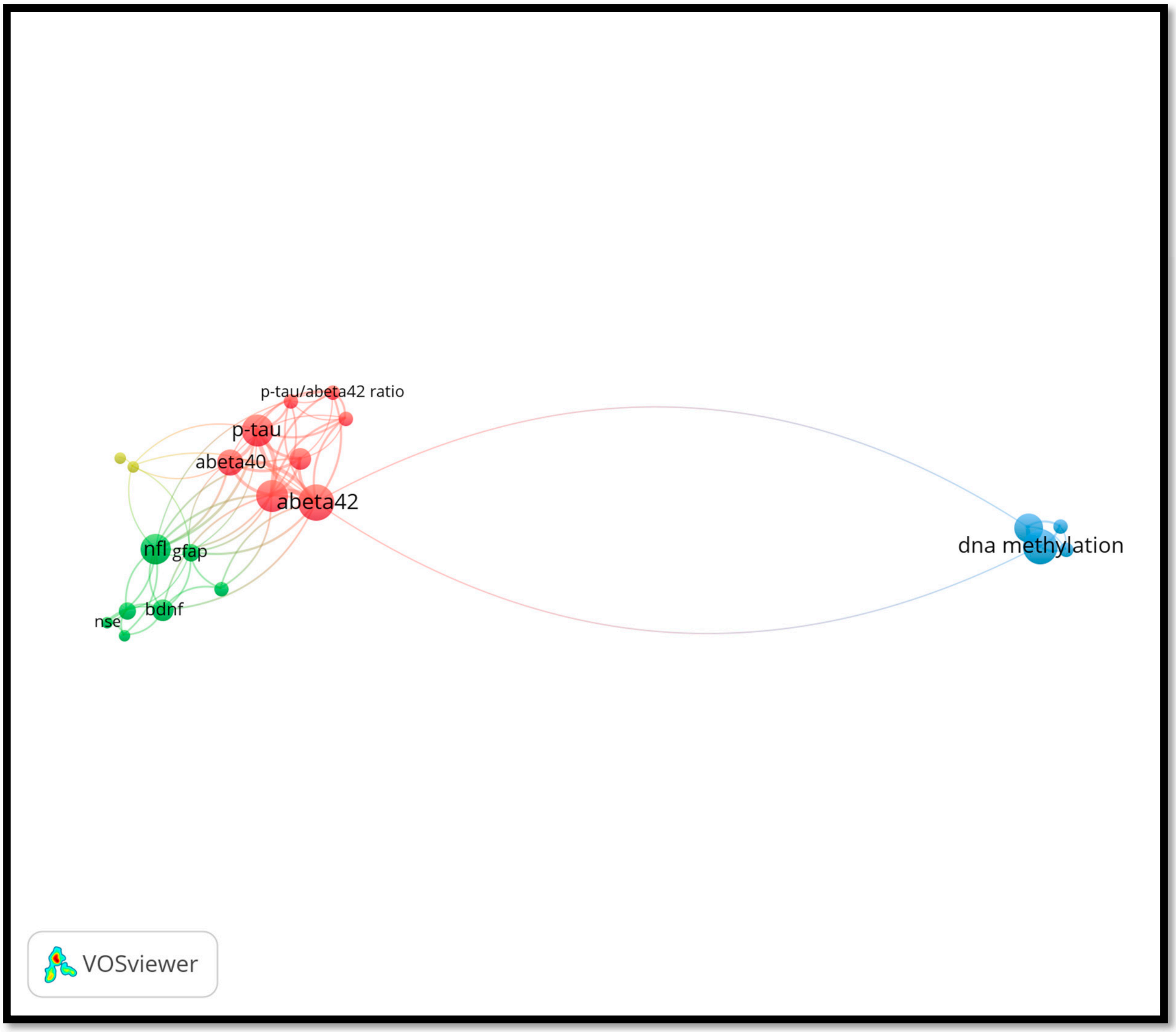

Figure S1. Biomarker-only co-reporting map, primary threshold (2 occurrences). Nodes represent harmonized biomarker terms; links indicate co-reporting within the same included study.

The pollutant-only map showed a more fragmented structure than the biomarker-only map. At the primary threshold (2 occurrences), 29 pollutant terms were retained and grouped into seven clusters when all components were considered. Metals and trace elements formed the densest component, whereas air-pollution terms formed a distinct component that remained visible when the occurrence threshold was increased.

The sensitivity analysis using a threshold of three (3 occurrences) retained 17 pollutant terms and four (4 occurrences) clusters. Metal and trace-element exposures and air pollution remained visible under the more restrictive threshold, whereas pesticide-related and organic-pollutant terms were more sparsely represented and more sensitive to the threshold change.

Dominant terms	Conservative descriptive label
Cadmium, lead, manganese, copper, iron, zinc, cobalt, selenium, arsenic, mercury, and other trace elements	Metal and trace-element exposure cluster
PM2.5, PM10, NO2, O3, SO2	Air pollution exposure cluster
Pesticides, fungicides, insecticides	Pesticide-related exposure terms
PAHs, PCBs, organophosphate pesticides	Sparse organic pollutant terms

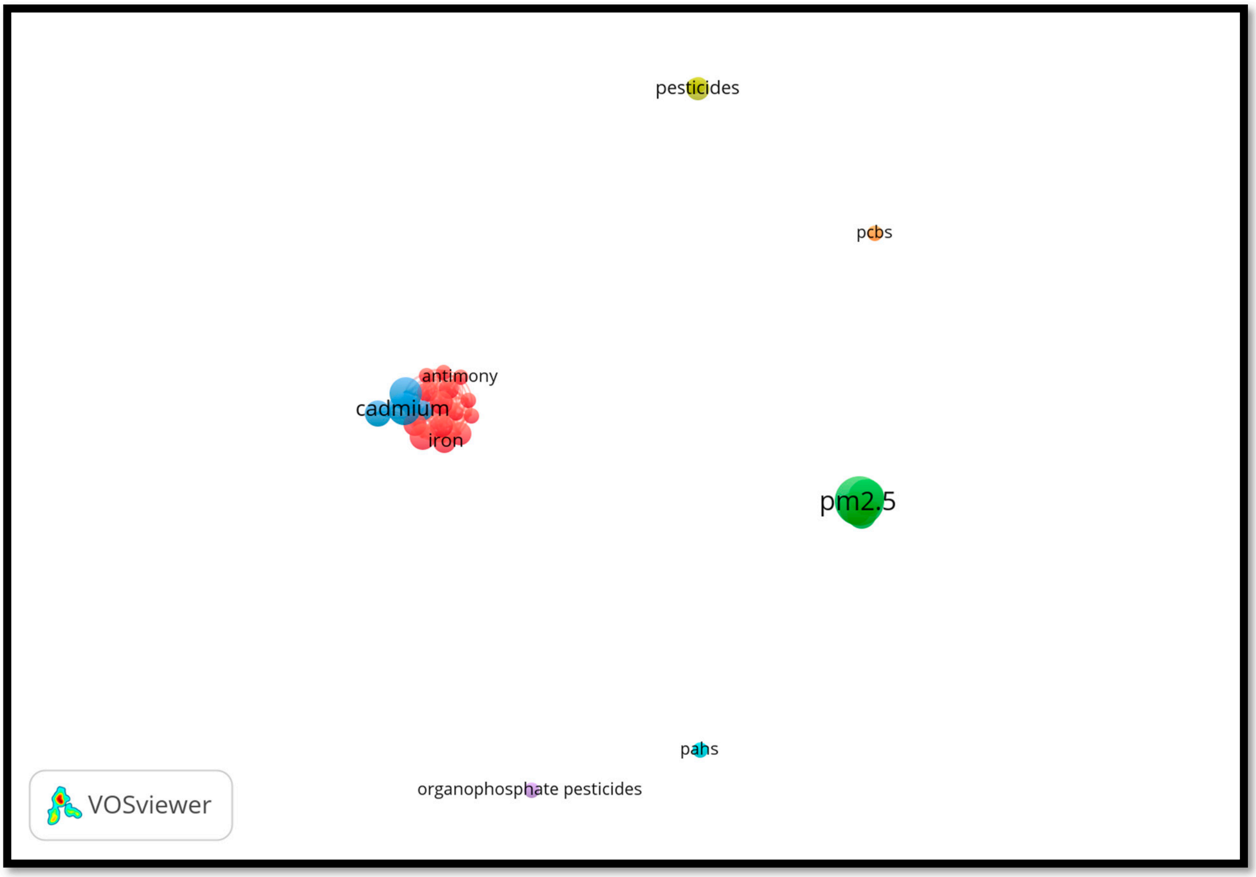

Figure S2. Pollutant-only co-reporting maps, primary threshold, all components. Nodes represent harmonized pollutant terms; links indicate co-reporting within the same included study.

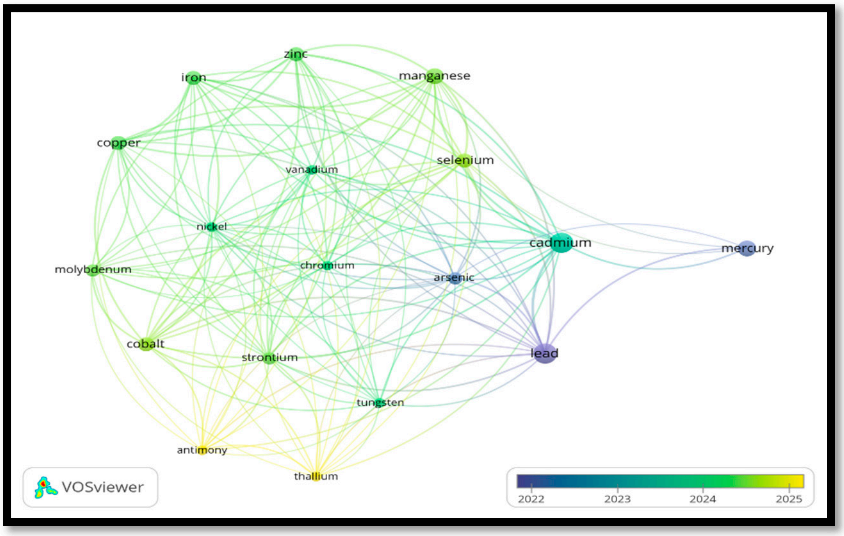

Figure S3. Pollutant-only co-reporting map, primary threshold, all Components, Cluster 1 (Cadmium).

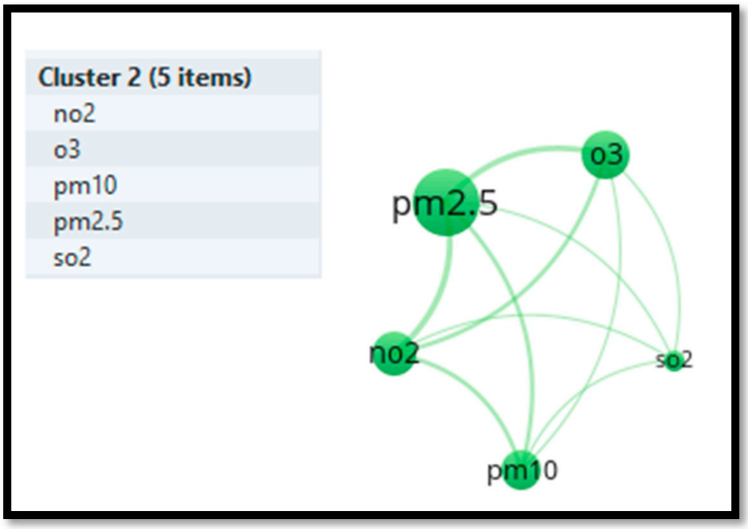

Figure S4. Pollutant-only co-reporting map, primary threshold (2 occurrences), all Components, Cluster 2 (PM2.5).

The combined pollutant-biomarker map was considered the most informative output, as it allowed exposure and biomarker terms to be visualized within the same co-reporting structure. At the primary threshold (2 occurrences), the map retained 50 items and four clusters. The most frequent and highly connected terms included Aβ42, p-tau, total tau, NfL, PM2.5, cadmium, and lead.

The sensitivity analysis using a threshold of three (3) retained 34 items and four (4) clusters. Although several low-frequency terms were removed, the main descriptive structure of the map remained visible. This threshold comparison therefore indicates persistence of the principal pollutant-biomarker co-reporting patterns rather than establishing robustness.

Based on the dominant cluster composition, the combined map suggested recurring study-level domains involving air pollution and amyloid/tau-related biomarkers, metal and trace-element exposures, neural injury/glial-neurotrophic biomarkers, and epigenetic biomarkers. These domains should be interpreted as descriptive structures of the reviewed literature rather than biological or causal pathways.

**Dominant composition**	**Conservative descriptive label**
PM2.5, NO2, O3, PM10 with Aβ42, Aβ40, p-tau and total tau	Air pollution-amyloid/tau co-reporting cluster
Cadmium, lead, manganese, copper, iron, zinc, cobalt, selenium, and related trace elements	Metal/trace-element exposure co-reporting cluster
NfL, GFAP, BDNF, S100B, alpha-synuclein, and related markers	Neural injury / glial-neurotrophic co-reporting cluster
DNA methylation, CpG sites, DMRs, EWAS, with links to selected organic/pesticide-related terms	Epigenetic biomarker co-reporting cluster

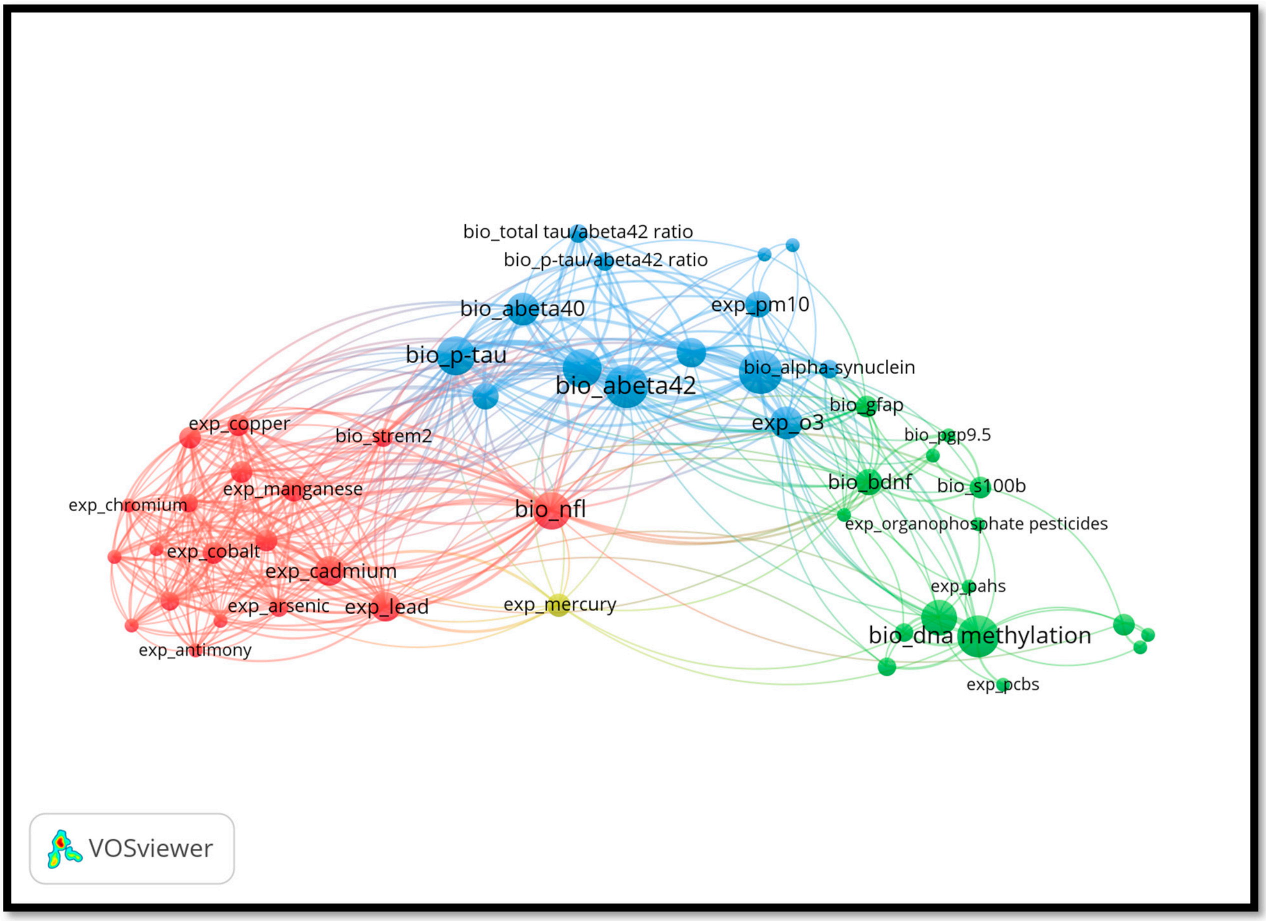

Figure S5. Exploratory pollutant-biomarker co-reporting map across the included studies, primary threshold (2 occurrences). Nodes represent harmonized biomarker and pollutant terms extracted from the review table. The links indicate that two terms were reported in the same study. Node size reflects occurrence frequency across studies, while link strength reflects the number of studies in which two terms were jointly reported.

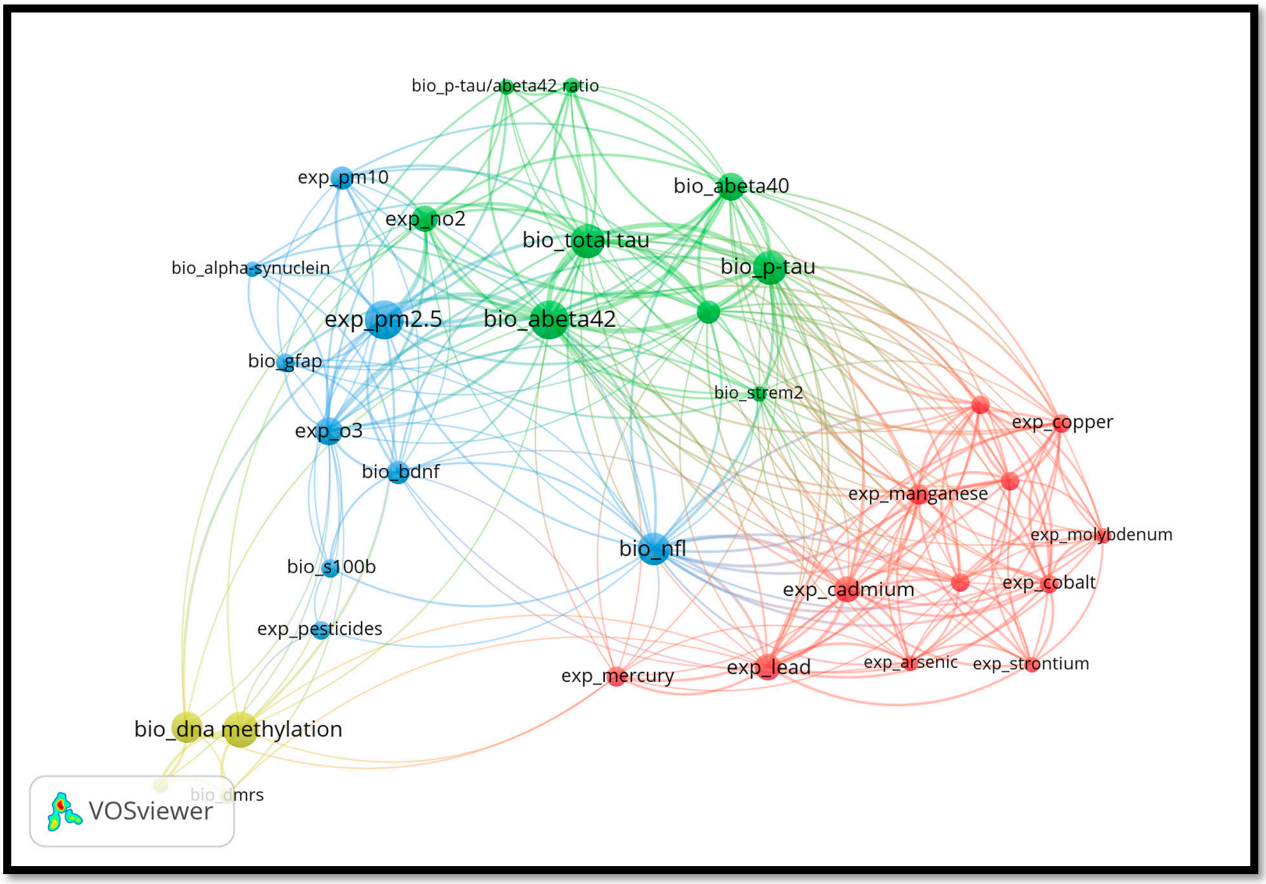

Figure S6. Sensitivity analysis: combined pollutant-biomarker co-reporting map using a more restrictive minimum occurrence threshold (3 occurrences). The main co-reporting structure was retained after removing lower-frequency terms.

The study traceability matrix provides an additional study-level view class-based co-reporting structure. Each bubble represents one study included in the review. A study appears in a cell when it reports at least one pollutant from the corresponding pollutant class and at least one biomarker from the corresponding biomarker class. Multiple bubbles within the same cell indicate that several studies contributed to that specific pollutant–biomarker combination.

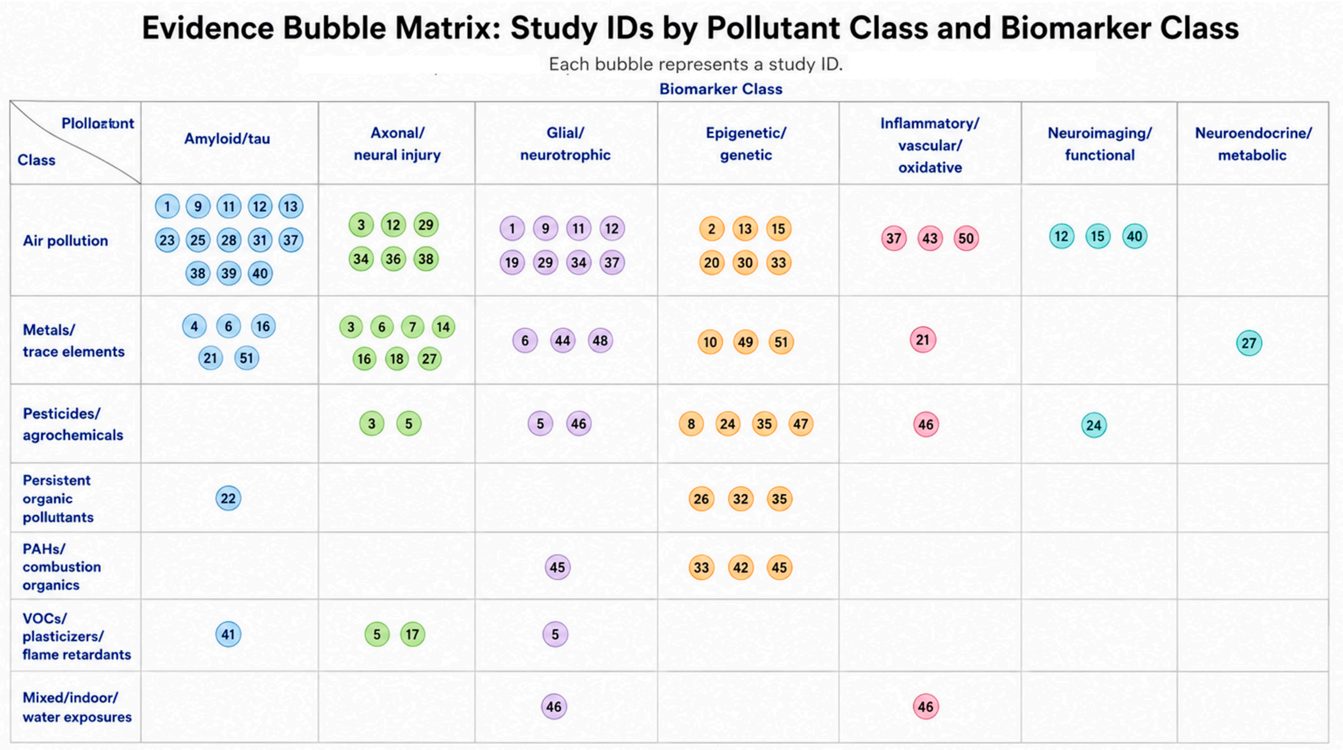

Figure S7. Study traceability matrix linking pollutant classes and biomarker classes.

The sensitivity analysis showed the main descriptive co-reporting patterns persisted when the minimum occurrence threshold was increased from two (2) to three (3) occurrences. As expected, fewer terms were retained, but the principal structure of the biomarker-only, pollutant-only, and combined pollutant-biomarker maps remained visible.

In the combined analysis, t four clusters were retained at both thresholds. Terms removed by the more restrictive threshold were mainly low-frequency or peripheral terms, whereas the main co-reporting domains remained visible. This comparison is interpreted as a descriptive sensitivity check on the persistence of the observed structure and not as evidence of statistical or methodological robustness.
